# From body scale ontogeny to species ontogeny: Histological and morphological assessment of the Late Devonian acanthodian *Triazeugacanthus affinis* from Miguasha, Canada

**DOI:** 10.1371/journal.pone.0174655

**Published:** 2017-04-12

**Authors:** Marion Chevrinais, Jean-Yves Sire, Richard Cloutier

**Affiliations:** 1 Université du Québec à Rimouski, Rimouski, Québec G5L 3A1, Canada; 2 UMR 7138-Evolution Paris-Seine, IBPS, Université Pierre et Marie Curie, Paris, France; New York Institute of Technology College of Osteopathic Medicine, UNITED STATES

## Abstract

Growth series of Palaeozoic fishes are rare because of the fragility of larval and juvenile specimens owing to their weak mineralisation and the scarcity of articulated specimens. This rarity makes it difficult to describe early vertebrate growth patterns and processes in extinct taxa. Indeed, only a few growth series of complete Palaeozoic fishes are available; however, they allow the growth of isolated elements to be described and individual growth from these isolated elements to be inferred. In addition, isolated and *in situ* scales are generally abundant and well-preserved, and bring information on (1) their morphology and structure relevant to phylogenetic relationships and (2) individual growth patterns and processes relative to species ontogeny. The Late Devonian acanthodian *Triazeugacanthus* *affinis* from the Miguasha Fossil-*Lagerstätte* preserves one of the best known fossilised ontogenies of early vertebrates because of the exceptional preservation, the large size range, and the abundance of complete specimens. Here, we present morphological, histological, and chemical data on scales from juvenile and adult specimens (scales not being formed in larvae). Histologically, *Triazeugacanthus* scales are composed of a basal layer of acellular bone housing Sharpey’s fibers, a mid-layer of mesodentine, and a superficial layer of ganoine. Developmentally, scales grow first through concentric addition of mesodentine and bone around a central primordium and then through superposition of ganoine layers. Ontogenetically, scales form first in the region below the dorsal fin spine, then squamation spreads anteriorly and posteriorly, and on fin webs. Phylogenetically, *Triazeugacanthus* scales show similarities with acanthodians (e.g. “box-in-box” growth), chondrichthyans (e.g. squamation pattern), and actinopterygians (e.g. ganoine). Scale histology and growth are interpreted in the light of a new phylogenetic analysis of gnathostomes supporting acanthodians as stem chondrichthyans.

## Introduction

Fish fossilised ontogenies are rare, especially in the Palaeozoic record [1] because the preservation of weakly mineralised skeletal elements of immature specimens requires exceptional fossilisation conditions. In extinct fish taxa, such as placoderms and acanthodians, the paucity of ontogenies is problematic because only ontogenies have the potential to inform about developmental patterns and processes in the past. Complete and articulated fossil fishes are rare in the Palaeozoic record, compared to isolated elements, which are abundant. Among these isolated elements, some, such as scales, record the ontogeny of the individual and thus provide a unique opportunity to describe their ontogeny [1, 2]. Fossil model organisms, for which both abundant individual isolated elements and complete specimens are known, are indispensable to describe the relationship between individual growth of isolated elements and species ontogeny [1].

Among early vertebrates, acanthodians have been recovered both from isolated elements and complete specimens from the Upper Silurian (423–419 million years ago) [3] to the Middle-Upper Permian (272–252 million years ago) [4]. Acanthodian species known from complete specimens are relatively rare compared to the number of taxa known solely from isolated scales [5–7]. Furthermore, only a few acanthodian ontogenies based on complete specimens have been discovered [1, 8]: one possible ischnacanthiform [*Nerepisacanthus denisoni* [3]], two diplacanthiforms [*Diplacanthus horridus* [9] and *Uraniacanthus curtus* [10]], one “climatiiform” [*Tetanopsyrus breviacanthias* [11]], one species of uncertain order *Lupopsyrus pygmaeus* [12]], and nine acanthodiforms [*Triazeugacanthus affinis* [13, 14], *Lodeacanthus gaujicus* [15–18], *Homalacanthus concinnus* [9], *Acanthodes bridgei* [19], *A*. *bronni* [20], *A*. *gracilis* [21], *A*. *lopatini* [22], *A*. *ovensi* [23]], and an acanthodiform indet. [24]. The rarity of complete specimens of acanthodians has been attributed to the micromeric nature of the dermal skeleton as well as the poor ossification of the endoskeleton [17, 25].

The micromeric dermal skeleton of acanthodians, composed of minute scales on the head and body, has been mainly described from adult specimens. Typically, acanthodian body scales are small, rhombic and composed of two tissue layers: a basal layer of bone and a layer of dentine [26, 27]. Some fundamental, histological differences have been reported among acanthodian groups such as the presence of osteocytes usually in the basal layer of the “climatiiform” scales and only sometimes recognizable in ischnacanthiform scales, the presence of vascular canals in the dentine layer of the ischnacanthiform scales and the presence of a superficial well-mineralised layer in some “climatiiform” and acanthodiform scales [5, 26, 28]. With regard to this disparity and based on tissue composition, Valiukevičius [28] defined four main types of scales characterising the four acanthodian orders; however, the phylogenetic status of these orders is questionable [29–31]. In addition, these scale types are only defined from adult specimens and from a few species. The *Nostolepis*-type (1) characterises “climatiiforms”: a thick basal plate of cellular bone, a crown of mesodentine, lacking well-mineralised, enamel-like tissue at the scale surface [32]. The *Diplacanthus*-type (2) characterises diplacanthiforms: a thick, vascularised basal plate of acellular bone, a crown of mesodentine, and no enamel-like tissue at the scale surface. The *Poracanthodes*-type (3) characterises poracanthid ischnacanthiforms: a basal plate of either acellular or cellular bone, a crown composed of either orthodentine, mesodentine or both, with a pore canal system opening superficially and on the neck, and no enamel-like tissue at the scale surface. Finally, the *Acanthodes*-type (4) characterises the acanthodiforms: a basal plate of acellular bone housing narrow vascular canals, a crown of mesodentine, and a well-mineralised, enamel-like tissue at the scale surface.

The four types of scales share a similar growth process, mainly characterised by the periodic superposition of bone and dentine layers around a single primordium. This mode of concentric growth is known as the “box-in-box” or “onion skin” pattern [5, 27]. With the exception of this growth process, scale ontogeny is poorly known mainly due to the destructive nature of histological methods, which explains why only a few adult specimens were analysed in previous studies. Non-destructive techniques such as nano-CT scanning [33] or synchrotron analysis [2, 34] are promising but their availability is still limited [35]. As a result, histological descriptions are rare and only the general structure of acanthodian scale tissues is known, including the presence of a basal layer of bone, a middle layer of dentine, with or without a superficial layer of well-mineralised enamel-like tissue. This structure is generalised among early gnathostomes but the diversity of histological and fine anatomical features and growth patterns and processes requires further examination. Therefore, ontogenetic data can be used to compare acanthodian scales to scales described in other early gnathostomes.

Among the well-documented fossilised ontogenies of Acanthodiformes recorded so far, the best documented is that of the middle Frasnian mesacanthid *Triazeugacanthus affinis* from the Escuminac Formation [13, 14], approximately 380 million years old. Phylogenetically, the Acanthodiformes are considered either as stem osteichthyans within polyphyletic acanthodians [30, 31], paraphyletic, as stem chondrichthyans [36], or as a paraphyletic sister-group of chondrichthyans and some acanthodians [37]. Given the debated phylogenetic position and status of the Acanthodiformes, new histological and ontogenetic information are, therefore, pertinent. Recently, the ontogeny of *Triazeugacanthus* was described from a large number of specimens [315 complete or almost complete specimens [38]], ranging in size from 4.51 to 52.72 mm, bridging larval, juvenile and adult stages, and showing exceptional preservation [13, 14]. This ontogeny demonstrates an increasing number of skeletal elements and a progressive extension of the squamation pattern as body size increases [14]. However, all previous observations on *Triazeugacanthus* ontogeny dealt with either changes in gross anatomy [9, 14] or chemical characterisation of anatomical elements [13], leaving the histological ontogenetic changes undescribed.

Taking advantage of this exceptional growth series of *Triazeugacanthus* specimens, we decided to use this species as a model to describe histological changes during ontogeny at the individual and species (among individuals) levels. The main objectives of this study were to (1) describe histological and morphological changes of scales during ontogeny, (2) investigate the relationship between these changes and the individual and species ontogenies, (3) characterise the squamation pattern during ontogeny, and (4) discuss phylogenetic implications of histological and ontogenetic changes.

*Institutional abbreviations*: MHNM, Musée d’Histoire naturelle de Miguasha, Parc national de Miguasha, Québec, Canada; MNHN, Muséum national d’Histoire naturelle, Paris, France; UPMC, Université Pierre et Marie Curie, Paris, France; UQAR, Université du Québec à Rimouski, Québec, Canada.

## Materials and methods

*Triazeugacanthus affinis* comes from the middle Frasnian Escuminac Formation (Miguasha, Québec, Canada) [39]. The studied material is housed in the MHNM collections and no permits were required for the described study.

Gross scale morphology was observed with a binocular microscope Leica MZ9.5 under water immersion, drawn using a camera lucida, and photographed with a Nikon D300. Samples for scanning electron microscopy (SEM) observations (six juveniles and one adult: S1 Table) were cleaned with 5% acetic acid, dried, glued on an aluminum stub and either sputter-coated with a thin layer of gold or not, according to the type of SEM analyses. Images were obtained with a Cambridge Instruments Stereoscan 260 SEM (Leica, Cambridge, UK) at UPMC. Elemental composition analysis was performed on two specimens [one juvenile (MHNM 03-398) and one adult (MHNM 03-1497); S1 Table] using an INCA X-sight (Oxford Instruments) energy dispersive X-ray spectrometer coupled to a JEOL 6460LV SEM at UQAR. Each spectrum was acquired for 100 seconds of lifetime (process time 5, spectrum range 0–20 keV, 2000 channels) at an accelerating voltage of 20 kV. Quantitative optimisation of the system was done using copper as a standard. Elements were automatically identified and quantified in weight by the INCA software and results were normalised to 100%.

**Fig 1:**
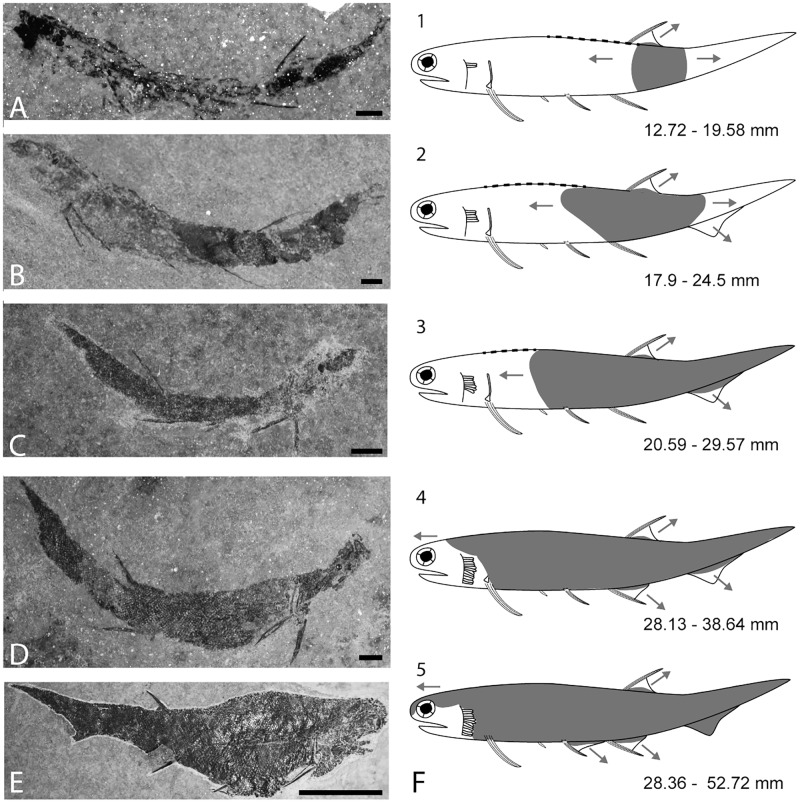
Development of the squamation pattern in *Triazeugacanthus affinis*. A-E: Ontogenetic stages with the corresponding squamation pattern schematically represented in F (1 to 5), respectively. A: Early juvenile MHNM 03-401. B: Early juvenile MHNM 03-2684. C: Late juvenile MHNM 03-259. D: Late juvenile MHNM 03-435. E: Adult MHNM 03-1497. F: Development of the squamation (grey zones) in relation to size ranges (not to scale). Dashed lines indicate the presence of median ridge scales. Arrows indicate the direction of the squamation progression along the body and in the fin webs. Dashed lines indicate the development of the median ridge scales. Arrows indicate the direction of the squamation progression along the body and fin webs. Scale bars: A-D = 1 mm; E = 10 mm.

Scale histology was analysed on 17 complete specimens: two early juveniles, in which squamation covers the region below the dorsal fin without extension to the pelvic fins (Fig 1A, 1B, 1F1 and 1F2), 11 late juveniles with incomplete squamation reaching the pectoral and pelvic fins (Fig 1C, 1D and 1F3), and four adults (Fig 1E and 1F5) (Table 1). The ontogenetic stages are defined following the criteria established by [14] (Table 1 and S1 Table). Blocks were restricted to the specimens using a BROT 380V diamond saw, embedded in stratyl resin containing 2% Luperox K1 catalyst, and sectioned into ca. 2-mm thick sections from the head to the caudal fin, *i*.*e*. perpendicular to the antero-posterior body axis, with a Leica 1600 saw microtome. These transverse ground sections (n = 207, with an average of 12 sections per specimens) were reduced to a final thickness of 150–200 *μ*m using abrasive disks, and then polished on both sides using alumina powder. Sections were glued with Araldite 2020 on glass slides and mounted with cover glass (Petropoxy 154 or Araldite 2020). Thin sections were observed under natural and polarised light with a binocular microscope (either Nikon Eclipse E600 POL or Leika DM LB2) and photographed with a microscope digital camera AmScope 10MP.

**Table 1:** Characteristics of the ontogenetic stages of the acanthodian *Triazeugacanthus affinis* based on [14].

	Larvae	Early juvenile	Late juvenile	Adult
Total length range (mm)	4.5–20.3	12.72–24.5	20.59–38.64	28.36–52.72
Squamation cover	no scale	dorsal fin region	from pectoral fins to caudal	complete
Squamation extent (% of total length)	0	<30	30–90	90–100
Cumulative number of skeletal elements at maximum size	13	16	21	23
Number of specimens	31	26	52	79

**Fig 2:**
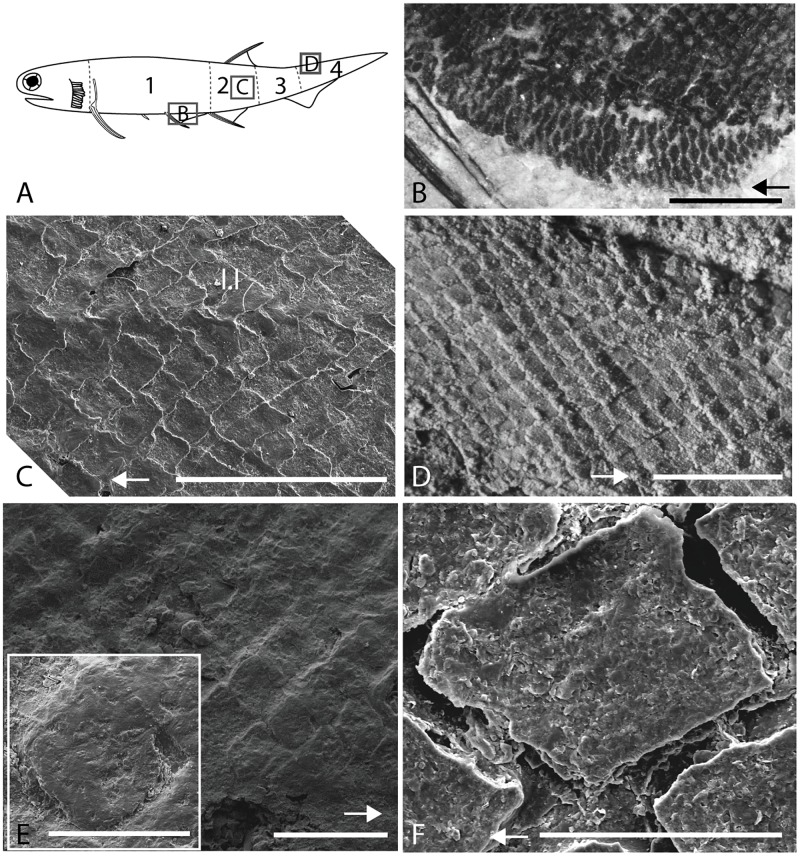
Variation of squamation along the body of *Triazeugacanthus affinis*. A: Schematic representation of an adult *Triazeugacanthus affinis* with position of the four body regions. Regions outlined in squares are detailed in B, C and D. B: MHNM 03-1550, detail of ventral scales. C: MHNM 03-1819, SEM showing scale alignment in the region below the dorsal fin (2). D: MHNM 03-1497, scale alignment in the caudal region whiten with ammonium chloride. E: juvenile MHNM 03-2631, SEM showing the organic layer (“epidermal cover”) covering the trunk scale ornamentation. F: juvenile MHNM 03-1819, SEM of a trunk scale. Arrows point forward. l.l, lateral line; body region 1, trunk; 2, dorsal-anal; 3, post-dorsal; 4, caudal. Scale bars: A = 5 mm; C = 500 *μ*m; D = 1 mm; E = 250 *μ*m; E (close-up), F = 100 *μ*m.

**Fig 3:**
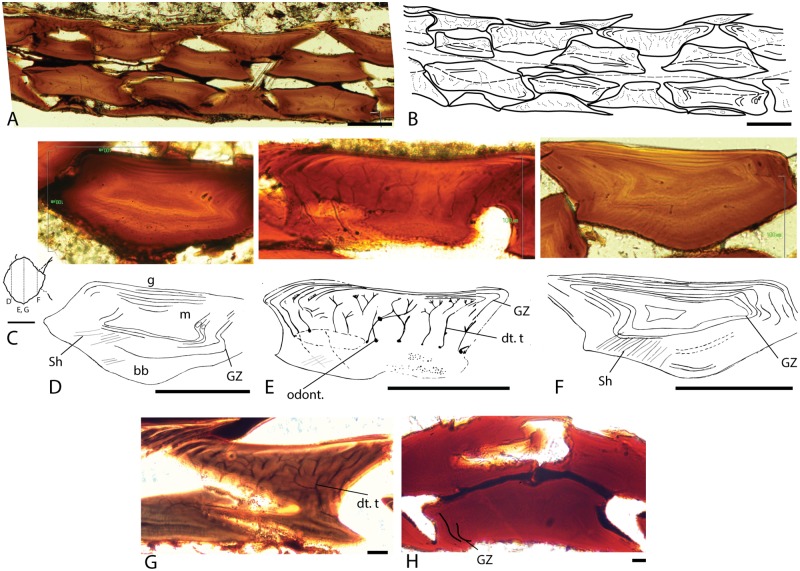
Transverse ground sections of *Triazeugacanthus affinis* scales in natural light. A-B: MHNM 03-2620, scale arrangement on both sides of the specimen showing the antero-posterior and lateral overlapping of the scales. B: MHNM 03-2620, interpretative drawing of A. The grey dashed line indicates the boundary between both sides; dark dotted lines indicate dentine tubules; dashed lines indicate the boundary between the crown and the basal plate. C: Diagram showing position of ground sections D-G in a body scale. D-F: Ground sections through the anterior, middle, and posterior levels of the scale and their interpretative drawings. D: MHNM 03-1817, the anterior region of the scale is mostly composed of acellular bone with embedded Sharpey’s fibers, a small, centrally located mesodentine layer, and thin layers of well-mineralised ganoine. E: MHNM 03-2620, the central region of the scale shows a basal plate of acellular bone, a thick middle region housing numerous ascending tubules and branched tubules, characteristic of the mesodentine, and a ganoine covering best visible in lateral regions, showing the growth zones. F: MHNM 03-1817, the posterior region of the scale is organised similarly to the anterior region of the scale. G: MHNM 03-2620, central region of the scale showing three dentine layers delimited by odontocyte cavities and tubules; each layer corresponds to a growth zone. H: juvenile MHNM 03-701, transverse section through the scales of a juvenile specimen showing a homogeneous histological composition. bb, bony base; dt. t, dentine ascending canals and tubules; g, ganoine layer; GZ, growth zone; m, mesodentine; odont., odontocytic cavities; Sh. f, Sharpey’s fibers. Scale bars: A-G, I = 100 *μ*m; G = 20 *μ*m; H = 10 *μ*m.

**Fig 4:**
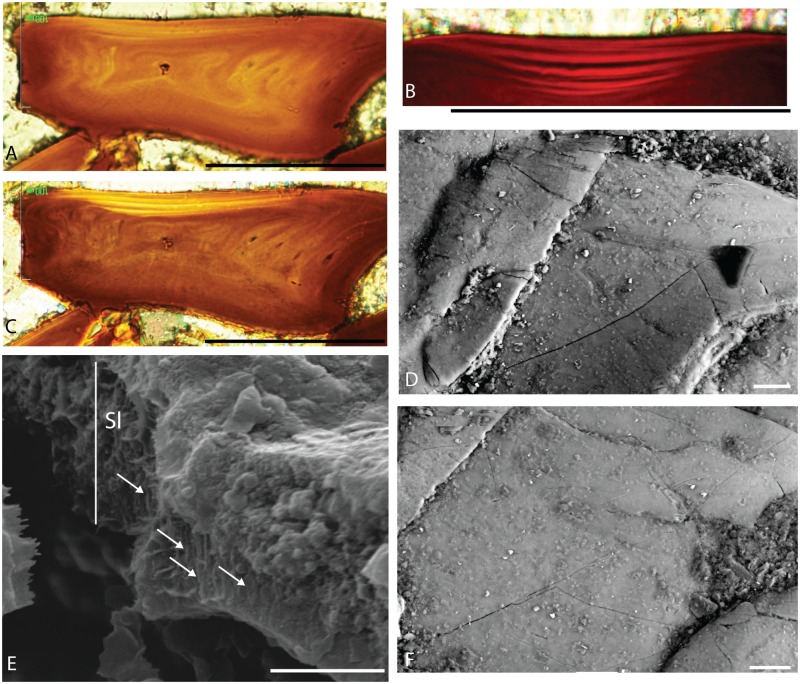
Superficial hypermineralised tissue of *Trizeugacanthus affinis* scales. A, C: MHNM 03-1817, ground section in natural (A) and polarised (C) light. B: MHNM 03-1817, close-up of the superficial multi-layered ganoine. D, F: MHNM 03-1460, SEM of the microtubercles of the ganoine surface. E: MHNM 03-1699, SEM showing the ganoine crystallites (arrows). Sl, superficial layer. Scale bars: A-C = 100 *μ*m; D and F = 20 *μ*m; E = 2 *μ*m.

**Fig 5:**
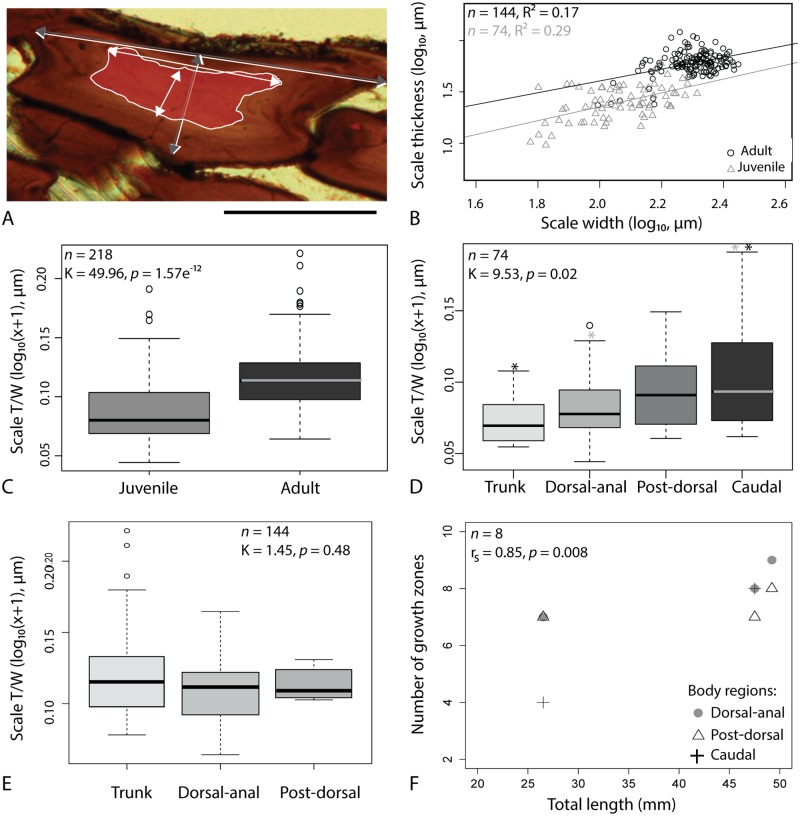
Individual and species ontogeny of *Triazeugacanthus affinis*. A: adult MHNM 03-1817, scale section of an adult specimen with superimposition (white lines) of the contours of a sectioned scale from a juvenile specimen (MHNM 03-701) [S2 Table for measurements (grey and white arrows)]. B: Scale thickness/width relationship in juvenile and adult specimens. C: Side-by-side boxplot showing thickness to width scale ratio in juvenile and adult specimens. D, E: Side-by-side boxplot showing thickness to width scale ratio in various body regions of juvenile D and adult E specimens. F: Number of growth zones per scale in function of the total length in various body regions of adult specimens. The regions from which measurements were taken are shown in Fig 2A. Asterisks refer to significant differences between two groups. Scale bar: A = 100 *μ*m.

The size and shape of juvenile and adult body scales of *Triazeugacanthus* (Figs 1–4) were measured on SEM and ground section images using Adobe Photoshop 14.0 (Fig 5A and S2 Table). Linear regressions between log_10_-transformed measurements of scale thickness and width were calculated for 74 juvenile (from two early and three late juveniles) and 144 adult scales (from three specimens). Mean thickness/width ratio of scales was compared between juveniles and adults as well as among body regions of juveniles and adults separately. Four body regions are defined based on previous descriptions of body regions in acanthodiforms [18, 19]. In *Triazeugacanthus*, they are delimited by fin positions, which allowed similar measurements among specimens (Fig 2A). The trunk region extends from the pectoral to the anal fins, the dorsal-anal region extends from the anterior limit of the anal fin to the posterior limit of the dorsal fin, the post-dorsal region extends from the posterior limit of the dorsal fin to the mid-length of the ventral web of the caudal fin and the caudal region extends from there to the posterior extremity of the caudal lobe. Comparisons of scales parameters among body regions were performed using the non-parametric Kruskal-Wallis test and the Tukey’s multiple comparisons test in R 3.0.2. Non-parametric tests were used because of the non-normality of the data.

The number of growth zones [“Wachstumszones” *sensu* [26]] in adult scales was recorded from images of ground sections. Data were taken in the dorsal-anal (region in which the squamation is initiated), post-dorsal and caudal regions. A Spearman correlation coefficient between the number of growth zones and the total length (TL) of specimens was calculated with R 3.0.2. The distances between two successive growth zones were measured in 16 adult scales from four adult specimens. Inter-growth zone distances have been plotted showing the growth variations among scales for “box-in-box” and superficial growth.

The data used to determine the squamation pattern (*i*.*e*. progression of scale coverage during growth) were collected from 188 specimens of *Triazeugacanthus*, with no or minimal taphonomic bias [14].

### Phylogenetic analysis

We included *Triazeugacanthus* and *Lodeacanthus* in a revised version of the data matrix recently published by Burrow et al. [37]. Burrow et al. [37]’s data matrix included 262 characters: 253 characters from Zhu et al. [36], two characters from Dupret et al. [40], and six original characters (including one uninformative character). The data matrix of Zhu et al. [36] included much of the data from Davis et al. [31] and Brazeau [30]. Our data matrix included the 261 characters used by Burrow et al. [37] plus six new characters (see S1 Appendix for the lists of characters and taxa as well as the data matrix). Twenty-nine characters and character states have either been rephrased, redefined, or repolarised (Characters 7–9, 11, 13, 18, 19, 26, 31, 51, 81, 104, 149, 160, 167, 177, 182, 190, 191, 195, 196, 209, 241, 242, 246, 252, 257, 258, and 260). Numerous modifications have been done throughout the matrix and more specifically on characters related to scales and histological features (Characters 8, 9, 11, 13, 260, 263–266); changes are given in the list of characters (S1 Appendix) and highlighted in the matrix (S1 File). Coding has been validated for numerous taxa with a special emphasis on *Homalacanthus* (M.C. and R.C., pers. observ., and S1 Fig), *Cheirolepis* [M.C. and R.C., pers. observ., S2 Fig and [41, 42]], *Miguashaia* (R.C., pers. observ.), *Gogonasus* (John A. Long, pers. comm.), and *Eusthenopteron* [R.C., pers. observ. and [43]]. *Lodeacanthus* has been coded based on [17] and [18] as well as direct observation on the material in the Latvian Museum of Natural History (Riga) (M.C., pers. observ.). Unknown data (“?”) represent 38.6% of the matrix, whereas not applicable codings (“-”) represent 18.5% of the total characters coded.

The data matrix (79 taxa and 267 characters) was analysed with PAUP version 4.0b10 [44]. The matrix was rooted on two outgroups (Galeaspida and Osteostraci). All characters were unordered and unweighted. We used a heuristic search; the branch-and-bound search did not yielded trees. Maxtrees was set at 100,000. ACCTRAN and DELTRAN options were used. We performed 1,000 bootstrap replicates using heuristic searches. We set the maximum number of trees saved for each random sequence addition to 50,000.

## Results

### Scale morphology

Body scale width ranges from ca. 0.08 mm (12 scales/mm; juveniles) to 0.20 mm (5 scales/mm; adults), and scale thickness ranges from ca. 30 *μ*m (juveniles) to 60 *μ*m (adults). Ventral scales are broader than trunk scales (Fig 2B).

Body scales are organised into oblique rows (Fig 2C and 2D) and are imbricated at one fifth of their length. The anterior region of a scale is overlapped by the elongated, posterior region of the preceding scale. The dorsal and ventral margins are anteriorly overlapped by the neighbour lateral scales, while they overlap the latter posteriorly (Fig 3A and 3B).

Trunk scales have a diamond-shape crown with a rounded angular anterior margin and a pointed posterior process. Such morphology is observed in juveniles and adults (Fig 2). The crown and the base, delimited by a poorly-developed neck (Figs 3, 4A, 4C and 5A), are of equal depth in juveniles and adults (Fig 3D–3F). The base is flat in juveniles and weakly convex in adults (Fig 3). The crown surface is flat in juveniles and adults (Fig 3D–3H). In juvenile specimens, the upper surface is smooth and homogeneous. In some ground sections of juvenile specimens (e.g. MHNM 03-210), this surface seems to be cover by a thin organic dark layer. Superficially, this layer covers scale boundaries (Fig 2E unlike in Fig 2F). We interpret this feature as potential remains of the epidermal cover (Fig 2E). In adult scales, the surface is ornamented with irregularly spaced microtubercles as revealed by SEM (Fig 4D and 4F).

### Scale histology

In juvenile 30 *μ*m-thick scales, ground sections reveal a homogeneous tissue composition (Fig 3H). Cell lacuna and tubules were not identified in the main tissue; these features characterize either acellular bone or dentine (Fig 3H). The upper surface is not covered with a well-mineralised tissue.

In contrast to juveniles, ground sections in adult 60 *μ*m-thick scales reveal three distinct tissues (Fig 3D–3G). The following three tissues are found from the deeper to the upper surface: a fibrillar and homogeneous tissue resembling acellular bone (= type 1), a fibrillar, thick and well-mineralised tissue (= type 2), and a thin, well-mineralised homogeneous tissue, organised into several, thin, superimposed layers (= type 3) (Fig 3D). None of the sections showed vascular canals. Type 1 tissue represents the main tissue forming the so-called basal plate. Neither osteocyte lacunae nor tubules are observed. This bone-like tissue is found at the base of the scale, and in the peripheral parts of the scale. In the lateral and posterior parts of the scales, longitudinal fibers oriented perpendicularly to the scale margin are similar to the Sharpey’s fibers and are therefore interpreted as collagen bundles (Fig 3D–3F). Type 2 tissue occupies mainly the central part of the scale, above the basal plate and is less developed in the anterior and posterior regions (Fig 3D, 3E and 3G). It is characterised by the presence of numerous tubules, often branched and running mostly perpendicular to the upper and lateral surfaces of the scales. The proximal extremity of the tubule exhibits cell lacuna. These tubules, interpreted as dentine tubules and cell lacunae, represent the space where odontocyt bodies were located in the scale. This tissue organisation with ascending tubules, putative horizontal connecting tubules and isolated odontoblasts is interpreted as mesodentine (Fig 3E and 3G). Up to three levels of mesodentine cells (*i*.*e*., odontocyt cavities and tubules) have been observed in some adult scales; each level corresponding to a growth zone (Fig 3G). There is a clear boundary between Type 2 and Type 3 tissues (Fig 4A–4C). Type 3 tissue, covering the upper surface of the scale, is well-mineralised and organised into several, thin, superimposed layers (Figs 3F and 4B). This tissue is birefringent and lacks cell lacuna and tubules. SEM observations reveal that this superficial layer is composed of parallel crystallites oriented perpendicularly to the scale surface forming rod-like structure (Fig 4E); this organisation suggests that collagen fibers are absent from the matrix. Microtubercles (ca. 2.5 *μ*m in diameter) of various shapes are irregularly distributed on the surface of this tissue (Fig 4D and 4F). Individual crystallites are too small to be clearly recognisable. The mineral of the scale tissues is chemically composed of carbonate-fluorapatite (calcium, phosphorus and fluorine; S3 Fig).

### Scale ontogeny

*Triazeugacanthus* scales show two distinct growth patterns: “box-in-box” and superpositional. The box-in-box” pattern is recognizable in the central part of the scales. It is composed of acellular bone and mesodentine, and reveals concentric addition of layers of bone and dentine matrix from the primordium towards the periphery, that are interpreted as growth zones (Fig 3D–3G). Growth zones appear as addition of thin dark and thick light layers (Fig 4B and 4C). This incremental pattern is clearer in the periphery (Fig 3E) than in the centre of the scales. Added to the “box-in-box” growth of the bony and dentine tissues, we observe the deposition of a well-mineralised tissue (superficial dark layer) at the upper surface of the scales (Fig 4B) with intervening dentine (superficial clear layers).

The relationship between the thickness and width of scales differs between juvenile and adult *Triazeugacanthus* (Fig 5B). Linear regressions are significant but have a weak coefficient of determination (R^2^ = 0.29, p-value = 3.489e^−7^ in juveniles; R^2^ = 0.17, p-value = 1.93e^−7^ in adults) revealing a high degree of intra- and inter-individual variation (Fig 5C). This difference is visible in ground sections, reflecting an ontogenetic change in the shape of the basal plate of the scales (Figs 3 and 5A). The thickness/width ratio differs significantly between juveniles and adults (Fig 5C: K = 49.96, p-value = 1.57e^−12^) and among body regions in juveniles (Fig 5D: K = 9.53, p-value = 0.02), whereas it is similar among body regions in adults (Fig 5E: K = 1.45, p-value = 0.48). Pairwise comparisons of this ratio among body regions of juveniles show significant differences between the trunk and caudal regions (p-value = 0.007) and between the dorsal-anal and caudal regions (p-value = 0.04) (Fig 5D).

### Individual and species ontogeny

Individual ontogeny, *i*.*e*. the growth of a single individual, is recorded from the growth zones observed in scale sections. The bony tissues display zones with relatively fast (clear) and slow (dark) growth (Fig 3D–3F). Two growth zones are already present in the scales of the smallest (youngest) available juvenile specimen MHNM 03-701 (33.18 mm TL; Fig 3H). In all adult scales studied, the number of growth zones range from three to eleven clear zones (Fig 3D–3F). The strong positive correlation (*r*_*s*_ = 0.85, p-value = 0.008) between the number of growth zones in scales and the total length of the adult specimens reveals a clear relationship between individual growth (number of growth zones) and species growth (body size of specimens) (Fig 5F). Variation in the thickness of growth zones associated with the “box-in-box” growth indicates non proportional deposition of these tissues, even within individuals (S4 Fig). In contrast, growth of superficial layers is more constant and displays weaker variation in the thickness of successive layers than observed in the scale thickness (S4 Fig).

### Squamation pattern

Data from 188 complete *Triazeugacanthus* specimens of various sizes and ontogenetic stages allowed accurate description of the squamation pattern (Fig 1). From 12.72 to 19.58 mm TL, juvenile specimens possess a single, small patch of scales on the body, located below the dorsal fin spine, suggesting that scales initiate first in this region (Fig 1A and 1F1). This patch develops at the mid-height of the body, but precise position in earliest stages of squamation is difficult to assess because the body outlines are poorly defined (Fig 1A). Simultaneously, scales of the dorsal fin web and of the caudal lobe start to develop; scales are added proximo-distally and organised in multiple rows along the base of the fins. Anteriorly to the progression of this main body squamation, there is a median row of paired small scales that developed on the dorsal edge of the body from posterior to anterior forming a dorsal mid-line. Their position clearly anterior to the anterior edge of the main squamation and their shape show that they likely develop earlier than the scales from the main squamation (Fig 1 dashed line and S5 Fig). In juvenile individuals between 17.90 and 24.50 mm TL, scales cover most of the posterior half of the trunk, which indicates that squamation extends anteriorly and posteriorly from the initial region (Fig 1B and 1F2). The squamation progresses first dorsally then ventrally. In 20.59 to 29.57 mm TL specimens, the squamation has expanded anteriorly towards the head, dorsally towards the dorsal fin web and posteriorly towards the caudal lobe (Fig 1C and 1F3). In individuals between 28.13 and 38.64 mm TL, the caudal fin and the posterodorsal part of the head region are scaled (Fig 1D and 1F4). In adult specimens from 28.36 to 52.72 mm TL, the squamation is complete on the body, covers the head dorsally, and scales are present on the pelvic, anal, dorsal and caudal fin webs (Fig 1E and 1F5). None of the adult specimens, even the largest one (52.72 mm-TL, MHNM 03-1107), display scales associated with the pectoral and intermediate spines. Dorsal mid-line scales show a ridge at the mid-width of the scale (S5 Fig) suggesting that the paired small scales may have fused during development to form a dorsal mid-line of median ridge scales as observed in some basal osteichthyans [45–47].

In adult *Triazeugacanthus*, 114 scales are counted on the longitudinal row at mid-height of the body, along the antero-posterior axis from the scapula to the posterior extremity of the caudal fin. At the deepest part of the body (*i*.*e*. between the dorsal and anal fins) a total of 15, 17 and 21 scale rows are present in early juveniles (incomplete squamation), late juveniles (nearly complete squamation) and adults, respectively.

### Phylogenetic analysis

**Fig 6:**
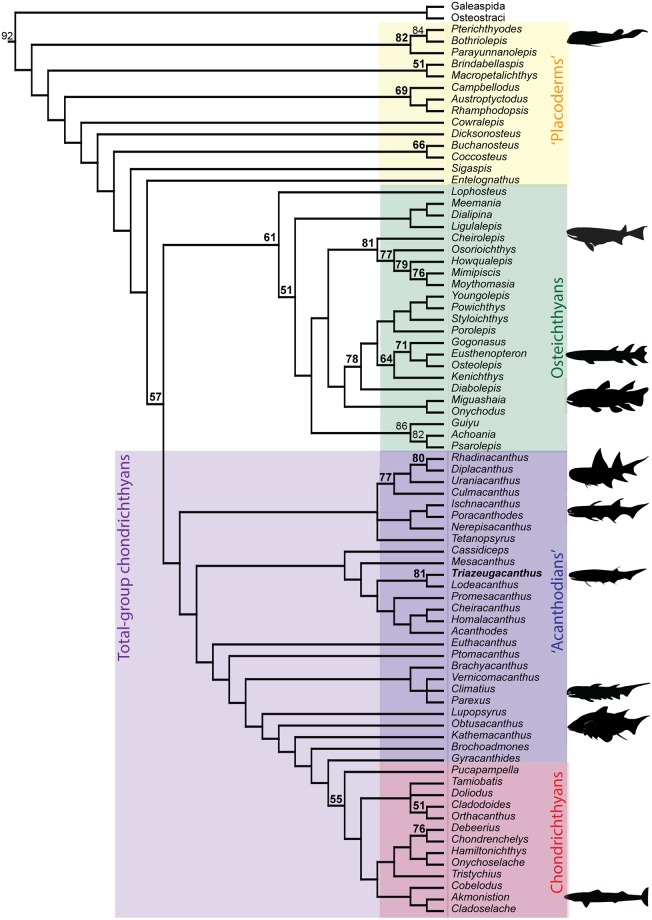
Phylogenetic relationships among early gnathostomes. 50% majority rule consensus tree based on 10,000 trees at 711 steps (79 taxa, 267 characters). Numbers on branches show percentage bootstrap support.

Burrow et al. [37] published the most recent phylogenetic analysis to investigate the phylogenetic status of acanthodians in relation to gnathostome interrelationships. Our phylogenetic analysis of the revised data matrix (see S1 Appendix and S1 File) provided 100,000 equally parsimonious trees at 711 steps (CI = 0.3952; RI = 0.7949; rescaled CI = 0.3142). Interrelationships among acanthodian taxa as well as the phylogenetic position of acanthodians among gnathostomes are recovered in the strict (S6 Fig), Adams (S6 Fig) and 50% majority rule consensus (Fig 6) trees; uncertainties in the topologies come primarily from placoderm and basal osteichthyan interrelationships (Fig 6, S6 Fig).

As proposed by Brazeau [30], Davis et al. [31] and Burrow et al. [37], the “Acanthodii” are considered to be paraphyletic with respect to the Chondrichthyes. Ischnacanthiformes and Diplacanthiformes are basal stem taxa. *Triazeugacanthus* is considered as the sister-group of *Lodeacanthus* in a monophyletic Acanthodiformes. The *incertae* *Euthacanthus*, the *incertae* gnathostome *Ptomacanthus*, climatiids, the Lockhovian MOTH *Lupopsyrus*, the so-called “putative stem chondrichthyan” *Obtusacanthus* and *Kathemacanthus*, the Lockhovian MOTH *Brochoadmones*, and the Early Carboniferous *Gyracanthides* are considered as stem chondrichthyans. It takes 22 supplementary steps to move the acanthodiforms, ischnacanthiforms and diplacanthiforms as stem-osteichthyans in a topology similar to that proposed by Davis et al. [31].

## Discussion

Previous to this study, the scale structure of *Triazeugacanthus* was poorly known. Here, owing to the large number of *Triazeugacanthus* scales sectioned we provide accurate description of the histology and spatial organisation of the scale tissues and their changes during ontogeny. The growth series of *Triazeugacanthus* *affinis* shows clearly that (1) the histological composition of the scales increases in complexity during ontogeny from a single, homogeneous tissue in juveniles to three tissues in adults including the well-mineralised superficial layer, (2) the central part of the scale grows according to a “box-in-box” pattern and the superficial part grows by superposition of well-mineralised layers, (3) the shape of the scales varies among body regions and ontogenetic stages, (4) the number of growth zones in scales is positively correlated with the total length, and (5) the squamation is initiated in the mid-body region, at the level of the dorsal fin, then spreads bidirectionally. In addition, this histological investigation of *Triazeugacanthus* contributed to the reinterpretation of certain scale characteristics included in a revision of the phylogenetic analysis of gnathostomes with a special emphasis on acanthodians.

In order to discuss the histological characters observed in *Triazeugacanthus*, we will first discuss some of the major results from the phylogenetic analysis (S1 Appendix). The original objective of this re-analysis was to include two new taxa in Burrow et al. [37]’s data matrix, *Triazeugacanthus* and *Lodeacanthus* [a species suggested as closely related to *Triazeugacanthus* [12, 18]], in order to discuss the histological observations in a phylogenetic context (S1 Appendix). As a result of our modifications, the new consensus topology differs slightly from Burrow et al. [37] but agrees with the overwhelming tendency to consider acanthodians as paraphyletic.

Only four recent phylogenetic analyses addressed specifically the phylogenetic status of acanthodians [12, 30, 31, 37]. In three of these analyses, acanthodians are recognised paraphyletic; the acanthodian paraphyly is also recognised in studies focusing on gnathostome interrelationships with a special emphasis on placoderms [36, 48]. In both Brazeau [30] and Davis et al. [31], some acanthodians are considered as stem gnathostomes and stem chondrichthyans while acanthodiforms are stem osteichthyans. Burrow et al. [37] proposed that acanthodians are solely stem chondrichthyans; this conclusion had already been reached in part prior to the phylogenetic analysis in Zhu [36] and Burrow and Rudkin [3]. Burrow and Rudkin [3] had suggested that acanthodians were either stem chondrichthyans or the monophyletic sister-group of chondrichthyans. Chondrichthyan affinities were also suggested by Brazeau and Friedman [49] and Giles et al. [50]. On the other hand, Hanke and Davis [12] suggested a monophyletic Acanthodii sister-group to osteichthyans and Dupret et al. [40] suggested a monophyletic Acanthodii sister-group to chondrichthyans; the monophyly had repeatedly been suggested for more than 40 years (e.g. [25, 29, 51, 52]).

The main differences between our topology and that reported by Burrow et al. [37] come from the order of “acanthodian” taxa along the stem. In our topology, climatiids are closer to putative chondrichthyans (*i*.*e*. *Brochoadmones*, *Kathemacanthus*, *Obtusacanthus*, and *Lypopsyrus*) rather than being at the base of the clade [37]. Furthermore, as suggested by Brazeau and Friedman [49], *Ptomacanthus* is closer to the chondrichthyans than the main acanthodian clade.

In addition to the phylogenetic characters, we compiled S4 Table from previously described scales in 43 acanthodians (including putative chondrichthyans), four early chondrichthyans and four early osteichthyans.

### Morphology and histology of acanthodian scales

Typically, acanthodian scales are rhombic with overlapping of neighbour scales, and organized in oblique rows [character 14(1)]. Each scale being attached to the neighbour scales and to the underlying dermis by Sharpey’s fibers [5]. Their crown surface is often ornamented and their neck is clearly constricted [5] [character 11(1)]. In contrast to the general condition, the scales of *Triazeugacanthus* are diamond-shaped, they only slightly overlap, the crown surface is smooth with subtle microtubercles, the neck is poorly developed and the base is convex [in contrast to the flat base reported by Burrow and Young [7]] [characters 12(1), 13 (0)]. The number of flank scales per millimetre in *Triazeugacanthus* fits within the acanthodiform range (2–16 scales/mm) which is higher than in most other acanthodians (S4 Table).

Acanthodian scales are commonly described as composed of two tissues: a deep basal plate formed either by acellular or cellular bone and a crown region composed of multiple layers of dentine (S4 Table) [53] (character 4). In most acanthodians, the basal plate is primarily acellular or occasionally cellular, whereas the crown is composed of mesodentine with a few exceptions in which it is composed of orthodentine (S4 Table). In previous descriptions (S4 Table), the composition of the crown was frequently referred simply as dentine. The distinction between the two types of dentine found in acanthodians (orthodentine and mesodentine; the semidentine being restricted to placoderms; character 5) takes into account the relative position of the cell bodies or odontoblasts. Mesodentine is characterized by cell bodies embedded within the dentine matrix, whereas in orthodentine all cell bodies are located at the matrix surface, along the walls of vascular canals or pulp cavities [53]. The crown of *Triazeugacanthus* scales is clearly composed of mesodentine. Narrow vascular canals, even if they are absent in the crown, could be present in the basal plate of some acanthodiforms. In *Acanthodes* *bronni*, the scale structure is characterised by the presence of dentine tubules in the crown and cell processes in the basal plate [54], with a clear boundary between the two zones [26]. However, in acanthodiforms (in which scales are small in comparison to other acanthodians), the canal network at the base is reduced to thin tubules where remnants of a vascular plexus are present in the centre of the scale (such as in *Halimacanthodes*) [55]. This absence of nutrient supply through vascularisation could explain the presence of mesodentine, with a rich network of long tubules. *Triazeugacanthus* shows the typical *Acanthodes*-type defined by Valiukevičius [28]; this condition could be considered derived among acanthodians.

Based on the topology, there seems to be no strong phylogenetic signal associated with the presence of the different types of dentine among acanthodian taxa. This is in contrast to Davis et al. [31] who suggested that mesodentine was found at the base of a large clade [“acanthodians” + [[“acanthodians” + chondrichthyans] + [“acanthodians” + osteichthyans]]], and also Brazeau [30], who suggested that mesodentine was only characteristic of the clade [“acanthodians” + osteichthyans]. The type of dentine might be phylogenetically informative at a higher phylogenetic level, but the coding of this character is certainly in need of revision.

Among acanthodians, the scale surface is either smooth (unornamented) or ornamented with longitudinal or radiating ridges, which cover either the complete surface of the scales or are limited to the anterior edge. At the base of the chondrichthyan total-group, scales have primarily a ridged crown surface as the main condition in ischnacanthiforms (some species do have a smooth surface S4 Table) and diplacanthiforms. Based on our topology, the smooth surface is most likely independently derived in acanthodiforms; the smooth surface also occurs in some climatiids and early ischnacanthids [56]. With the exception of *Lupopsyrus*, so-called putative chondrichthyans and *Gyracanthides* have an ornamented scale surface owing to their polyodontode condition [character 8(0)].

The smooth scales of acanthodians are occasionally covered by minute superficial microtubercles [55, 57, 58]. Such microtubercles are also present on the scale surface of some osteichthyans (e.g. *Polypterus* and *Lepisosteus*) [58–60]. In adult *Triazeugacanthus*, the crown surface is ornamented with microtubercles, similar to those described on the crown surface of the scales in three acanthodiform taxa [*i*.*e*. *Acanthodes* sp., acanthodiform indet. [61] and *Halimacanthodes ahlbergi* [55]] (S4 Table). The presence of these microtubercles has been considered as characteristic of ganoine [27, 58, 60, 62–65]. However, in order to conclusively show the presence of ganoine in *Triazeugacanthus* histological and SEM investigations were necessary.

The crown surface of acanthodian scales is generally covered by a hypermineralised tissue (S4 Table) (characters 264, 265, and 266). The identification of this hypermineralised tissue is still controversial since it was interpreted either as enamel, enameloid or ganoine [60, 65, 66]. Enamel is a homogeneous tissue that does not include collagen fibrils nor cells during its development [65]. The mature enameloid differs from the enamel by the presence of a loose network of collagen fibres resulting in less ordered mineral crystals [53]. The mature ganoine is a non-collagenous tissue and differs from dental enamel by the presence of multiple layers [53, 65]; single-layered ganoine is accepted by some authors [60]. Ganoine is considered as homologous to enamel by some others [42, 67, 68]. Ganoine is known unambiguously in early actinopterygians [42, 60, 69] as well as extinct and extant polypterids and lepisosteids [53, 60]. As a result, the presence of ganoine has been frequently considered as an actinopterygian synapomorphy [64, 65, 70, 71]. However, the identification of ganoine in acanthodians has been suggested to invalidate this character as an actinopterygian synapomorphy [60, 66]. Only few reports have suggested the presence of ganoine in acanthodians, and its proper identification remains questionable. In one species of acanthodians, referred to *Acanthodes* sp. 4 [61], ganoine was identified solely on the presence of superficial microtubercules. Burrow et al. [55] identified the presence of birefringent enameloid in the scales of *Halimacanthodes ahlbergi* based on both the presence of superficial microtubercules and the multi-layered nature of the tissue, a description that corresponds to ganoine rather than to enameloid as the latter is not layered [65]. Richter and Smith [60] suggested the presence of enamel-like ganoine in a scale identified as “*Cheiracanthoides*” sp. based on the multi-layered superficial tissue which lacked the microtubercules. In adult *Triazeugacanthus* scales, the superficial layer consists of crystallites organised perpendicularly to the scale surface. However, these crystallites were too small to be clearly recognisable, a condition similar to that observed in the Late Silurian “*Cheiracanthoides*” sp. [60]. Richter and Smith [56] mentioned that an unclear or non-existent boundary between the dentine and the superficial hypermineralized layer seems to be the commonest condition in acanthodian scales. This unclear boundary is an additional argument that these authors used to question the clear identification of ganoine in acanthodians. However, the boundary between the mesodentine and the hypermineralised tissue in the scales of *Triazeugacanthus* is clear and distinct. Therefore, we interpret the superficial hypermineralised tissue on the body scales of *Triazeugacanthus* as ganoine based on the presence of microtubercles at the crown surface, the multi-layered structure, the perpendicular orientation of the mineral crystallites to the scale surface suggesting that collagen fibers are not present in the matrix [72], and the clear boundary with the mesodentine. As far as we know, this makes it the first unambiguous identification of ganoine in an acanthodian.

As proposed by Valiukevičius [28] and Valiukevičius and Burrow [32], various groups of acanthodians shared histological similarities of their scales. These similarities led to the recognition of four types of scales: the *Nostolepis*-type (1) (in “climatiiforms”), the *Diplacanthus*-type (2) (in diplacanthiforms), the *Poracanthodes*-type (3) (in ischnacanthiforms), and the *Acanthodes*-type (4) (in acanthodiforms). Based on our topology, a sequence of *Poracanthodes*-type—*Diplacanthus*-type—*Acanthodes*-type—*Nostolepis*-type would form a transformation series precursor to the polyodontode scales found in putative chondrichthyans *Gyracanthides* and chondrichthyans. However, the phylogenetic distribution of some of the different characteristics (acellular or cellular base, vascularised or avascularised base, orthodentine or mesodentine, presence or absence of enamel-like tissue) defining these four types is not congruent with our topology. The distribution of dentine and hypermineralized tissue types is homoplastic; thus suggesting that these scale types might be informative to identify acanthodian groups but are poorly informative phylogenetically.

One would expect that closely related species are more likely to share similar histological composition. *Triazeugacanthus* is considered as the sister-group of *Lodeacanthus* [12, 15, 18 this study] which is reflected in part by some histological similarities (e.g. mesodentine and acellular bony base) (S4 Table) [18]. However, major histological differences are also observed: *Lodeacanthus* scales show the presence of vascular canals in the crown and a layer of hypermineralised superficial tissue, while *Triazeugacanthus* scales lack vascular canals but have multi-layered ganoine.

At a higher phylogenetic level, a few histological scale characters are suggestive of phylogenetic affinities. For example, the presence of ganoine in *Triazeugacanthus* would suggest a phylogenetic affinity with actinopterygians. However, as we have demonstrated it would require minimally 22 steps to place the acanthodiforms [and related taxa as suggested by Brazeau [30] and Davis et al. [31]] as the sister-group to osteichthyans, while it would require either 26 or 39 additional steps to place *Triazeugacanthus* alone as the sister-group of actinopterygians or basal osteichthyans, respectively. The presence of ganoine alone cannot support a close relationship between some acanthodians and actinopterygians. An additional character shared by *Triazeugacanthus* [14, 73], numerous acanthodiforms, and osteichthyans is the presence of three pairs of otoliths [74, 75]. Schultze [74, 75] considered the presence of three pairs of otoliths as a synapomorphy shared by acanthodians (acanthodiforms) and osteichthyans. However, the rarity of information concerning the presence of this character would not have an impact on the resolution of the tree. For instance, none of the osteichthyan taxa used in this phylogenetic analysis could be coded for the presence of otoliths while *Triazeugacanthus*, *Homalacanthus*, *Mesacanthus*, and *Acanthodes* would have been coded as sharing three pairs of otoliths [75]. Similarly the paucity of endochondral information for acanthodian taxa limits potentially our phylogenetic resolution of this group [76].

### Assessment of individual and species ontogeny from scale growth pattern

The abundant material of *Triazeugacanthus* allowed us to evaluate both ontogenetic changes within a single individual by looking at scale growth, and ontogenetic changes among individuals by comparing morphology and histology along a size series. Individual growth of *Triazeugacanthus* had already been alluded by Gagnier [77] who mentioned two distinct orders of growth lines in a saccular otolith. On the other hand, *Triazeugacanthus* ontogeny had already been evaluated among individuals by looking at body shape changes [9], size changes [14], squamation pattern [14], and mineralisation pattern [13]. In the present investigation, we intended to combine all ontogenetic information.

Scale growth process varies among acanthodians. Most acanthodian scales grow by addition of concentric layers of mesodentine (or orthodentine) forming growth zones which results in the classical “box-in-box” (or concentric “onion skin”) pattern [5] [character 9 (1)]. This “box-in-box” pattern has been reported in the acanthodids *Acanthodes bridgei*, *A*. *lopatini*, and *A*. *lundi*, the cheiracanthid *Homalacanthus concinnus* (S1 Fig), all mesacanthids, some Diplacanthiformes, Climatiiformes and Ischnacanthiformes [5, 19] (S4 Table). In addition to this “box-in-box” growth, the superficial region of the scale might thicken by the superimposition of ganoine layers as described in *Triazeugacanthus*. In living polypterid and lepisosteid actinopterygians, the first layer of ganoine matrix is deposited on the scale surface only when the basal layer cells of the epidermis become in close contact with the upper layer of either the dentine (polypterids) or bone (lepisosteids) matrix [67, 68, 78]. In lepisosteids and polypterids, the epidermal cells partially retract periodically from the scale surface allowing the mesenchymal cells to invade the space left free between the epidermal basal cells and the scale, in particular in the lateral parts. We observed the same growth pattern at the surface of *Triazeugacanthus* scales. Therefore, *Triazeugacanthus* scales show two growth modes: (1) the “box-in-box” growth for mesodentine and basal bone and (2) the superpositional growth for ganoine.

The “box-in-box” growth pattern has frequently been considered as an acanthodian synapomorphy [5, 25, 79] or defining a sub-inclusive acanthodian clade [76]. Although the “box-in-box” pattern is recognised by most authors as a generalised condition among acanthodians, there are some disagreements in terms of recognising this growth pattern as either characteristic of monodontode (monoodontode) or polyodontode scales. Monodontode scales represent scales composed of a single unit, the odontode (vascular supply takes place through basal canals and/or neck canals) [80]; they either grow, or not, by concentric addition of dentine and bone layer. On the other hand, polyodontode scales correspond to a complex of fused or apposed odontodes (*i*.*e*. independent single units) lying on a bony basal plate and showing areal or appositional growth [2, 80–85]. Considering the “box-in-box” of acanthodians, Ørvig [80] referred to these scales as odontocomplex without mentioning if they were either monodontode or polyodontode. The “box-in-box” scales of acanthodians and early actinopterygians (e.g. *Cheirolepis canadensis* S2 Fig) are considered as monodontode (*contra* [30, 31, 37]) because of the presence of a single primordium per scale and the non-independence of individual growth layers. We suggest that each layer does not represent a single, separate unit (*i*.*e*. each layer is not a separate odontode) but rather an accretion around an initial unit; however, 3D microanatomical and histological data would be necessary to clarify this issue (see [2]). Based on our topology, the “box-in-box” growth pattern, occurring at the base of the total-group chondrichthyan, was replaced by polyodontode scales growing through appositional and areal growth in putative chondrichthyans [12, 29, 86, 87] and chondrichthyans [83, 88]. However, the polyodontode growth was already present prior to the origin of the total-group chondrichthyans since it is present in osteostracans [89, 90], in placoderms [91] and also in basal osteichthyans [2]. Furthermore, even in basal stem chondrichthyans, such as the Silurian ischnacanthiform *Nerepisacanthus denisoni*, “box-in-box” scales are present on the body and areal-growth polyodontode scales are present on the cheek region (S4 Table) [3]. Such polyodontode-type tesserae and *Nostolepis*-type scales are also found in the Early Devonian ischnacanthiform *Acritolepis ushakovi* [92]. Therefore, the “box-in-box” growth represents an evolutionary novelty at the base of the total-group chondrichthyans, but the presence of polyodontode scales (or the potential of forming polyodontode scales) remained present.

It is also likely that the so-called polyodontode condition regroups different and similar but non-homologous growth patterns. We agree with Qu et al. [2] who concluded that a complete revision of paleohistology of early vertebrates is needed.

Scale ontogeny is described from a single element, and then individual ontogeny is inferred. We showed that scale ontogeny reflects the individual ontogeny in *Triazeugacanthus*. For example, ganoine has not been observed in ground sections of juvenile *Triazeugacanthus*, whereas it was unambiguously present in adults. This ontogenetic difference suggests that ganoine develops later in ontogeny when mesodentine and bone are well developed. One could suggest that the ganoine layer could have been abraded during the fossilisation process, however, the outer surface of juvenile scales is covered with tissues interpreted as skin remnants (epidermis and mesenchyme) (Fig 2E). Thus, this difference reflects a true ontogenetic change rather than a taphonomic bias. The condition observed in *Triazeugacanthus* shows similarities with that found in the living *Polypterus* *senegalus*. In this basal actinopterygian, the scales extend first in surface area then in thickness and subsequently the ganoine layer is deposited only when the upper dentine layer is well developed in late juveniles [59, 67, 68]. Therefore, the late ontogenetic formation of ganoine in *Triazeugacanthus* is similar to what is described for the ganoine deposition in lepisosteid and polypterid scales [68, 93].

**Fig 7:**
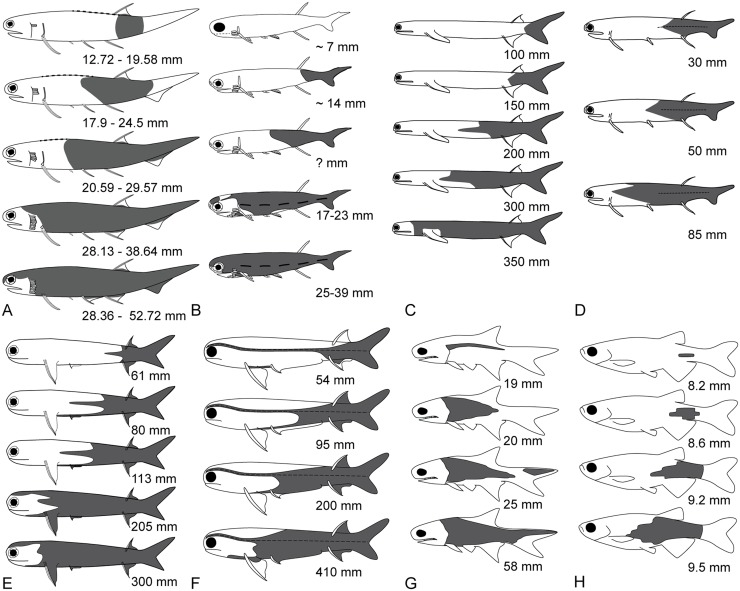
Development of the squamation pattern in various acanthodiformes (A-F) and actinopterygians (G-H). A: *Triazeugacanthus affinis*. B: *Lodeacanthus gaujicus* [modified from [18]]. C: *Acanthodes bronni* [modified from [20]]. D: *Acanthodes ovensi* [modified from [23]]. E: *Acanthodes gracilis* [modified from [21]. F: *Acanthodes bridgei* [modified from [8]]. G: *Elonichthys peltigerus* [modified from [1]]. H: *Danio rerio* [modified from [104]]. Estimated total length is given in A to G, whereas standard length is given in H.

Different scale shapes have been reported along the body (and head) of acanthodians [6, 7, 18, 37, 94–96]. One of these changes reported in various acanthodians (e.g. *Climatius*, *Ptomacanthus*, *Lodeacanthus*) is the presence of flat-based scales anteriorly and bulging-based scales posteriorly [18, 96]. In *Lodeacanthus*, Upeniece [18] described two types of scale bases in juveniles: fully-developed conical (or bulging) bases (type 1) and incompletely-developed flat bases with a deep ventral pit (type 2) from the ventral and dorso-lateral areas of the prepectoral region. Based on our findings on the bidirectional pattern of squamation from a relatively posterior origin in *Triazeugacanthus*, anterior scales would develop later in ontogeny than posterior scales, and thus would exhibit a younger phenotype. We suggest that type 2 (flat base) of *Lodeacanthus* corrresponds to younger scales (as in juvenile *Triazeugacanthus*), whereas type 1 (convex base) of *Lodeacanthus* corresponds to older scales (as in adult *Triazeugacanthus*) (Fig 7A and 7B). The allometry of the thickness/width ratio in *Triazeugacanthus* scales indicates that body scales grow first in area then in thickness (as already mentioned for *Polypterus*). This thickness change is reflected morphologically by the change in the shape of the basal layer from a flat surface in juveniles to a convex surface in adults. Retention of flat-based scales in the anterior part of body in adults (such as in *Ptomacanthus*) could suggest a heterochronic shift showing a juvenile feature in the last scales to develop. This presence of anterior flat-base scales has also been mentioned for scales attributed to chondrichthyans [96].

Growth zones are recognised in extant fish scales and are generally related to periodic changes of environmental conditions (seasonal or annual cycles) promoting the intake of nutritive elements [97–99]. Such variations, revealed by the alternation of short dark zones (rest zones) and large light zones (growth zones), occurred also during *Triazeugacanthus* life (Fig 3). Based on the growth of complete *Triazeugacanthus* specimens and that of isolated elements, we showed that the individual growth of scales is correlated to species ontogeny as suggested by Zidek [19] and Karatajute-Talimaa [88]; this is in contrast to Valiukevičius [32] who mentioned that the number of growth lamellae do not reflect the developmental stage of the animal. “box-in-box” growing scales are reliable proxies of species growth.

### Squamation pattern

In acanthodians, the squamation pattern has only been described in some acanthodiforms (S4 Table). Based on the literature, the general acanthodiform pattern of squamation is characterised by an initiation in the caudal region and an anterior progression following initially the lateral line trajectory [18, 19, 100] (Fig 7). Such pattern was described in various species [*i*.*e*. *Acanthodes bridgei* [8], *A*. *bronni* [20], *A*. *gracilis* [21], *A*. *ovensi* [23], *Lodeacanthus gaujicus* [18]] and is shared with some early actinopterygians [1, 101] and many extant teleost fish [102, 103] (Fig 7). This observed caudal-rostral progression differs from the hypothesized direction suggested by Hanke and Wilson [29]. In their phylogenetic analysis of acanthodians, Hanke and Wilson [29] coded for a character (their character 21) that takes into account the scale growth origin (or initiation). The 20 acanthodian taxa, with the exception of *Obtusacanthus* and *Lupopsyroides* (which they used as out-groups), were coded as having the first scales develop below the second dorsal fin (assuming that dorsal fin of the single-dorsal-fin taxa correspond to the second dorsal fin). They used the larger size of the scales as a proxy of the first formed scales.

In *Triazeugacanthus*, we found a pattern of squamation similar to that suggested by Hanke and Wilson [29] rather than that described from growth series in the literature. Based on the progression of the squamation from the size series and scale proportions, the squamation of *Triazeugacanthus* is initiated in the posterior region of the body, below the dorsal fin and progresses bidirectionally; the postero-anterior direction is predominant over the antero-posterior direction because of the relatively posterior position of the site of initiation. As a result the number of rows as well as the number of scales per row increases during the ontogeny of *Triazeugacanthus*. This information refutes the hypothesis suggested by Karatajute-Talimaa [88] that the number of scales remained stable during the ontogeny of acanthodians.

General conditions of squamation have already been described in different taxonomic groups. The squamation of the heterostracan *Dinaspidella elizabethae* developed first ventrally and dorsally following an antero-posterior direction [105]. Such an antero-posterior pattern is also described for the thelodonts *Lanarkia horrida* [106], *Loganellia scotica* [107] and *Thelodus laevis* [108]. There is only few information relative to the direction of squamation for placoderms [*i*.*e*., ontogeny of *Asterolepis ornata* [18]]. Although the information is sparse for jawless vertebrates and early gnathostomes (and not necessarily representative of the complete phylogenetic diversity), an antero-posterior patterning of body scales is suggested to be plesiomorphic for vertebrates.

The scarce information available in Palaeozoic chondrichthyans indicates that scales are present first along the lateral line [109–111]. In extant chondrichthyans, scale development is generally considered to differ from other gnathostomes because head and body scales do not form sequentially but rather simultaneously and in a non-regular pattern [104, 112–114]. However, Johanson et al. [113, 114] described an initiation of primary scale (or patterned tail scales) development on the extremity of the caudal fin progressing anteriorly along the caudal peduncle, then followed by a more irregular origin and arrangement of body scales, from anterior to posterior, to cover the ventral and dorsal lobes of the caudal fin; the initial sequential caudal scales are subsequently lost during ontogeny. Johanson et al. [113, 114] suggested that this regulated and sequential development of the caudal primary scales retained in early ontogeny may represent the plesiomorphic condition for chondrichthyans.

In the living basal actinopterygian *Polypterus senegalus*, there are two sites of squamation initiation which start almost simultaneously [115]: an anterior site located just behind the pectoral girdle, and a second site in the caudal region. In both sites, scales form close to the lateral line. Thus, in *Polypterus*, scales develop antero-posteriorly from the anterior site and bidirectionally from the caudal site. In the Carboniferous actinopterygian *Elonichthys peltigerus* [1, 116] and the Triassic *Brookvalia gracilis* [111], scales initiate in the anterior region of the body near the lateral line and progress posteriorly (Fig 7G). This antero-posterior patterning is also conserved in the living *Amia calva*, where the first scales form on the lateral line just behind the pectoral girdle then the squamation extends posteriorly along the lateral line [117, 118]. In the living *Lepisosteus oculatus* and *L*. *osseus*, the first scales appear along the lateral line in the tail region, then the squamation spreads anteriorly [119, 120]. Sire and Akimenko [104] reported that the main generalised condition of scale development in teleosts (Fig 7H) is the initiation of the first scales along the midline row at the level of the caudal peduncle, followed by a rapid progression of the squamation anteriorly and posteriorly along this row, while new rows are added ventrally and dorsally. Although further information relative to the squamation patterning in actinopterygians are needed, it seems that the plesiomorphic condition for the group is the antero-posterior direction, and that a postero-anterior direction (similar to the acanthodian pattern) would have occurred near the base of the neopterygians; however, as reported in *Polypterus*, both patterns are present in some species.

Independently of the direction of progression, Johanson et al. [113] considered that the presence of scale patterning maintained through ontogeny might be a synapomorphy of crown group gnathostomes. Our data on acanthodian scale patterning through ontogeny corroborates this hypothesis. The bidirectional pattern of squamation in juvenile *Triazeugacanthus* is similar to the pattern reported in most acanthodian [29] and teleost fish [102], while a unidirectional development seems to be restricted to some acanthodiforms and living chondrichthyans during early ontogeny solely. The difference between the pattern described for *Triazeugacanthus* and that reported for acanthodiforms might be biased by the greater number of specimens observed, the availability of younger developmental stages and the exceptional state of preservation allowing the fossilisation of soft and weakly mineralised tissues such as developing scales. The squamation pattern observed in acanthodians might well represent a precursor condition to that of chondrichthyans which would also corroborate the stem-group position of acanthodians.

Three hypotheses, mainly based on scale development in teleost fish, are given concerning the region of first scale development: (1) gene expression patterns, (2) lateral line induction, and (3) mechanical constraints imposed to the fish skin during swimming [102]. The first hypothesis suggests a role of *Shh* and/or *ScShh* which is known to be involved in the positional specification along the antero-posterior axis in vertebrates [114, 121]. *Shh* expression is involved in the control of epidermal-dermal interactions but seems not essential for scale initiation and patterning of squamation [104]. The second hypothesis proposes that the development of the lateral line neuromasts during the embryonic phase of fish ontogeny could induce the formation of the first scales notably because whatever the portion of the body from which the scale initiate (anterior or posterior), their development follows the lateral line in several actinopterygians [122–124]. However, Wada et al. [125] have shown that the final position of each terminal neuromast coincided with the position of a scale in the proximal region of the caudal fin in zebrafish. They suggested that the prospective scale region may emit chemoattractive factors that regulate neuromast migration. Thus, scale patterning would in part regulate lateral line patterning rather than the opposite. The third hypothesis is suggested by the flexibility of the body in the mobile posterior region, which has been proposed as a possible factor triggering early scale formation in this region [102, 103]; however, the anterior site of initiation would not be subject to special mechanical epigenetic constraint. In extant teleosts, opposite directions of squamation development are observed in closely related species such as in two cyprinids (posterior-anterior in *Danio rerio* *versus* anterior-posterior in *Cyprinus carpio*) and were related to swimming mode in juveniles prior to scale formation [102, 103]. Our data on *Triazeugacanthus* squamation do not allow us to choose among the three hypotheses. However, the progression seems to be following grossly the trajectory of the lateral line system and most likely the body shape of *Triazeugacanthus* would suggest some type of undulatory locomotion with greater amplitude of movement in the dorsal-caudal region. On the other hand, the conservatism of patterning among acanthodians as well as the relative conservatism in other groups would rather suggest the importance of fundamental developmental pattern under the control of gene expression.

## Conclusion

The fossilised ontogeny of the Late Devonian acanthodian *Triazeugacanthus* allowed us to describe a scale structure similar to the *Acanthodes*-type scale and to define a bidirectional pattern of squamation. We identified three tissues composing the scales (*i*.*e*. a basal layer of acellular bone, a middle layer of mesodentine and a superficial layer of ganoine). Ontogenetic data (thickness/width ratio, growth zone distances, and squamation pattern) allowed us to recognize two types of growth (*i*.*e*. the “box-in-box” growth of mesodentine and basal bone and the subsequent superimpositional growth of the well-mineralised ganoine layer). *Triazeugacanthus* scales show similarities with acanthodians (e.g. “box-in-box” growth), chondrichthyans (e.g. squamation pattern), and actinopterygians (e.g. ganoine), which phylogenetically are interpreted considering acanthodians as stem chondrichthyans. The usage of scales as proxies to study developmental patterns and processes in extinct groups [1, 2], such as acanthodians, opens the possibility to not only determine phylogenetic relationships but infer developmental novelties important during the evolutionary history of early vertebrates.

## Supporting information

S1 Table *Triazeugacanthus affinis* specimens used for either histology or SEM-EDS X-ray analyses. Squamation cover and extent (as percentage of total length) are measured from head to tail.
(PDF)

S2 Table Data for linear regressions and ANOVA analysis related to ontogenetic stages of *Triazeugacanthus affinis*. Thickness and width values obtained from transverse ground sections of scales are given in log_10_ and *μ*m, except for thickness/width where values were log_10_(x + 1)-transformed.
(PDF)

S3 Table Results from successive deletion of characters pertaining to the histology, morphology and growth of scales and two spine characters. Only the characters relevant to acanthodian taxa have been deleted. For each analysis the length of the trees, the number of trees, as well as the resulting phylogenetic status of the acanthodians and the identification of the taxa at the base of either the monophyletic acanthodians or the total-group chondrichthyans have been recorded.
(PDF)

S4 Table Comparison of scale composition in various early gnathostomes. A, anterior; GZ, growth zone; P, posterior.
(PDF)

S1 Appendix Phylogenetic analysis. List of characters, coding matrix and results.
(PDF)

S1 File Data matrix for phylogenetic analyses of gnathostomes. (NEX)

S1 Fig Scales of the Frasnian acanthodiform *Homalacanthus concinnus*, Escuminac Formation, Miguasha, Quebec, Canada. A-D: MHNM 03-2215. A: SEM of the body squamation showing the alignment. B: Details from two scales showing the superficial ridges. C: SEM observations of a transverse section. D: Transverse ground section under polarised light. b, acellular bone; m, mesodentine; en, well-mineralised layer; l.l, lateral line; ne, neck; ri, superficial ridge; Sh, Sharpey’s fibers. Scale bar = 1 mm in A, 200 *μ*m in B, 100 *μ*m in C, 50 *μ*m in D.
(TIF)

S2 Fig Scales of the Frasnian actinopterygian *Cheirolepis canadensis*, Escuminac Formation, Miguasha, Quebec, Canada. A: MHNM 05-53, SEM of the body squamation. B: MHNM 05-53, details showing the ridged scale surface and the broad base. C: MHNM 05-152, SEM of lepidotrichial segments showing the ornamentation. D: Transverse ground section of a scale under polarised light. cb, cellular bone; m, mesodentine; g, ganoine; le, lepidotrichium; ri, superficial ridge. Scale bar = 2 mm in A, 200 *μ*m in B, 500 *μ*m in C, 50 *μ*m in D.
(TIF)

S3 Fig Representative spectra of *Triazeugacanthus affinis* samples using EDX punctual microanalysis of MHNM 03-1497 scales. A: MHNM 03-1497, location of spectra for EDX analyses. Note that the oxygen peak is non-significant and depends essentially on the vacuum level in the chamber of the environmental SEM. Scale bar: A = 1 mm.
(TIF)

S4 Fig Distances between growth lines in the scales of four adult specimens of *Triazeugacanthus affinis*. Circles are for the ganoine layers (superimpositional growth), squares for mesodentine and bone layers (“box-in-box” growth). The “box-in-box” patttern of growth is more irregular than the superimpositional growth of the multi-layered ganoine. Growth zones are numbered by the two growth lines that delimit the zone. Each coloured line represents one scale.
(TIF)

S5 Fig *Triazeugacanthus affinis* median ridge scales. A: Early juvenile, MHNM 03-1252. Dorsal mid-line scales develop before trunk scales; the latter are only present in the posterior region (red rectangle). B: Adult, MHNM 03-1497. Dorsal scale showing the presence of a median ridge (white arrow). C: Late juvenile, MHNM 03-2684. Two parallel scale rows (white arrows) are present anterior to trunk scales (red rectangle). Scale bars: A = 2 mm, B, C = 1mm.
(TIF)

S6 Fig Trees generated in the phylogenetic analysis of selected early gnathostomes (79 taxa, 267 characters). A: Strict consensus of 10,000 most parsimonious trees (711 steps). B: Adams consensus of 10,000 most parsimonious trees (711 steps).
(JPG)

S7 Fig Trees generated in the phylogenetic analysis of selected early gnathostomes (79 taxa, 266 characters). Each analysis is realized with the exclusion of one scale-related character.
(JPG)

S8 Fig Trees generated in the phylogenetic analysis of selected early gnathostomes (79 taxa, 266 characters). Each analysis is realized with the exclusion of one scale-related character.
(JPG)

S9 Fig Trees generated in the phylogenetic analysis of selected early gnathostomes (79 taxa, 266 characters). Each analysis is realized with the exclusion of one scale-related character.
(JPG)

S10 Fig Trees generated in the phylogenetic analysis of selected early gnathostomes (79 taxa, 266 characters). Each analysis is realized with the exclusion of one scale-related character.
(JPG)

S11 Fig Trees generated in the phylogenetic analysis of selected early gnathostomes (79 taxa, 266 characters). Each analysis is realized with the exclusion of one scale-related character.
(JPG)

S12 Fig Tree generated in the phylogenetic analysis of selected early gnathostomes (79 taxa, 266 characters). The analysis is realized with the exclusion of one scale-related character.
(JPG)
