# Supplementary material for: From body scale ontogeny to species ontogeny: Histological and morphological assessment of the Late Devonian acanthodian Triazeugacanthus affinis from Miguasha, Canada
Source: PLoS One. 2017 Apr 12;12(4):e0174655. doi: 10.1371/journal.pone.0174655 (PMC5389634; doi:10.1371/journal.pone.0174655)
Supplement: S1 Table — Squamation cover and extent (as percentage of total length) are measured from head to tail. (PDF) [file pone.0174655.s001.pdf]

S1 Table. *Triazeugacanthus affinis* specimens used for either histology or SEM-EDS X-ray analyses.

Specimen ID	*Analyses*	*TL (mm)*	*Squamation extent (%)*
MHNM 03-1817	Histology	47.51	100
MHNM 03-1971	Histology	49.21	100
MHNM 03-78	Histology	26.52	100
MHNM 03-2620	Histology	35.44	100
MHNM 03-1497	EDS X-ray - SEM	45.29	100
MHNM 03-1819	Histology - SEM	23.9	48
MHNM 03-398	Histology - EDS X-ray - SEM	17.02	57
MHNM 03-701	Histology	33.18	49
MHNM 03-2684 2	Histology	12.71	NA
MHNM 03-529	Histology	43.28	NA
MHNM 03-372	Histology	16.02	13
MHNM 03-740	Histology	21.92	33
MHNM 03-1250	Histology	21.37	37
MHNM 03-210	Histology - SEM	17.55	33
MHNM 03-978	Histology	21.64	38
MHNM 03-2631	Histology - SEM	31.47	43
MHNM 03-259	Histology - SEM	13.82	55
MHNM 03-2570	Histology - SEM	22.96	63
MHNM 03-1460	SEM	NA	NA
