# Supplementary material for: From body scale ontogeny to species ontogeny: Histological and morphological assessment of the Late Devonian acanthodian Triazeugacanthus affinis from Miguasha, Canada
Source: PLoS One. 2017 Apr 12;12(4):e0174655. doi: 10.1371/journal.pone.0174655 (PMC5389634; doi:10.1371/journal.pone.0174655)
Supplement: S2 Table — Thickness and width values obtained from transverse ground sections of scales are given in log10 and μm, except for thickness/width where values were log10(x + 1)-transformed. (PDF) [file pone.0174655.s002.pdf]

Triazeugacanthus affinis **scale measurements used for linear regressions and ANOVA analysis related to ontogenetic stages.**

Specimen ID	*Thickness*	*Width*	*Thickness/Width*
**Adults**			
MHNM 03-1817	1.98515692	2.37391744	0.1487706
	2.08149133	2.25880723	0.22135931
	2.02710079	2.23081919	0.21100786
	1.98237979	2.27596288	0.17858778
	1.85448823	2.25825405	0.14447147
	1.90661514	2.33804392	0.13682039
	1.93785877	2.28935703	0.1599115
	2.02391472	2.31377867	0.17984526
	1.85838078	2.32332224	0.12801586
	1.96465084	2.32939991	0.15587279
	1.90661514	2.26083908	0.15907374
	1.84801043	2.38659527	0.11036896
	1.85692817	2.34220153	0.12291501
	1.87785491	2.33587494	0.12979338
	1.78843	2.42931454	0.08941781
	1.76285105	2.36087351	0.09772053
	1.84712209	2.32285992	0.12528501
	1.86486099	2.34399317	0.12443688
	1.76732658	2.34682223	0.10151765
	1.79919576	2.39244369	0.09868679
	1.75554711	2.40823318	0.08724594
	1.6531353	2.33231522	0.08254285
	1.89063895	2.41876517	0.11273801
	1.91450182	2.21415744	0.17654987

Specimen ID	*Thickness*	*Width*	*Thickness/Width*
	**1.93337085**	**2.28385731**	**0.16022341**
	**1.9006784**	**2.3290784**	**0.13764097**
	**1.89458188**	**2.34174703**	**0.13262369**
	**1.92292777**	**2.39874011**	**0.12526634**
	**1.96364146**	**2.28389571**	**0.16977726**
	**1.88910553**	**2.27324904**	**0.15011482**
	**1.89247862**	**2.35861813**	**0.12771031**
	**1.76732658**	**2.44797931**	**0.08228828**
	**1.84636186**	**2.38736313**	**0.10982785**
	**1.79342017**	**2.32698477**	**0.11150062**
	**1.82326551**	**2.30633494**	**0.12345931**
	**1.810864**	**2.42202979**	**0.09510406**
	**1.82030657**	**2.24539914**	**0.13854183**
	**1.79450199**	**2.35754228**	**0.10499946**
	**1.74697652**	**2.35296162**	**0.09612782**
	**1.83206161**	**2.38705864**	**0.10673946**
	**1.8297025**	**2.20988213**	**0.15127697**
	**1.71962955**	**2.29218516**	**0.10297342**
	**1.72662942**	**2.186801**	**0.12923857**
	**1.69838766**	**2.19892638**	**0.11920168**
	**1.75442479**	**2.13631779**	**0.15077372**
	**1.66238002**	**2.30477479**	**0.08913719**
	**1.72180237**	**2.29339571**	**0.10317673**
	**1.86987095**	**2.24166351**	**0.15376075**
	**1.87096543**	**2.25278994**	**0.15079381**
	**1.78947495**	**2.32767968**	**0.11045428**
	**1.75539458**	**2.4323968**	**0.08292058**

Specimen ID	*Thickness*	*Width*	*Thickness/Width*
	**1.83126172**	**2.33489988**	**0.11845975**
	**1.78540102**	**2.41187045**	**0.09213665**
	**1.74873056**	**2.40717328**	**0.0862039**
	**1.74076521**	**2.39493474**	**0.08697633**
	**1.82548454**	**2.33250309**	**0.11765514**
	**1.82351987**	**2.39997814**	**0.10215252**
	**1.84551352**	**2.32263271**	**0.12493925**
	**1.81421429**	**2.31561143**	**0.11899579**
	**1.78730458**	**2.35867708**	**0.10322344**
	**1.78049013**	**2.35454828**	**0.10265668**
	**1.82836984**	**2.28187159**	**0.13096513**
	**1.86742624**	**2.4111734**	**0.1092158**
	**1.74413645**	**2.30968764**	**0.10446143**
	**1.85367388**	**2.37286599**	**0.1147969**
	**1.84147835**	**2.40419035**	**0.10506999**
	**1.84358779**	**2.31115179**	**0.12734784**
	**1.87588853**	**2.35894322**	**0.12346296**
**MHNM 03-78**	**1.52896809**	**2.159955**	**0.09127672**
	**1.41247735**	**2.06465263**	**0.08733895**
	**1.37021692**	**1.99353763**	**0.09274032**
	**1.37845241**	**2.17629099**	**0.06418837**
	**1.43507954**	**2.1725212**	**0.07300134**
	**1.38084413**	**2.03668877**	**0.08667279**
	**1.58593425**	**2.04339372**	**0.12993826**
**Juveniles**			
**MHNM 03-259**	**1.08075106**	**1.80247123**	**0.07547135**
	**0.98066972**	**1.82560538**	**0.05801225**

Specimen ID	*Thickness*	*Width*	*Thickness/Width*
	**1.18953478**	**1.89971046**	**0.0773336**
	**1.19251814**	**1.82301293**	**0.09137004**
	**1.01154926**	**1.77589235**	**0.06894647**
	**1.06795629**	**1.84396604**	**0.06725337**
**MHNM 03-398**	**1.16346569**	**2.13291759**	**0.0442603**
	**1.79074948**	**2.2774052**	**0.12257467**
	**1.7816405**	**2.30105388**	**0.11474551**
	**1.81914236**	**2.34431759**	**0.11341447**
	**1.81931357**	**2.36654436**	**0.10844363**
	**1.80452106**	**2.16291672**	**0.15779856**
	**1.7706974**	**2.26426516**	**0.12088513**
	**1.78005075**	**2.27008229**	**0.12174697**
	**1.79025701**	**2.29747599**	**0.11760758**
	**1.67878234**	**2.37475384**	**0.07968244**
	**1.71594488**	**2.43429259**	**0.07601108**
	**1.75854857**	**2.19385616**	**0.13577559**
	**1.72914858**	**2.38246732**	**0.08713086**
	**1.79971264**	**2.3466209**	**0.10851491**
	**1.81603571**	**2.31550853**	**0.11945776**
	**1.81132696**	**2.14746917**	**0.16469972**
	**1.69334875**	**2.33663582**	**0.08897176**
	**1.82459438**	**2.31687575**	**0.12119803**
	**1.75472283**	**2.30942788**	**0.1068031**
	**1.71222024**	**2.31168183**	**0.09743094**
	**1.78712027**	**2.29399199**	**0.11768999**
	**1.74786218**	**2.27622736**	**0.11268339**
	**1.8012802**	**2.25870192**	**0.12994802**

Specimen ID	*Thickness*	*Width*	*Thickness/Width*
	**1.76062601**	**2.26810504**	**0.11754591**
	**1.84653504**	**2.28897319**	**0.13387262**
	**1.77603252**	**2.3407276**	**0.1046446**
	**1.77321578**	**2.31116027**	**0.11051274**
	**1.82957392**	**2.40689258**	**0.10197232**
	**1.76964314**	**2.39021598**	**0.09327004**
	**1.69155003**	**2.34559523**	**0.0869989**
	**1.64443859**	**2.2861195**	**0.08926973**
	**1.74845938**	**2.35895652**	**0.09523564**
	**1.79025701**	**2.32248393**	**0.11180387**
	**1.78611939**	**2.3572295**	**0.10327896**
	**1.74179744**	**2.26527312**	**0.11380567**
	**1.69563925**	**2.32839388**	**0.09094219**
	**1.67878234**	**2.30483292**	**0.09221674**
	**1.72253521**	**2.28283728**	**0.10558899**
	**1.78173384**	**2.19257582**	**0.1424807**
	**1.74766035**	**2.19293525**	**0.13312192**
	**1.80953278**	**2.23450969**	**0.13857342**
	**1.69790903**	**2.28844598**	**0.09923921**
	**1.76324076**	**2.34992218**	**0.1000296**
	**1.66376687**	**2.35372201**	**0.08069678**
	**1.68946869**	**2.36597255**	**0.08300724**
	**1.72118386**	**2.29400965**	**0.1029164**
	**1.68946869**	**2.34325888**	**0.0870452**
	**1.27036818**	**2.17037483**	**0.0514962**
	**1.24970686**	**2.14386506**	**0.05215406**
	**1.38300136**	**2.09971295**	**0.07627419**

Specimen ID	*Thickness*	*Width*	*Thickness/Width*
	**1.36240164**	**2.05037883**	**0.08103281**
	**1.16102729**	**2.01476847**	**0.05692091**
	**1.3510831**	**1.95397732**	**0.09674329**
	**1.22019248**	**1.86811954**	**0.08811596**
	**1.24367359**	**2.01156669**	**0.06842716**
	**1.19762174**	**1.94086226**	**0.07210916**
	**1.28764648**	**1.98637023**	**0.0792223**
	**1.19536891**	**2.02051645**	**0.06053646**
	**1.19611926**	**1.98205721**	**0.06584291**
**MHNM 03-529**	**1.35672862**	**2.22718892**	**0.05490149**
	**1.37341658**	**2.21878134**	**0.05795862**
	**1.35672862**	**2.22967831**	**0.05460662**
	**1.36461119**	**2.12762534**	**0.0691418**
	**1.27685162**	**2.13760379**	**0.05606576**
	**1.36864806**	**2.12964293**	**0.06943959**
	**1.53093605**	**2.23381551**	**0.07853211**
	**1.48452746**	**2.239636**	**0.07031441**
	**1.46093167**	**2.17352261**	**0.07694055**
	**1.48413842**	**2.31472983**	**0.059832**
	**1.463903**	**2.27603141**	**0.06225265**
	**1.70393435**	**2.32960887**	**0.09228871**
	**1.65620769**	**2.28604535**	**0.0914948**
	**1.6690414**	**2.21928354**	**0.10778002**
	**1.63007297**	**2.26798312**	**0.08997283**
	**1.71918433**	**2.1805098**	**0.12894187**
	**1.77674369**	**2.19760939**	**0.13970039**
	**1.4543844**	**2.213943**	**0.06965211**

Specimen ID	*Thickness*	*Width*	*Thickness/Width*
	**1.4203791**	**2.00199307**	**0.10107695**
	**1.57754784**	**2.28363952**	**0.07800238**
	**1.53493856**	**2.20018804**	**0.08498631**
	**1.57506991**	**2.17684394**	**0.09696722**
	**1.53832862**	**2.15701595**	**0.09363508**
	**1.56037779**	**2.13020341**	**0.10355113**
	**1.54157557**	**2.00750296**	**0.12776437**
	**1.48110138**	**2.2459543**	**0.06887166**
	**1.38747458**	**2.13831897**	**0.07095447**
	**1.36632565**	**2.1607415**	**0.06466024**
	**1.43837018**	**2.14021501**	**0.07870343**
	**1.34289245**	**2.15885064**	**0.06174316**
**MHNM 03-701**	**1.40835604**	**2.06530944**	**0.08647241**
	**1.56336858**	**2.07669637**	**0.11616613**
	**1.43513006**	**2.03840855**	**0.09666659**
	**1.51767792**	**2.14303448**	**0.0923496**
	**1.76081438**	**2.37354091**	**0.09479756**
	**1.78237228**	**2.25071512**	**0.12715003**
**MHNM 03-2620**	**1.75208688**	**2.25109288**	**0.11957007**
	**1.77359631**	**2.26749322**	**0.12080518**
	**1.74245017**	**2.26784261**	**0.11336456**
	**1.74684429**	**2.24804442**	**0.11904302**
	**1.82901415**	**2.29775176**	**0.12704987**
	**1.7631059**	**2.28420732**	**0.11435415**
	**1.72978014**	**2.32752445**	**0.09777659**
	**1.71962955**	**2.29374695**	**0.10264421**
	**1.65292289**	**2.35816907**	**0.07814149**

Specimen ID	*Thickness*	*Width*	*Thickness/Width*
	**1.65143309**	**2.29938743**	**0.08811095**
	**1.66238002**	**2.29045534**	**0.09183013**
	**1.76971695**	**2.24664627**	**0.12498673**
	**1.70137799**	**2.3701447**	**0.08436326**
	**1.73443176**	**2.25208567**	**0.11515468**
	**1.62772421**	**2.17090095**	**0.10934269**
	**1.78237228**	**2.13911052**	**0.15830416**
	**1.69958627**	**2.16577537**	**0.12769768**
	**1.7043393**	**2.12384511**	**0.14007487**
	**1.74684429**	**2.23396774**	**0.1224597**
	**1.69164721**	**2.16869779**	**0.12495641**
	**1.45552997**	**2.10094548**	**0.08857827**
	**1.41560304**	**1.98515209**	**0.10360982**
	**1.20086937**	**1.94930439**	**0.07131849**
	**1.51995576**	**2.03382763**	**0.1160385**
	**1.5395326**	**2.05426661**	**0.11583651**
	**1.55738758**	**2.06371916**	**0.11781827**
	**1.36944182**	**2.02349107**	**0.08699816**
	**1.50806872**	**2.06257302**	**0.10684688**
	**1.23450212**	**2.02896943**	**0.06465312**
	**1.54539915**	**1.93254043**	**0.14924088**
**MHNM 03-740**	**1.53874387**	**2.00598307**	**0.12743041**
	**1.50529559**	**1.97166848**	**0.12765087**
	**1.54407267**	**1.8015192**	**0.19111034**
	**1.57353117**	**1.90955919**	**0.16473576**
	**1.57353117**	**1.89409245**	**0.16967794**
	**1.34201338**	**1.89013305**	**0.10824737**

Specimen ID	*Thickness*	*Width*	*Thickness/Width*
	**1.32783252**	**1.88029762**	**0.10729244**
	**1.35962989**	**1.97985877**	**0.09333654**
	**1.33697638**	**2.00754025**	**0.08404651**
	**1.34243944**	**2.06650821**	**0.07509756**
	**1.34201338**	**2.10398681**	**0.06929513**
