# Supplementary material for: From body scale ontogeny to species ontogeny: Histological and morphological assessment of the Late Devonian acanthodian Triazeugacanthus affinis from Miguasha, Canada
Source: PLoS One. 2017 Apr 12;12(4):e0174655. doi: 10.1371/journal.pone.0174655 (PMC5389634; doi:10.1371/journal.pone.0174655)
Supplement: S3 Table — Only the characters relevant to acanthodian taxa have been deleted. For each analysis the length of the trees, the number of trees, as well as the resulting phylogenetic status of the acanthodians and the identification of the taxa at the base of either the monophyletic acanthodians or the total-group chondrichthyans have been recorded. (PDF) [file pone.0174655.s003.pdf]

S3 Table. Results from successive deletion of characters pertaining to the histology, morphology and growth of scales and two spine characters.

Character	Length of MPTs	Number of trees	Acanthodii	Basal
5 - Dentine types	702	3549	paraphyly	“climatiformes”
8 - Odontode	703	9600	paraphyly	[climatiids + putative chondrichthyans + chondrichthyans] + [acanthodids + ischnacanthids + diplacanthids]
9 - Box-in-box growth	706	33990	paraphyly	Diplacanthiformes
11 - Profile (neck)	704	24 596	paraphyly	[climatiids + putative chondrichthyans + chondrichthyans] + [acanthodids + ischnacanthids + diplacanthids]
12 - Bulging base	703	100000	monophyly	Ischnacanthiformes
13 - Flat base	703	85933	monophyly	Ischnacanthiformes
14 - Flank scale alignement	707	100000	monophyly	[Ischnacanthiformes + Diplacanthiformes]
15 - Sensory line canals	704	4388	paraphyly	[Ischnacanthiformes + Diplacanthiformes]
127 - Anal fin spine	713	100000	monophyly	putative chondrichthyans
128 - Paired pectoral fin spines	708	20631	monophyly	putative chondrichthyans
260 - Areal growth	709	2702	monophyly	Acanthodiformes
261 - Pore canal system	706	30978	paraphyly	[Ischnacanthiformes + Diplacanthiformes]
263 - Appositional growth	709	2622	monophyly	Acanthodiformes
264 - Hypermineralized superficial layer of scales	705	31251	paraphyly	[Ischnacanthiformes + Diplacanthiformes]
265 - Enameloid/Enamel	709	100000	monophyly	Ischnacanthiformes
266 - Mono/multi-layered enamel	709	1708	paraphyly	Diplacanthiformes
