# Supplementary material for: From body scale ontogeny to species ontogeny: Histological and morphological assessment of the Late Devonian acanthodian Triazeugacanthus affinis from Miguasha, Canada
Source: PLoS One. 2017 Apr 12;12(4):e0174655. doi: 10.1371/journal.pone.0174655 (PMC5389634; doi:10.1371/journal.pone.0174655)
Supplement: S4 Table — A, anterior; GZ, growth zone; P, posterior. (PDF) [file pone.0174655.s004.pdf]

S4 Table. Comparison of scale composition in various early vertebrates.

	Superficial layer			*Middle layer*				*Basal layer*			*Size of flank scales*	*Box-in-box growth*	*Superpositional growth*	*Polyodontod e growth*	*Squamation primordium*	*Squamation direction*
	Surface	*Micro relief*	*Enamel-like*	*Vascular canal*	*Pore canal*	*Dentine*	*Neck*	*Bone*	*Sharpey's fibers*	*Shape*						
**Diplacanthiformes**																
Diplacanthus (Denison 1979) Burrow et al 2016	ridges	N/A	absent	present	present	mesodentine	slightly cons-tricted	acellular	present	convex	3/mm	5-6 GZ	N/A	absent	?	?
Uraniacanthus (Newman *et al.* 2012)	3-5 grooves	absent	absent	present	present	dentine	present	acellular	present	flat	2-5/mm	2-4 GZ	absent	absent	?	?
Rhadinacanthus (Burrow et al 2016)	around 12	absent	absent	present	present	mesodentine	constricted	acellular	present	slightly slightly convex	1/mm	> 6-7 GZ	absent	absent	?	?
Milesacanthus (Burrow *et al.* 2009)	14-24 ridges	absent	absent	present	present	mesodentine	constricted	acellular	present	convex	1-2/2mm	12 GZ	absent	absent	?	?
Culmacanthus stewarti (Long 1983)	6-7 ridges	?	?	?	present	?	constricted	?	?	convex	?	?	?	?	?	?
Tetanopsyrus lindoei (Hanke et Wilson 2001)	smooth	?	no histology								6/mm	?	?	absent	?	?
Gladiobranchus probaton	6-9 ridges	absent	absent	present	absent	orthodentine	constricted	acellular	present	slightly	2/mm	6 GZ	absent	absent	?	?

(Hanke&Davis 2008)										convex						
**Climatiiformes**																
Nostolepis gaujensis (Gross 1971; Valiukevicius and Burrow 2005; Burrow *et al.* 2009)	4-6 ridges	15 µm diameter	birefrin-gent duro-dentine	present	present	mesodentine	absent	cellular	present	modera-tely convex	1/1.5 mm	> 6 GZ	present	absent	?	?
Cassidiceps vermiculatus (Gagnier and Wilson 1996)	smooth	absent					high and constricted			convex					?	?
Climatius reticulatus (Burrow 2015)	ridges	?	absent	present	absent	mesodentine	present	cellular	present	slightly convex	3-4/mm	absent	absent	areal	?	?
Parexus recurvus (Burrow et al 2013)	ridges	?	absent	?	?	?	absent	acellular		flat or slightly concave	1-2/mm	absent	absent	3-5 appositional GZ	?	?
Vernicomacanthus waynensis (Miles 1973)	ridges	?	no histology								2/mm	?	?	?	?	?
**Acanthodiformes**																
Triazeugacanthus affinis – juveniles	smooth	N/A	absent	absent	absent	mesodentine	poorly developed	N/A	absent	flat	12/mm	1-2 GZ	absent	absent	dorsal region	bidirectional
Triazeugacanthus affinis – adults	smooth	2.5 µm diameter	ganoine	absent	absent	mesodentine	poorly developed	acellular	present	convex	5/mm	> 11 GZ	present	absent	N/A	N/A

Lodeacanthus gaujicus (Upeniece 2011)	smooth	absent	present	absent	absent	mesodentine	constricted	acellular	?	convex	7/mm	?	absent	absent	caudal	P > A
Acanthodes sp. (Derycke and Chancogne-Weber 1995)	smooth	present	ganoine	absent	absent	mesodentine	?	acellular	?	?	?	?	?	?	?	?
Acanthodes bridgei (Zidek 1985)	smooth	absent	no histology									present	?	?	caudal	P > A
Acanthodes bronni (Heidtke 1990)	no histology														caudal	P > A
Acanthodes gracilis (Zajic 2005)	no histology										5/mm	?	?	?	caudal	P > A
Acanthodes lopatini (Beznosov 2009)	smooth	absent	ename-loid	absent	absent	dentine	developed	acellular	?	convex	?	present	absent	absent	caudal	P > A
Acanthodes ovensi (Dineley 1999)	no histology														caudal	P > A
Promesacanthus milesi (Hanke 2008)	smooth	?	?	?	present	dentine	present	acellular	present	tumid/ convex	4/mm	4-5 GZ	absent	absent	?	?
Melanoacanthus minutus (Cumbaa and Schultze 2002)	smooth	?	?	absent	absent	dentine	?	acellular	?	?	9/mm	present	absent	absent	?	?
Teneracanthus (Burrow and Young	smooth	?	present	absent	absent	mesodentine	present	acellular	?	convex	4-5/mm	present	absent	absent	?	?

2005)																
Mesacanthus mitchelli (Baron 2015) Watson 1937	smooth	?	?	?	?	orthodentine	?	?	?	?	16/mm	?	?	?	?	P > A
Homalacanthus concinnus<br><br>(Appendix S8)	ridges	absent	present	absent	absent	mesodentine	constricted	acellular	present	convex	4/mm	present	present	absent	dorsal	?
Cheiracanthus splendens<br><br>(Denison 1979, Trinajstic 2001)	ridges	?	?	present	absent	orthodentine	constricted	cellular	?	tumid/ convex	2-3/mm	present	absent	absent	?	?
Acanthodidae indet. (Burrow *et al.* 2009)	smooth	?	duro-dentine	present	absent	dentine	slightly cons-tricted	acellular	?	convex	2-3/mm	10 GZ	absent	absent	?	?
Halimacanthodes ahlbergi<br><br>(Burrow *et al.* 2012)	smooth	present	enameloid	absent	absent	dentine	shallow	acellular	present	convex	2-3/mm	?	?	?	?	?
**Ischnacanthiformes**																
Gomphonchus<br><br>(Gross 1971, Burrow 1999)	ridges				absent				present			present				
Poracanthodes (Gross 1971; Valiukevičius 1995, Burrow 1999)	ridges	?	absent	present	present	mesodentine and/or orthodentine	constricted	cellular and acellular	?	?	?	12 GZ	absent	absent	?	?

Radioporacanthodes scheii (Burrow 2013)	4-8 ridges	honey-comb	durodentine	present	present	mesodentine	constricted	cellular	?	convex	9-10/mm	14 GZ	absent	absent	?	?
Ischnacanthus gracilis (Watson 1937)	smooth	?	no histology								4/mm	?	?	?	?	?
Nerepisacanthus (Burrow and Rudkin 2014)	smooth	?	absent	absent	absent	mesodentine	absent	cellular	?	convex		absent	present	present	?	?
Acritolepis (Valiukevicius 2003)	2-6	absent	absent	present	absent	mesodentine	present	cellular	present	slightly convex	3-7/mm	absent	present	absent	?	?
**Acanthodian Indet.**																
Paucicanthus vanelsti (Hanke 2002)	smooth	no histology					low	no histology		flat	no histology				?	?
Machaeracanthus pectinatus *Burrow&Young 2005*	4-12 ridges	absent	absent	absent	absent	orthodentine + mesodentine	constricted	acellular	?	slightly convex	0.5-1/mm	present	present	absent	?	?
Machaeracanthus goujeti (Botella *et al.* 2012)	8-12 ridges	absent	absent	absent	absent	mesodentine	pronounced	cellular	present	slightly convex	0.5-1/mm	present	absent	absent	?	?
Lupopsyrus pygmaeus (Hanke and Davis 2012)	smooth edges	absent	absent	absent	absent	mesodentine	present	no basal tissue	absent	flat	?	absent	absent	absent	2nd dorsal fin	?

Pechoralepis (acritolepidae) (Burrow *et al.* 2009)	ridges	absent	absent	present	absent	mesodentine	short or absent	cellular	?	convex	1-2/mm	absent	4-7	absent	?	?
Brochoadmones milesi (Hanke et Wilson 2006)	rings	?	absent	?	absent	mesodentine	low	cellular	present	Flat or slightly convex	3-4/mm	present	absent	absent	?	?
Obtusacanthus corroconis (Hanke et Wilson 2004)	ridges	?	absent	present	absent	orthodentine	absent	?	?	flat	2/mm	?	?	?	?	?
Kathemacanthus rosulentus (Hanke et Wilson 2010)	ridges	?	no histology								2-3/mm	absent	absent	appositional	?	?
Euthacanthus gracilis (Newman et al 2012)	> 4 ridges		no histology							slightly convex	2-3/mm	?	?	?	?	?
Ptomacanthus anglicus (Brazeau 2012)	ridges	?	?	present	absent	mesodentine	present	cellular	absent	convex	1-2/mm	absent	present	areal	?	?
**Early osteichthyans**																
Cheirolepis canadensis (Appendix S9)	ridges	present	ganoine	present	absent	mesodentine	short	cellular	?	convex	2/mm	>8	absent	absent	?	?

Psarolepis romeri (Qu *et al.* 2013)	smooth with pores	10-50 µm pore diameter	cosmine	present	present	orthodentine	present	cellular	present	convex	?	absent	present	present	?	?
Elonichthys peltigerus (Schultze and Bardack 1987)	no histology														lateral line – abdominal	A > P then, P > A
Eurynotus crenatus (Schultze 2015)	smooth	present	ganoine	present	present	dentine	absent	cellular	?	flat	?	?	present	absent	?	?
**Early chondrichthyan growing scales** (Karatajute-Talimaa 1992)																
Ctenacanthus	ridges	absent	present	?	?	dentine	?	?	?	concave	?	absent	absent	present	scales of various ages with different # of odontodes	?
Altholepis	ridges	absent	present	?	?	dentine				concave		absent	absent	present	?	?
Seretolepis	ridges	absent	present			dentine				concave		absent	absent	present	increasing # of scales during life	?
Protacrodus	ridges	absent	present			dentine				convex		absent	absent	present	?	?

BIBLIOGRAPHIE

Baron, M. G., 2015. An investigation of the genus *Mesacanthus* (Chordata : Acanthodii) from the Orcadian Basin and Midland Valley areas of northern and central Scotland using traditional morphometrics. PeerJ 3, e1331.

Beznosov, P., 2009. A redescription of the Early Carboniferous acanthodian *Acanthodes lopatini* Rohon, 1889. Acta Zoologica 90, 183–193.

Botella, H., Martinez-Perez, C., Soler-Gijon, R., 2012. *Machaerancathus gaujeti* n. sp. (Acanthodii) from the Lower Devonian of Spain and northwest France, with special reference to spine histology. Geodiversitas 34 (4), 761–783.

Brazeau, M. D., 2012. A revision of the anatomy of the Early Devonian jawed vertebrate *Ptomacanthus anglicus* Miles. Palaeontology 55 (2), 355–367.

Burrow, C. J., Davidson, R. G., Den Blaauwen, J. L., Newman, M. J., 2015. Revision of *Climatius reticulatus* Agassiz, 1844 (Acanthodii, Climatiidae), from the Lower Devonian of Scotland, based on new histological and morphological data. Journal of Vertebrate Paleontology, e913421.

Burrow, C. J., Den Blaauwen, J. L., Newman, M., Davidson, R. G., 2016. The diplacanthid fishes (Acanthodii, Diplacanthiformes, Diplacanthidae) from the Middle Devonian of Scotland. Palaeontologica Electronica 19 (1.10A), 1–83.

Burrow, C. J., Long, J. A., Trinajstic, K., 2009. Disarticulated acanthodian and chondrichthyan remains from the upper Middle Devonian Aztec Siltstone, southern Victoria Land, Antarctica. Antarctic Science 21 (01), 71–88.

Burrow, C. J., Newman, M. J., Davidson, R. G., Den Blaauwen, J. L., 2013. Redescription of *Parexus recurvus*, an Early Devonian acanthodian from the Midland Valley of Scotland. Alcheringa 37, 1–23.

Burrow, C. J., Rudkin, D., 2014. Oldest near-complete acanthodian : The first vertebrate from the Silurian Bertie Formation Konservat-Lagerstätte, Ontario. PLoS ONE 9 (8), e104171.

Burrow, C. J., Sues, H., 2013. Reassessment of *Ischnacanthus ? scheii* Spjeldnaes (Acanthodii, Ischnacanthiformes) from the latest Silurian or earliest Devonian of Ellesmere Island, arctic Canada1. Canadian Journal of Earth Sciences 50 (9), 945–954.

Burrow, C. J., Trinajstic, K., Long, J. A., 2012. First acanthodian from the Upper Devonian (Frasnian) Gogo Formation, Western Australia. Historical Biology : An International Journal of Paleobiology.

Burrow, C. J., Young, G. C., 1999. An articulated teleostome fish from the Late Silurian (Ludlow) of Victoria, Australia. Records of the Western Australian Museum 57, 1–14.

Burrow, C. J., Young, G. C., 2005. The acanthodian fauna of the Craven Peaks beds (Early to Middle Devonian), western Queensland. Queensland Museum. Memoirs 51 (1), 3–25.

Cumbaa, S. L., Schultze, H.-P., 2002. An Early Devonian (Emsian) acanthodian from the Bear Rock Formation, Anderson River, Northwest Territories, Canada. Canadian Journal of Earth Sciences 39 (10), 1457–1465.

Denison, R., 1979. Acanthodii. Vol. 5. Gustav Fischer, New York.

Derycke, C., Chancogne-Weber, C., 1995. Histological discovery on acanthodian scales from the Famennian of Belgium. Geobios 28, Supple (0), 31–34.

Dineley, D. L., 1999. Mid- and late Devonian fossil fishes sites of England and Wales. In : Dineley, D. L., Metcalf, S. J. (Eds.), Fossil fishes of Great britain. Joint Nature Conservation Comittee, United Kingdom, pp. 225–226.

Gagnier, P.-Y., Wilson, M. V. H., 1996. Early Devonian acanthodians from northern Canada. Palaeontology 39 (2), 241–258.

Gross, W., 1971. Downtonische und dittonische Acanthodier-reste des Ostseegebietes. Palaeontographica Abteilung A Palaeozoologie-Stratigraphie 136, 1–82.

Hanke, G. F., 2002. *Paucicanthus vanelsti* gen. et sp. nov., an Early Devonian (Lochkovian) acanthodian that lacks paired fin-spines. Canadian Journal of Earth Sciences 39 (7), 1071–1083.

Hanke, G. F., 2008. *Promesacanthus eppleri* n. gen., n. sp., a mesacanthid (Acanthodii, Acanthodiformes) from the Lower Devonian of northern Canada. Geodiversitas 30 (2), 287–302.

Hanke, G. F., Davis, S. P., 2012. A re-examination of *Lupopsyrus pygmaeus* Bernacsek & Dineley, 1977 (Pisces, Acanthodii). Geodiversitas 34 (3), 469–487.

Hanke, G. F., Davis, S. P., Wilson, M. V. H., 2001. New species of the acanthodian genus *Tetanopsyrus* from Northern Canada, and comments on related taxa. Journal of Vertebrate Paleontology 21 (4), 740–753.

Hanke, G. F., Wilson, M. V. H., 2004. New teleostome fishes and acanthodian systematics. In : Arratia, G., Wilson, M. V. H., Cloutier, R. (Eds.), Recent Advances in the Origin and Early Radiation of Vertebrates. Verlag Dr Friedrich Pfeil, München, pp. 189–216.

Hanke, G. F., Wilson, M. V. H., 2006. Anatomy of the Early Devonian acanthodian *Brochoadmones milesi* based on nearly complete body fossils, with comments on the evolution and development of paired fins. Journal of Vertebrate Paleontology 26 (3), 526–537.

Hanke, G. F., Wilson, M. V. H., 2010. The putative stem-group chondrichthyans *Kathemacanthus* and *Seretolepis* from the Lower Devonian MOTH locality, Mackenzie Mountains, Canada. In : Elliott, D. K., Maisey, J. G., Yu, X., Miao, D. (Eds.), Morphology, Phylogeny and Paleobiogeography of Fossil Fishes. Dr Friedrich Pfeil, München, pp. 159–182.

Heidtke, U. H. J., 1990. Studien über *Acanthodes* (Pisces : Acanthodii) aus dem saarpfälzischen Rotliengend (?Ober-Karbon - Unter-Perm - SW-Deutschland). Pollichia 19, 1–86.

Karatajute-Talimaa, V. N., 1992. The early stage of the dermal skeleton formation in chondrichthyans. In : Mark-Kurik, E. (Ed.), Fossil Fishes as Living Animals. Academy of Sciences of Estonia, Tallinn, pp. 223–231.

Long, J. A., 1983. A new diplacanthoid acanthodian from the Late Devonian of Victoria. Memoirs of the Association of Australasian Palaeontologists 1, 51–65.

Miles, R. S., 1973. Articulated acanthodian fishes from the Old Red Sandstone of England, with a review of the structure and evolution of the acanthodian shoulder-girdle. Bulletin of the British Museum of Natural History Geology 24 (2), 114–213.

Newman, M. J., Davidson, R. G., Blaauwen, J. L. D., Burrow, C. J., 2012. The Early Devonian acanthodian *Uraniacanthus curtus* (Powrie, 1870) n. comb. from the Midland Valley of Scotland. Geodiversitas 34 (4), 739–759.

Qu, Q., Zhu, M., Wang, W., 2013. Scales and dermal skeletal histology of an early bony fish *Psarolepis romeri* and their bearing on the evolution of rhombic scales and hard tissues. PLoS ONE 8 (4), e61485.

Schultze, H.-P., 2015. Scales, enamel, cosmine, ganoine, and early osteichthyans. Comptes Rendus Palevol (0).

Schultze, H.-P., Bardack, D., 1987. Diversity and size changes in palaeonisciform fishes (Actinopterygii, Pisces) from the Pennsylvanian Mazon Creek fauna, Illinois, U.S.A. Journal of Vertebrate Paleontology 7 (1), 1–23.

Trinajstic, K., 2001. Acanthodian microremains from the Frasnian Gneudna Formation, Western Australia. RECORDS-WESTERN AUSTRALIAN MUSEUM 20 (2), 187–198.

Upeniece, I., 2011. Palaeoecology and juvenile individuals of the Devonian placoderm and acanthodian fishes from Lode site, Latvia. Ph.D. thesis, University of Latvia.

Valiukevičius, J., 1995. Acanthodian histology : Some significant aspects in taxonomical and phylogenetical research. Geobios 28, Supple (0), 157–159.

Valiukevičius, J., 2003. Devonian acanthodians from Severnaya Zemlya Archipelago (Russia). Geodiversitas 25 (1), 131–204.

Valiukevičius, J., Burrow, C., 2005. Diversity of tissues in acanthodians with *Nostolepis*-type histological structure. Acta Palaeontologica Polonica 50 (3), 635–649.

Watson, D. M. S., 1937. The acanthodian fishes. Philosophical Transactions of the Royal Society of London. Series B, Biological Sciences 228 (549), 49–146.

Zajic, J., 2005. Permian acanthodians of the Czech Republic. Czech Geological Survey Special Papers 18, 6–42.

Zidek, J., 1985. Growth in *Acanthodes* (Acanthodii : Pisces) data and implications. Paläontologische Zeitschrift 59 (1), 147–166.

Zylberberg, L., Meunier, F. J., Laurin, M., 2016. A microanatomical and histological study of the postcranial dermal skeleton of the devonian actinopterygian *Cheirolepis canadensis*. Acta Palaeontologica Polonica 61 (2), 363–376.

BIBLIOGRAPHIE

Baron, M. G., 2015. An investigation of the genus *Mesacanthus* (Chordata : Acanthodii) from the Orcadian Basin and Midland Valley areas of northern and central Scotland using traditional morphometrics. PeerJ 3, e1331.

Beznosov, P., 2009. A redescription of the Early Carboniferous acanthodian *Acanthodes lopatini* Rohon, 1889. Acta Zoologica 90, 183–193.

Botella, H., Martinez-Perez, C., Soler-Gijon, R., 2012. *Machaerancathus gaujeti* n. sp. (Acanthodii) from the Lower Devonian of Spain and northwest France, with special reference to spine histology. Geodiversitas 34 (4), 761–783.

Brazeau, M. D., 2012. A revision of the anatomy of the Early Devonian jawed vertebrate *Ptomacanthus anglicus* Miles. Palaeontology 55 (2), 355–367.

Burrow, C. J., Davidson, R. G., Den Blaauwen, J. L., Newman, M. J., 2015. Revision of *Climatius reticulatus* Agassiz, 1844 (Acanthodii, Climatiidae), from the Lower Devonian of Scotland, based on new histological and morphological data. Journal of Vertebrate Paleontology, e913421.

Burrow, C. J., Den Blaauwen, J. L., Newman, M., Davidson, R. G., 2016. The diplacanthid fishes (Acanthodii, Diplacanthiformes, Diplacanthidae) from the Middle Devonian of Scotland. Palaeontologica Electronica 19 (1.10A), 1–83.

Burrow, C. J., Long, J. A., Trinajstic, K., 2009. Disarticulated acanthodian and chondrichthyan remains from the upper Middle Devonian Aztec Siltstone, southern Victoria Land, Antarctica. Antarctic Science 21 (01), 71–88.

Burrow, C. J., Newman, M. J., Davidson, R. G., Den Blaauwen, J. L., 2013. Redescription of *Parexus recurvus*, an Early Devonian acanthodian from the Midland Valley of Scotland. Alcheringa 37, 1–23.

Burrow, C. J., Rudkin, D., 2014. Oldest near-complete acanthodian : The first vertebrate from the Silurian Bertie Formation Konservat-Lagerstätte, Ontario. PLoS ONE 9 (8), e104171.

Burrow, C. J., Sues, H., 2013. Reassessment of *Ischnacanthus ? scheii* Spjeldnaes (Acanthodii, Ischnacanthiformes) from the latest Silurian or earliest Devonian of Ellesmere Island, arctic Canada1. Canadian Journal of Earth Sciences 50 (9), 945–954.

Burrow, C. J., Trinajstic, K., Long, J. A., 2012. First acanthodian from the Upper Devonian (Frasnian) Gogo Formation, Western Australia. Historical Biology : An International Journal of Paleobiology.

Burrow, C. J., Young, G. C., 1999. An articulated teleostome fish from the Late Silurian (Ludlow) of Victoria, Australia. Records of the Western Australian Museum 57, 1–14.

Burrow, C. J., Young, G. C., 2005. The acanthodian fauna of the Craven Peaks beds (Early to Middle Devonian), western Queensland. Queensland Museum. Memoirs 51 (1), 3–25.

Cumbaa, S. L., Schultze, H.-P., 2002. An Early Devonian (Emsian) acanthodian from the Bear Rock Formation, Anderson River, Northwest Territories, Canada. Canadian Journal of Earth Sciences 39 (10), 1457–1465.

Denison, R., 1979. Acanthodii. Vol. 5. Gustav Fischer, New York.

Derycke, C., Chancogne-Weber, C., 1995. Histological discovery on acanthodian scales from the Famennian of Belgium. Geobios 28, Supple (0), 31–34.

Dineley, D. L., 1999. Mid- and late Devonian fossil fishes sites of England and Wales. In : Dineley, D. L., Metcalf, S. J. (Eds.), Fossil fishes of Great britain. Joint Nature Conservation Comittee, United Kingdom, pp. 225–226.

Gagnier, P.-Y., Wilson, M. V. H., 1996. Early Devonian acanthodians from northern Canada. Palaeontology 39 (2), 241–258.

Gross, W., 1971. Downtonische und dittonische Acanthodier-reste des Ostseegebietes. Palaeontographica Abteilung A Palaeozoologie-Stratigraphie 136, 1–82.

Hanke, G. F., 2002. *Paucicanthus vanelsti* gen. et sp. nov., an Early Devonian (Lochkovian) acanthodian that lacks paired fin-spines. Canadian Journal of Earth Sciences 39 (7), 1071–1083.

Hanke, G. F., 2008. *Promesacanthus eppleri* n. gen., n. sp., a mesacanthid (Acanthodii, Acanthodiformes) from the Lower Devonian of northern Canada. Geodiversitas 30 (2), 287–302.

Hanke, G. F., Davis, S. P., 2012. A re-examination of *Lupopsyrus pygmaeus* Bernacsek & Dineley, 1977 (Pisces, Acanthodii). Geodiversitas 34 (3), 469–487.

Hanke, G. F., Davis, S. P., Wilson, M. V. H., 2001. New species of the acanthodian genus *Tetanopsyrus* from Northern Canada, and comments on related taxa. Journal of Vertebrate Paleontology 21 (4), 740–753.

Hanke, G. F., Wilson, M. V. H., 2004. New teleostome fishes and acanthodian systematics. In : Arratia, G., Wilson, M. V. H., Cloutier, R. (Eds.), Recent Advances in the Origin and Early Radiation of Vertebrates. Verlag Dr Friedrich Pfeil, München, pp. 189–216.

Hanke, G. F., Wilson, M. V. H., 2006. Anatomy of the Early Devonian acanthodian *Brochoadmones milesi* based on nearly complete body fossils, with comments on the evolution and development of paired fins. Journal of Vertebrate Paleontology 26 (3), 526–537.

Hanke, G. F., Wilson, M. V. H., 2010. The putative stem-group chondrichthyans *Kathemacanthus* and *Seretolepis* from the Lower Devonian MOTH locality, Mackenzie Mountains, Canada. In : Elliott, D. K., Maisey, J. G., Yu, X., Miao, D. (Eds.), Morphology, Phylogeny and Paleobiogeography of Fossil Fishes. Dr Friedrich Pfeil, München, pp. 159–182.

Heidtke, U. H. J., 1990. Studien über *Acanthodes* (Pisces : Acanthodii) aus dem saarpfälzischen Rotliengend (?Ober-Karbon - Unter-Perm - SW-Deutschland). Pollichia 19, 1–86.

Karatajute-Talimaa, V. N., 1992. The early stage of the dermal skeleton formation in chondrichthyans. In : Mark-Kurik, E. (Ed.), Fossil Fishes as Living Animals. Academy of Sciences of Estonia, Tallinn, pp. 223–231.

Long, J. A., 1983. A new diplacanthoid acanthodian from the Late Devonian of Victoria. Memoirs of the Association of Australasian Palaeontologists 1, 51–65.

Miles, R. S., 1973. Articulated acanthodian fishes from the Old Red Sandstone of England, with a review of the structure and evolution of the acanthodian shoulder-girdle. Bulletin of the British Museum of Natural History Geology 24 (2), 114–213.

Newman, M. J., Davidson, R. G., Blaauwen, J. L. D., Burrow, C. J., 2012. The Early Devonian acanthodian *Uraniacanthus curtus* (Powrie, 1870) n. comb. from the Midland Valley of Scotland. Geodiversitas 34 (4), 739–759.

Qu, Q., Zhu, M., Wang, W., 2013. Scales and dermal skeletal histology of an early bony fish *Psarolepis romeri* and their bearing on the evolution of rhombic scales and hard tissues. PLoS ONE 8 (4), e61485.

Schultze, H.-P., 2015. Scales, enamel, cosmine, ganoine, and early osteichthyans. Comptes Rendus Palevol (0).

Schultze, H.-P., Bardack, D., 1987. Diversity and size changes in palaeonisciform fishes (Actinopterygii, Pisces) from the Pennsylvanian Mazon Creek fauna, Illinois, U.S.A. Journal of Vertebrate Paleontology 7 (1), 1–23.

Trinajstic, K., 2001. Acanthodian microremains from the Frasnian Gneudna Formation, Western Australia. RECORDS-WESTERN AUSTRALIAN MUSEUM 20 (2), 187–198.

Upeniece, I., 2011. Palaeoecology and juvenile individuals of the Devonian placoderm and acanthodian fishes from Lode site, Latvia. Ph.D. thesis, University of Latvia.

Valiukevičius, J., 1995. Acanthodian histology : Some significant aspects in taxonomical and phylogenetical research. Geobios 28, Supple (0), 157–159.

Valiukevičius, J., 2003. Devonian acanthodians from Severnaya Zemlya Archipelago (Russia). Geodiversitas 25 (1), 131–204.

Valiukevičius, J., Burrow, C., 2005. Diversity of tissues in acanthodians with *Nostolepis*-type histological structure. Acta Palaeontologica Polonica 50 (3), 635–649.

Watson, D. M. S., 1937. The acanthodian fishes. Philosophical Transactions of the Royal Society of London. Series B, Biological Sciences 228 (549), 49–146.

Zajic, J., 2005. Permian acanthodians of the Czech Republic. Czech Geological Survey Special Papers 18, 6–42.

Zidek, J., 1985. Growth in *Acanthodes* (Acanthodii : Pisces) data and implications. Paläontologische Zeitschrift 59 (1), 147–166.

Zylberberg, L., Meunier, F. J., Laurin, M., 2016. A microanatomical and histological study of the postcranial dermal skeleton of the devonian actinopterygian *Cheirolepis canadensis*. Acta Palaeontologica Polonica 61 (2), 363–376.
