# Supplementary material for: From body scale ontogeny to species ontogeny: Histological and morphological assessment of the Late Devonian acanthodian Triazeugacanthus affinis from Miguasha, Canada
Source: PLoS One. 2017 Apr 12;12(4):e0174655. doi: 10.1371/journal.pone.0174655 (PMC5389634; doi:10.1371/journal.pone.0174655)
Supplement: S1 Appendix — List of characters, coding matrix and results. (PDF) [file pone.0174655.s005.pdf]

S1 Appendix. Phylogenetic analysis of early gnathostomes. List of characters, coding matrix and results.

List of characters (modified from [1]):

Modifications were made on 36 characters from the original data matrix from [1]: 16 characters have been rephrased (characters 9, 18, 19, 26, 31, 51, 81, 160, 177, 182, 190, 191, 241, 242, 257, 258), seven characters have been redefined (characters 7, 8, 149, 167, 195, 246, 260), 12 characters have been re-polarized (characters 7, 8, 11, 13, 104, 149, 167, 195, 196, 209, 246, 252), uninformative character 261 has been removed and six characters have been added (characters 262-267).

1. Tesselate prismatic calcified cartilage: absent (0); present (1).
In contrast to [1], but in agreement with [2], *Doliodus* has been re-coded as (1).

2. Perichondral bone: present (0); absent (1).
Perichondral bone is present in osteostracans ([3, 4, 5]; contra [2]). Character 2 is coded as (0) for the Osteostraci. Since the presence of perichondral bone is ambiguous in galeaspids [see [6] versus Zhu et Janvier (1998)], the Galeaspida is coded as (?). *Miguashaia* is coded as (0). *Diplacanthus* and *Rhadinacanthus* have been coded as (0) because perichondral ossification has been described in their scapulocoracoid [1].

3. Extensive endochondral ossification: absent (0); present (1).
Because of the morphological incompleteness *Diabolepis*, *Styloichthys*, *Youngolepis*, *Psarolepis* and *Meemannia* have to be coded as (?) rather than (1). *Miguashaia* and *Cheirolepis* are coded as (1).

4. Dentine: absent (0); present (1).
Dentine is absent in the Galeaspida [6] but present in the Osteostraci [5]. *Homalacanthus* and *Triazeugacanthus* are coded as (1).

5. Type of dentine: mesodentine (0); semidentine (1); orthodentine (2).
Homalacanthus and *Triazeugacanthus* are coded as (0). *Poracanthodes* has been coded (0&2) [7, 8].

6. Cosmine: absent (0); present (1).

7. Lepidotrichia: absent (0); present (1).
Character 7 is modified to solely represent the presence or absent of lepidotrichia. Character 262 takes into account the presence or absence of lepidotrichia-like scale alignment. The polarity has been changed to reflect the absence of lepidotrichia in both the Galeaspida and the Osteostraci. *Doliodus* is coded as (0).

8. Body scale growth pattern: polyodontode (0); monodontode (1).
The large rectangular body scales of osteostracans are considered to be polyodontodes because they are composed of multiple units [9]. Polyodontode composition has been described histologically in the Silurian osteostracan *Tremataspis* and *Oeselaspis* [5]. We refer to the information of the article 3 for our recognition of these two types. Thus, polyodontode is considered as the plesiomorphic condition. *Psarolepis* has been coded as (0) [10].

9. Body scale growth concentric "onion skin" pattern: absent (0); present (1).
Character 9 has been modified following [11, character 9]. *Bothriolepis* has been coded as (?). *Cheirolepis*, *Homalacanthus* and *Triazeugacanthus* have been coded as (1). *Miguashaia* has been coded as (0).

10. Body scales with peg-and-socket articulation: absent (0); present (1).
Specimens of *Botrhiolepis* ([12], R.C., pers. observ.) with scales lack peg-and-socket articulation; *Bothriolepis* has been coded as (0). *Cheirolepis* has been coded as (0).

11. Body scale profile: flattened (0); distinct crown and base demarcated by a constriction (neck) (1).
Polarity of character 11 has been changed because there is no constriction neck in osteostracan body scales [13]. *Bothriolepis* has been coded as (0). *Promesacanthus* has been recoded as (1) [14].

12. Body scales with bulging base: absent (0); present (1).

13. Body scales with flattened base: present (0); absent (1).
Polarity of character 13 has been changed because of the presence of flattened base in the scales of galeaspids [6].

14. Flank scale alignment: vertical rows (0); oblique rows or hexagonal/rhombic packing (1); disorganised (2).

15. Sensory line canal: passes between or beneath scales (0); passes over scales and/or is partially enclosed or surrounded by scales (1); perforates and passes through scales (2).

16. Sensory line network: preserved as open grooves (sulci) in dermal bones (0); sensory lines pass through canals enclosed within dermal bones (1).

17. Jugal portion of infraorbital canal joins supramaxillary canal: present (0); absent (1).

18. Dermal skull roof: includes large dermal plates (0); consists of undifferentiated plates or small polygonal plates (1).
Tessera *sensu* [11] are defined as flat-based, plate-like head coverings that are differentiated from the body scales, but do not form as distinct pattern as the dermal skull roof of placoderms or osteichthyans. These are different from the endoskeletal mineralization of chondrichthyans [15]. Because of this difference we rephrased the apomorphic character states.

19. Dermal skeleton morphology of the head: large interlocking polygonal plates (0); microsquamose, not larger than body squamation (1).
Character 19 has been rephrased because of the non-homologous conditions of dermal "tessera" sensu [11] and the tessera of chondrichthyans.

20. Extent of dermatocranial cover: complete (0); incomplete (scale-free cheek and elsewhere) (1).

21. Endolymphatic ducts open in dermal skull roof: present (0); absent (1).

22. Endolymphatic ducts with oblique course through dermal skull bones: absent (0); present (1).

23. Series of paired median skull roofing bones that meet at the dorsal midline of the skull (rectilinear skull roof pattern): absent (0); present (1).

Homalacanthus has been coded as (0) [[16]; pers. observ.]. Acanthodians for which the head scale condition is known have been coded accordingly (0).

24. Consolidated cheek plates: absent (0); present (1).
Homalacanthus has been coded as (0) [[16]; pers. observ.].

25. Pineal opening perforation in dermal skull roof: present (0); absent (1).
Cheirolepis has been coded as (0&1); *C. trailli* has an open pineal opening, whereas *C. canadensis* and *C. schultzei* lack a pineal opening [17, 18].

26. Enlarged postorbital dermal plate separate from orbital series, over the otic region: absent (0); present (1).
Character 26 has been rephrased.

27. Bony hyoidean gill-cover series (branchiostegals): absent (0); present (1).
[19] interpreted a highly modified small element in *Onychodus jandemarrai* as a possible branchiostegal; it seems unlikely that this small element provided a hyoidean gill cover. This element was considered as absent by [20, character 62]. *Onychodus* has been coded as (0). *Homalacanthus* has been coded as (1) [[16]; pers. observ.] and *Miguashaia* as (0).

28. Branchiostegal plate series along ventral margin of lower jaw: absent (0); present (1).
Homalacanthus has been coded as (1) [[16]; pers. observ.].

29. Branchiostegal ossifications: plate-like (0); narrow and ribbon-like (1).
Homalacanthus has been coded as (1) [[16]; pers. observ.].

30. Branchiostegal ossifications: ornamented (0); unornamented (1).

31. Branchiostegal ossifications: not imbricated (0); imbricated (1).
Character 31 and character states have been rephrased. *Homalacanthus* has been coded as (0).

32. Opercular cover of branchial chamber: complete or partial (0); separate gill covers and gill slits (1).

33. Opercular (submarginal) ossification: absent (0); present (1).
Dialipina is coded as (1) although a true opercular is absent [21, 22] but since the character takes into account the presence of an opercular (submarginal) ossification, such an element is present.

34. Shape of opercular (submarginal) ossification: broad plate that tapers towards its proximal end (0); narrow, rod-shaped (1).
Miguashaia has been coded as (0).

35. Gular plates: absent (0); present (1).

36. Size of lateral gular plates: extending most of length of the lower jaw (0); restricted to the anterior third of the jaw (no longer than the width of three or four branchiostegals) (1).

37. Basihyal: present (0); absent, hyoid arch articulates directly with basibranchial (1).

38. Interhyal: absent (0); present (1).

39. Oral dermal tubercles borne on jaw cartilages: absent (0); present (1).

40. Tooth whorls: absent (0); present (1).
Homalacanthus has been coded as (0).

41. Bases of tooth whorls: single, continuous plate (0); some or all whorls consist of separate tooth units (1).

42. Enlarged adsymphysial tooth whorl: absent (0); present (1).
Homalacanthus has been coded as (0).

43. Teeth ankylosed to dermal bones: absent (0); present (1).
Homalacanthus and *Miguashaia* have been coded as (0).

44. Dermal jaw plates on biting surface of jaw cartilages: absent (0); present (1).

45. Maxillary and dentary tooth-bearing bones: absent (0); present (1).

46. Large otic process of the palatoquadrate: absent (0); present (1).

47. Insertion area for jaw adductor muscles on palatoquadrate: ventral or medial (0); lateral (1).
Miguashaia has been coded as (1).

48. Oblique ridge or groove along medial face of palatoquadrate: absent (0); present (1).
Miguashaia has been coded as (1).

49. Fenestration of palatoquadrate at basipterygoid articulation: absent (0); present (1).

50. Perforate or fenestrate anterodorsal (metapterygoid) portion of palatoquadrate: absent (0); present (1).
Miguashaia has been coded as (0).

51. Dorsal process on Meckelian bone or cartilage: absent or weak (0); well-developed (pronounced) (1).
Character 51 and character states have been rephrased.

52. Preglenoid process: absent (0); present (1).

53. Jaw articulation located on rearmost extremity of mandible: absent (0); present (1).

54. Precerebral fontanelle: absent (0); present (1).
Gogonasus has been recoded as (0).

55. Median dermal bone of palate (parasphenoid): absent (0); present (1).

56. Position of nasal opening(s): dorsal, placed between orbits (0); ventral and anterior to orbit (1).

57. Olfactory tracts: short, with olfactory capsules situated close to telencephalon cavity (0); elongate and tubular (much longer than wide) (1).
Cheirolepis has been coded as (1) [23].

58. Prominent pre-orbital rostral expansion of the neurocranium: present (0); absent (1).

59. Pronounced sub-ethmoidal keel: absent (0); present (1).

60. Position of myodome for superior oblique eye muscles: posterior and dorsal to foramen for nerve II (0); anterior and dorsal to foramen (1).

61. Endoskeletal intracranial joint: absent (0); present (1).
Cheirolepis and *Miguashaia* have been coded as (0).

62. Spiracular groove on basicranial surface: absent (0); present (1).
Cheirolepis has been coded as (1) [23].

63. Spiracular groove on lateral commissure: absent (0); present (1).
Cheirolepis has been coded as (1) [23].

64. Subpituitary fenestra: absent (0); present (1).
Gogonasus has been recoded as (0).

65. Supraorbital shelf broad with convex lateral margin: absent (0); present (1).
Cheirolepis has been coded as (1) [23].

66. Orbit dorsal or facing dorsolaterally, surrounded laterally by endocranium: present (0); absent (1).

67. Extended prehypophysial portion of sphenoid: absent (0); present (1).
Homalacanthus has been coded as (?).

68. Narrow interorbital septum: absent (0); present (1).

69. Main trunk of facial nerve (N. VII): elongate and passes anterolaterally through orbital floor (0); stout, divides within otic capsule at the level of the postorbital process (1).

70. Hyoid ramus of facial nerve (N. VII) exits through posterior jugular opening: absent (0); present (1).

71. Glossopharyngeal nerve (N. IX) exit: foramen situated posteroventral to otic capsule and anterior to metotic fissure (0); through metotic fissure (1).

72. Short otico-occipital region of braincase: absent (0); present (1).
Homalacanthus has been coded as (?). *Gogonasus* has been recoded as (0).

73. Ethmoid region elongate with dorsoventrally deep lateral walls: absent (0); present (1).

74. Basicranial morphology: platybasic (0); tropibasic (1).

75. Ascending basisphenoid pillar pierced by common internal carotid: absent (0); present (1).

76. Jugular vein: invested in otic capsule wall posterior to the postorbital process (0); lateral wall of jugular canal incomplete or absent (1).
Cheirolepis has been coded as (1) [23].

77. Canal for lateral dorsal aorta within basicranial cartilage: absent (0); present (1).
Cheirolepis has been coded as (1) [23].

78. Entrance of internal carotids: through separate openings flanking the hypophyseal opening or recess (0); through a common opening at the midline of the basicranium (1).

79. Canal for efferent pseudobranchial artery within basicranial cartilage: absent (0); present (1).
Cheirolepis has been coded as (1) [23].

80. Position of basal/basipterygoid articulation: same anteroposterior level as hypophysial opening (0); anterior to hypophysial opening (1).
Homalacanthus has been coded as (?).

81. Postorbital process: not articulated with palatoquadrate (0); articulating with palatoquadrate (1).
Character 81 and character states have been rephrased from [2] and [1]. *Cheirolepis* has been coded as (0) [23]. *Homalacanthus* has been coded as (?).

82. Labyrinth cavity: separated from the main neurocranial cavity by a cartilaginous or ossified capsular wall (0); skeletal capsular wall absent (1).

83. Basipterygoid process (basal articulation) with vertically oriented component: absent (0); present (1).

84. Pituitary vein canal: dorsal to level of basipterygoid process (0); flanked posteriorly by basipterygoid process (1).

85. External (horizontal) semicircular canal: absent (0); present (1).

86. Sinus superior: absent or indistinguishable from union of anterior and posterior canals with saccular chamber (0); present (1).

87. External (horizontal) semicircular canal: joins the vestibular region dorsal to posterior ampulla (0); joins the vestibular region levelling with posterior ampulla (1).

88. Trigemino-facial recess: absent (0); present (1).

89. Posterior dorsal fontanelle: absent (0); present (1).
Cheirolepis has been coded as (1) [23].

90. Shape of posterior dorsal fontanelle: approximately as long as broad (0); much longer than wide, slot-shaped (1).
Meemannia has been recoded as (0).

91. Dorsal ridge: absent (0); present (1).

92. Endolymphatic ducts: posterodorsally angled tubes (0); tubes oriented vertically through median endolymphatic fossa (1).

93. Lateral otic process: absent (0); present (1).

94. Process forming part or complete wall of jugular groove or canal projecting from otic capsule wall: absent (0); present (1).

95. Position of hyomandibula articulation on neurocranium: below or anterior to orbit, on ventrolateral angle of braincase (0); posterior to orbit (1).

96. Ventral cranial fissure: absent (0); present (1).
Homalacanthus has been coded as (?).

97. Metotic (otico-occipital) fissure: absent (0); present (1).
Cheirolepis has been coded as (1) [23].

98. Vestibular fontanelle: absent (0); present (1).
Cheirolepis has been coded as (1) [23].

99. Occipital arch wedged in between otic capsules: absent (0); present (1).

100. Spino-occipital nerve foramina: two or more, aligned horizontally (0); one or two, aligned dorsoventrally (1).
Cheirolepis has been coded as (1) [23].

101. Ventral notch between parachordals: absent (0); present or entirely unfused (1).
Cheirolepis has been coded as (1) [23].

102. Parachordal shape: broad, flat (0); keeled with sloping lateral margins (1).
Homalacanthus has been coded as (?).

103. Hypotic lamina (and dorsally directed glossopharyngeal canal): absent (0); present (1).

104. Macromeric dermal shoulder girdle: absent (0), present (1).
The polarity of character 104 has been reversed because of the absence of macromeric dermal shoulder girdle in galeaspids and osteostracans. [1] consider that *Gyracanthides murrayi* lacks a macromeric pectoral girdle. However, based on the description of [24], the proper identification of elements from the pectoral region remains questionable. The presence of small tubercules similar to dermal ornamentation on the element identified by [24, fig. 7] as the scapulocoracoid suggests the potential presence of a dermal ventral component. Because of this discrepancy of interpretation, *Gyracanthides* has been coded as (?).

105. Dermal shoulder girdle composition: ventral and dorsal (scapular) components (0); ventral components only (1).

106. Dermal shoulder girdle forming a complete ring around the trunk: present (0); absent (1).
Chondrichthyan taxa have been coded (1) as they lack such a structure independently if they have or not dermal components.

107. Pectoral fenestra completely encircled by dermal shoulder armour: present (0); absent (1).

108. Median dorsal plate: absent (0); present (1).

109. Pronounced internal crista ('keel') on median dorsal surface of shoulder girdle: absent (0); present (1).

110. Scapular process of endoskeletal shoulder girdle: absent (0); present (1).

111. Ventral margin of separate scapular ossification: horizontal (0); deeply angled (1).

112. Cross-sectional shape of scapular shaft: flattened or strongly ovate (0); subcircular (1).

113. Flange on trailing edge of scapulocoracoid: absent (0); present (1).

114. Scapular process with posterodorsal angle: absent (0); present (1).

115. Endoskeletal postbranchial lamina on scapular process: present (0); absent (1).

116. Mineralisation of internal surface of scapular shaft: mineralised all around (0); unmineralised on internal face forming a hemicylindrical crosssection (1).

117. Coracoid process: absent (0); present (1).
Cheirolepis has been coded as (1) [23].

118. Procoracoid mineralisation: absent (0); present (1).
Cheirolepis and *Miguashaia* have been coded as (0).

119. Fin base articulation on scapulocoracoid: stenobasal (0); eurybasal (1).
Miguashaia has been coded as (0).

120. Perforate propterygium: absent (0); present (1).

121. Pelvic fins: absent (0); present (1).

122. Pelvic claspers: absent (0); present (1).
Homalacanthus has been coded as (0).

123. Dermal pelvic clasper ossifications: absent (0); present (1).

124. Pectoral fins covered in macromeric dermal armour: absent (0); present (1).
Miguashaia has been coded as (0).

125. Pectoral fin base has large, hemispherical dermal component: absent (0); present (1).
Miguashaia has been coded as (0).

126. Dorsal fin spines: absent (0); present (1).

127. Anal fin spine: absent (0); present (1).

128. Paired pectoral fin spines: absent (0); present (1).
Osteostraci has been coded as (0) as the pectoral fins of osteostracans are relatively well-known and lack such spines [4].

129. Median fin spine insertion: shallow, not greatly deeper than dermal bones/scales (0); deep (1).
Miguashaia has been coded as (-).

130. Prepelvic fin spines: absent (0); present (1).
Miguashaia has been coded as (0).

131. Prepectoral fin spines: absent (0); present (1).
Miguashaia has been coded as (0).

132. Fin spines with ridges: absent (0); present (1).

133. Median and paired fin spines with nodes: absent (0); present (1).

134. Dorsal fin spines with rows of large retrorse denticles: absent (0); present (1).
Miguashaia has been coded as (-).

135. Synarcual: absent (0); present (1).
Miguashaia has been coded as (0).

136. Number of dorsal fins, if present: one (0); two (1).
Miguashaia has been coded as (1).

137. Anal fin: absent (0); present (1).

138. Caudal radials: extend beyond level of body wall and deep into hypochordal lobe (0); radials restricted to axial lobe (1).
Miguashaia has been coded as (1). *Onychodus* has been coded as (1) [19].

139. Resorption and redeposition of odontodes: lacking or partially developed (0); developed (1).
Homalacanthus has been coded as (0).

140. Acrodin: absent (0); present (1).

141. Plicidentine: absent (0); simple or generalized polyplocodont (1).

142. Rostral tubuli: absent (0); present (1).
Cheirolepis, *Homalacanthus* and *Miguashaia* have been coded as (0).

143. Peg on rhomboid scale: narrow (0); broad (1).

144. Anterodorsal process on scale (sensu [25]): absent (0); present (1).

145. Fringing fulcra: absent (0); present (1).
Homalacanthus has been coded as (0).

146. Epichordal lepidotrichia in caudal fin: absent (0); present (1).
The fin elements present in the epichordal lobe of the caudal fin of *Bothriolepis* are interpreted as finrays rather than radials [26].

147. Dermal intracranial joint: absent (0); present (1).
Homalacanthus and *Miguashaia* have been coded as (0).

148. Large unpaired median skull roofing bone anterior to the level of nasal capsules: absent (0); present (1).
Homalacanthus has been coded as (0).

149. Nasals: absent (0); many (1); one or two (2).
A new plesiomorphic condition has been included in character 149 to take into account the absence

of nasals in osteostracans and galeaspids. The coding has been changed in *Acanthodes* [27], *Cheiracanthus*, *Brochoadmones* [28], *Euthacanthus* [29], *Ischnacanthus* [30], *Kathemacanthus* [31], *Obtusacanthus* [32], *Promesacanthus* [14], and *Tetanopsyrus* [33] for (0) because only head scales surround the nares. *Cassidiceps* [34], *Homalacanthus* [16], *Mesacanthus* [30], *Climatius*, *Culmacanthus* [35] and *Nerepisacanthus* [36] have been coded (2) because they have one or two pair(s) of nasal bones. *Ptomacanthus* has been coded as (?) because of the weak preservation of the snout area [37]. All chondrichthyans have been coded as (0). *Pterichthyodes* has been coded (0).

150. Mesial margin of nasal: not notched (0); notched (1).
Mesacanthus has been coded as (1) [30].

151. Dermintermedial process: absent (0); present (1).

152. Posterior nostril: associated with orbit (0); not associated with orbit (1).
Homalacanthus and *Miguashaia* have been coded as (1).

153. Position of posterior nostril: external, far from jaw margin (0); external, close to jaw margin (1).

154. Supraorbital [sensu [20] including posterior tectal of Jarvik]: absent (0); present (1).
Homalacanthus has been coded as (0).

155. Supraorbital, preorbital and nasal: unfused (0); fused (1).

156. Tectal [sensu [20], not counting the 'posterior tectal' of Jarvik]: absent (0); present (1).
Homalacanthus has been coded as (0). *Miguashaia* and *Porolepis* have been coded as (1).

157. Lateral plates [sensu [2]]: absent (0); present (1).

158. Location of pineal foramen/eminence: level with posterior margin of orbits (0); well posterior of orbits (1).

159. Parietals (preorbitals of placoderms) surround pineal foramen/eminence: yes (0); no (1).

160. Spiracle: not completely enclosed by skull roof bones (0); completely enclosed by skull roof bones (1).
Character 160 and character states have been rephrased.

161. Number of marginal bones alongside paired median skull roofing bones over the otico-occipital division of braincase: single (0); two or more (1).

162. Number of paranuchals: one pair (0), two pairs (1).
Miguashaia has been coded as (?).

163. Large unpaired median bone contributing to posterior margin of skull roof: absent (0); present (1).
Cheirolepis and *Homalacanthus* have been coded as (0).

164. Contact of nuchal or centronuchal plate with paired preorbital plates: absent (0), present (1).

165. Posterior process of the paranuchal plate behind the nuchal plate (dorsal face): absent (0), present (1).

166. Junction of posterior pitline and main lateral line: far in front of posterior margin of skull roof (0), close to posterior margin of skull roof (1).

167. Extrascapulars: absent (0); uneven number (1); paired number (2).
Character 167 has been redefined to include the new plesiomorphic condition.

168. Dermal neck-joint between paired main-lateral line-bearing bones of skull and shoulder girdle: absent (0); present (1).

169. Type of dermal neck-joint: sliding, dermal shoulder girdle plate with flat articular flange (0); ginglymoid, dermal shoulder girdle plate with articular condyle or fossa (1).

170. Number of sclerotic plates: four or less (0); more than four (1).

171. Foramina (similar to infradentary foramina) on cheek bones: absent (0); present (1).

172. Lacrimal posteriorly enclosing posterior nostril: absent (0); present (1).

173. Most posterior major bone of cheek bearing preopercular canal ("preopercular") extending forward, close to orbit: absent (0); present (1).
Eusthenopteron has been coded as (0).

174. Number of cheek bones bearing preopercular canal posterior to jugal: one (0); two (1).
Eusthenopteron, *Osteolepis*, *Gogonasus* and *Porolepis* have been coded as (1).

175. Bone bearing both quadratojugal pit-line and preopercular canal: absent (0); present (1).

176. Dermohyal: absent (0); present (1).

177. Premaxillae: without inturned adsymphysial processes (0); with inturned adsymphysial processes (1).
Character 177 and character states have been rephrased. *Miguashaia* has been coded as (0).

178. Premaxilla forming part of orbit: absent (0); present (1).

179. Preorbital process of premaxilla: absent (0); present (1).

180. Posterior expansion of maxilla (maxilla cleaver-shaped): present (0); absent (1). *Dialipina* has been recoded as (0) [22, 21].

181. Ventral margin of maxilla: straight (0), curved (1).

182. Maxilla: contributes to posterior margin of cheek (0); does not contribute to posterior margin of cheek (1).
Character 182 and character states have been rephrased. *Eusthenopteron* has been coded as (1).

183. Course of ethmoid commissure: middle portion through median rostral (0); sutural course (1); through bone center of premaxillary (2).
Eusthenopteron has been coded as (1).

184. Position of anterior pit-line: on paired median skull roofing bones over the otico-occipital division of braincase (0); on paired median skull roofing bones over the sphenoid division of braincase (1).
Eusthenopteron has been coded as (1); *Miguashaia* has been coded as (-).

185. Middle and posterior pit-lines on postparietal: posteriorly situated (0), mesially situated (1).
Eusthenopteron has been coded as (0).

186. Position of middle and posterior pit-lines: close to midline (0); near the central portion of each postparietal (1).
Eusthenopteron has been coded as (1).

187. Course of supraorbital canal: between anterior and posterior nostrils (0); anterior to both nostrils (1).
Eusthenopteron has been coded as (-).

188. Course of supraorbital canal: straight (0); lyre-shaped (1).
Eusthenopteron has been coded as (0).

189. Posterior end of supraorbital canal: in postparietal (0); in parietal (1); in intertemporal (2).
Eusthenopteron has been coded as (2).

190. Otic and supraorbital canals: not in contact (0); in contact (1).
Character 190 has been rephrased.

191. Supraorbital and infraorbital canals: in contact rostrally (0); not in contact rostrally (1).
Character 191 has been rephrased. *Eusthenopteron* has been coded as (0).

192. Otic canal: runs through skull roof (0); follows edge of skull roof (1).
Eusthenopteron and *Miguashaia* have been coded as (0).

193. Infraorbital canal follows premaxillary suture: no (0); yes (1).

194. Sensory canal or pit-line associated with maxilla: absent (0); present (1).
Eusthenopteron has been coded as (0); *Miguashaia* has been coded as (-).

195. Preopercular canal: canal exits the dorso-posterior margin of the preopercular (0); canal exits at the antero-dorsal margin of the preopercular (1); canal exits the anterior margin of the preopercular at half-length (2).
Character 195 and character states of [2] have been redefined. All codings have been changed according to the newly defined character states.

196. Median gular: absent (0); present (1).
The polarity of character 196 was reversed because a median gular is absent in galeaspids and osteostracans.

197. Foramen in hyomandibular: absent (0); present (1).

198. Large dermal plates forming outer dental arcade: only with denticles (0), with a monolinear series of large, shedding teeth (1).
Eusthenopteron has been coded as (1).

199. Tooth-bearing median rostral: absent (0); present (1).
Eusthenopteron has been coded as (0).

200. Teeth of dentary: reaching anterior end of dentary (0); not reaching anterior end of dentary (1).
Eusthenopteron has been coded as (0).

201. Number of coronoids (*sensu lato*, including parasymphysial dental plate but excluding parasymphysial tooth whorl): more than three (0); three (1).

202. Fangs of coronoids (*sensu stricto*): absent (0); present (1).
Eusthenopteron has been coded as (1).

203. Marginal denticle band on coronoids: broad band, at least posteriorly (0); narrow band with 2-4 denticle rows (1).
Eusthenopteron has been coded as (-).

204. Infradentary bones: absent (0), present (1).

205. Infradentary foramina: always present (0); variable (1); always absent (2).

206. Large ventromesially directed flange of symphysial region of mandible: absent (0); present (1).
[38] (character 156) coded *Miguashaia* as (1) although the condition is visible in *M. grossi* [39] but lacks clear evidence in *M. bureaui*. *Youngolepis* has been recoded as (0/1) since the condition of the flange is variable (Zhu Min, pers. comm.).

207. Flange-like extension composed of Meckelian ossification and prearticular that extends below ventral margin of infradentaries: absent (0), present (1).
[38] (character 159) coded *Youngolepis* as (0), whereas [2] and [1] coded as (1).

208. Strong ascending flexion of symphysial region of mandible: absent (0); present (1).
Eusthenopteron has been coded as (0).

209. Parasymphysial plate: absent (0); detachable tooth whorl (1); long with posterior corner, sutured to coronoid, denticulated or with tooth row (2).
The polarity of character 209 has been changed to take into account the plesiomorphic condition given by galeaspids and osteostracans. *Eusthenopteron* has been coded as (2).

210. Anterior end of prearticular: far from jaw symphysis (0); near jaw symphysis (1).
Eusthenopteron has been coded as (1).

211. Prearticular-dentary contact: present (0); absent (1).
Eusthenopteron has been coded as (1).

212. Meckelian bone exposed immediately anterior to first coronoid: yes (0); no (1).
Eusthenopteron has been coded as (1) [40].

213. Dermal plates on mesial (lingual) surfaces of Meckel's cartilage and palatoquadrate: absent (0); present (1).
Eusthenopteron has been coded as (1).

214. Biconcave glenoid on lower jaw: absent (0); present (1).

215. Contact between palatoquadrate and dermal cheek bones: continuous contact of metapterygoid and autopalatine (0); metapterygoid and autopalatine contacts separated by gap between commissural lamina of palatoquadrate and cheek bones (1).

216. Metapterygoid with developed mesial ventral protrusion (*i.e.*, commissural lamina *sensu stricto*): absent (0); present (1).

217. Course of mandibular canal: not passing through most posterior infradentary (0); passing through most posterior infradentary (1).

218. Course of mandibular canal: passing through dentary (0); not passing through dentary (1).
Miguashaia has been recoded as (1).

219. Internasal pits: absent (0); undifferentiated or anterior palatal fossa (1); shallow, paired pits with strong midline ridge (2); deep, pear-shaped pits (3).

220. Fenestra ventrolateralis: absent (0); present (1); common ventral fenestra for anterior and posterior nostrils (2).

221. Ethmoid articulation for palatoquadrate: placed on postnasal wall (0); extends posteriorly to the level of N. II (1).

222. Eye stalk or unfinished area on neurocranial wall for eye stalk: absent (0); present (1).

223. Developed postorbital cavity: absent (0); present (1).

224. Postorbital pila ascending from basipterygoid process to postorbital process: absent (0); present (1).

225. Unconstricted cranial notochord: absent (0); present (1).

226. Descending process of sphenoid (with its posterior extremity lacking periostegeal lining): absent (0); present (1).

227. Articulation facet with hyomandibular: single-headed (0), double-headed (1).

228. Hyoid arch articulation: on lateral commissure (0); on otic capsule wall (1).

229. Opercular suspension on braincase: absent (0); present (1).

230. Posterior postorbital process: absent (0); present (1).
Cheirolepis has been coded as (0) [23].

231. Basicranial fenestra: absent (0); present (1).
Cheirolepis has been coded as (0) [23].

232. Otical process (an outgrowth from the lateral wall of the braincase penetrated by the branches of the r. oticus lateralis): absent (0); present (1).

233. Lateral cranial canal: absent (0); present (1).

234. Midline canal in basicranium for dorsal aorta: absent (0); present (1).

235. Vomerine fangs: absent (0); present (1).
Miguashaia has been coded as (0).

236. Vomeral area with grooves and raised areas: absent (0); present (1).

237. Parasphenoid: protruding forward into ethmoid region of endocranium (0); behind ethmoid region (1).

238. Denticulated field of parasphenoid: without spiracular groove (0); with spiracular groove (1).
Miguashaia has been coded as (0).

239. Ascending process of parasphenoid: absent (0); present (1).
Miguashaia has been coded as (0).

240. Shape of parasphenoid denticulated field: broad rhomboid or lozenge-shaped (0); broad, splint-shaped (1); slender, splint-shaped (2).
Miguashaia has been coded as (0).

241. Parasphenoid denticulated field: without multifid anterior margin (0); with multifid anterior margin (1).
Character 241 and character states have been rephrased. *Miguashaia* has been coded as (0).

242. Parasphenoid denticle field: without anteriorly divergent lateral margins (0); with anteriorly divergent lateral margins (1).
Character 242 and character states have been rephrased. *Miguashaia* has been coded as (1).

243. Parasphenoid denticle field: terminates at or anterior to level of foramina for internal carotid arteries (0); extends posterior to foramina for internal carotid arteries (1).

244. Presupracleithrum: absent (0); present (1).

245. Anocleithrum: absent (0), element developed as postcleithrum (1); element developed as anocleithrum sensu stricto (2).

246. Cleithra: absent (0); two cleithra (1); one cleithrum (2).
Character 246 as used by [1] and [2] and originally by is redefined to minimize the assumption implied in the apomorphic state. For example, the original coding of *Eusthenopteron* as (1) implied that the pectoral girdle element identified as the cleithrum included the dorsal and ventral cleithra [i.e., the anterolateral (AL) and anterior ventrolateral (AVL) plates of placoderms, respectively) as well as the pectoral spine (i.e., spinal plate of placoderms), where in fact there is no indication of fusion. The character is rephrased to take into account the absence of cleithrum, the presence of two cleithra (including the AL and AVL plates of placoderms) or a single cleithrum. A new plesiomorphic condition is added to take into account the scope of the analysis. Character 128 takes into account the presence of pectoral spines, whereas character 265 has been added to take into account the presence of the spinal plate.

247. Relationship of clavicle to cleithrum: ascending process of clavicle overlapping cleithrum laterally (0); ascending process of clavicle wrapping round anterior edge of cleithrum, overlapping it both laterally and mesially (1).

248. Triradiate scapulocoracoid: absent (0); present (1).

249. Subscapular foramen/fossa: absent (0); present (1).

250. Endoskeletal supports in pectoral fin: multiple elements articulating with girdle (0); single element (”humerus”) articulating with girdle (1).

251. Pectoral propterygium: absent (0); present (1).

252. Pelvic girdle with substantial dermal component: absent (0); present (1).
The polarity of character 252 has been reversed.

253. Pelvic fin spines: absent (0); present (1).

254. Articulated jaws: absent (0); present (1).

255. Endocranial optic fissure: absent (0); present (1).

256. Admedian fin spines: absent (0); present (1).

257. Fin spine insertion: smooth (0); with fine parallel longitudinal ridges (1).
Character 257 and character states have been rephrased.

258. Retrorse denticles on adult pectoral spine: absent (0); present (1).
Character 258 has been rephrased.

259. Sclerotic plates: present (0); absent (1).

260. Areal growth in postcranial scale crowns: present (0); absent (1).
Character 260 of [1] has been divided into characters 260 and 265 to take into account the two modes of growth separately. Areal growth characterized polyodontode scales with addition of odontodes centripetally relative to the first odontode [as in type B2 growth of chondrichthyan scales [41]]. Areal growth of osteostracan scales represents the plesiomorphic condition [42]. *Climatius* [43] and *Ptomacanthus* [37] have been coded as (1).

261. Scales with a canal system in the dentine of the crown, opening out through pores on the crown surface: absent (0); present (1).

262. Lepidotrichia-like scale alignment: present (0); absent (1).
Character 262 corresponds in part to character 7 of [2] and [1]. The presence of lepidotrichia-like scale alignment has been documented in osteostracans [see *Ilemanaspis* [42] and *Escuminaspis* [4]]. Fin webs preserved in specimens of *Parexus* and *Rhadinacanthus* show this type of scale alignment. *Triazeugacanthus*, *Homalacanthus* (MHNM collections) and *Acanthodes* (CMN and NHM collections) show this alignment. Lupopsyrus (Hanke and Davis 2012) and *Obtusacanthus* [32] were previously coded as (0&1), but scale alignment are visible in the anterior part of the fin web.

263. Appositional growth in postcranial scales: absent (0); present (1).
Appositional growth characterized the apposition of odontodes on the side, on the back or on the front of the first odontode in polyodontode growing scales [growth type C, [41]]. Character 263 is complementary to character 260; it takes into account part of character 265 of [1]. *Parexus* [44] and *Kathemacanthus* [45] have been coded as (1).

264. Hypermineralized superficial layer of scales: present (0), absent (1).
Character 264 and character states have been modified from [46] (character 82). Because *Tremataspis* (Osteostraci) shows the presence of a hypermineralized layer [5], the polarization is modified from [46].

265. Hypermineralized superficial layer of scales: enameloid (0), enamel (1).
Tremataspis (Osteostraci) shows the presence of enameloid [5], which represents the plesiomorphic condition.

266. Enamel: one layer (0), multi-layers (1).
Mimipiscis [47], *Moythomasia* [48], *Cheirolepis* and *Triazeugacanthus* have been coded (1) because they have multi-layered enamel (=ganoine).

267. Spinal plates: absent (0), present (1)

Matrix:

Osteostraci:
0001000000?000?0?100000-000-----0-0----0--?----------00000000-?0000000-000?0----?0??00-00-
?00??000-??000-00-0??--?0-100-0-000?00-00---00??---0-00?000----0-00?----0--?0-----0--0---------
-----?----0-------0----0---------?0??0-??---?0???---------000-???0-00000-0000000-0
Galeaspida:
0?00-0-??00001?0?0&1000?0-000-----0-0----0--?----------000000?0-?0000000-000?0----?0??01?0
0-?00??000-0000--?----?--?------0-?--??0-?----0???---0-00?000----0-001----0--?0-----0--0------------
--?----0-------0----0---------?0???-??---?0???---------000------000---??01??--0
Acanthodes:
000120011011111-0110-?00?01110000-0-1?0----0011101010?01?1??010?0111?0110111010011
001101101001111?001100-1-0-1010010110010-0011110001000100--?-00---0--??0-00?----0--?0-
-1-----0----------??-00---?00------0----0---00-1--?????0?001?00??0---------000-?10101100000-00-0
0-0
Achoania:
001121?????????1?0-????10?????????????11?1?11??????0?011010?1??00100???1?????0???????
??????????1???????10?1???????????1????????1???????????0?0????1???110???01?????????????0
????100???????1010???1???1110??10101111011????30110111?????????010?0010??????01?010
0?-?????010?
Akmonistion:
10012001?0-??21-?1?1-----000---10-0-?01110-0011?000?0101010?01001110101100?111?11?00
1??110111010100?0010-1-0-1-00110111011000100100000000?0??-0----0--?----0?-------?---?---
-------------?????---?00------0-?-??---0?-1--020?0????1?00?00---------000-??01?01000-??01?0100
Austroptyctodus:
0001?0?1?00000?1?0-?1-001-00---0110-0010-01100???000?01010?0????101????????0????????
?????????0???0??001001101-00000100?111001000?000011??????-0??00?????0-00?10100--1-11
?------------?-???0???0---0?------0---??---0??0--??????????1??????????????0?1???????1?00-0?0???
??0
Bothriolepis:
0000-01??00--?10?0-000010-00---0100-???0--?10000??00???0?0???????0????00????????0?????
???????0????????100010?????1--00-0--11000-00---000?????-0-1?10??0?0-010-0101-0?011?0-??
?0------?-???????0---??------0---00---0?00--?0??????????????????????0?2????0--110--0-0?-1--0
Brachyacanthus:
000100?1000?010??100??-0?11000?0??0-??0----00?????????01?1???????????????????????????
?????????????????11110-1000010?0??1?-00111011110011?0--?-00---?--??0-00?-------?---1--?--?-
------?--??-??---?0?------0----0---0???--????????????????0--------000-????011?1000?00????0

Brindabellaspis:
000110?100111????0-?000?1-?????010???????????????????0?010000000000000-00000100?00??
10000-00010000-0?0010????1-0001010??????0?????0?--???????0-?-?01???0?0-00010111100-11
??-???????????-???0?000--????????????????-????--02?100????11??00?????????????0?????110?
-?-0?-???1
Brochoadmones:
0101000100?1010-0110??-0?000---10-0-??1100000?????????01?1???????????????????????????
?????????????????0-1-0-??????--?0?10-0011111111111011?0-0?-00---0--??0-00?-------?-------------
------??-??---?0??-----0----0---0?-?--????????????????0--------000-????011?0001-00-1--0
Buchanosteus:
00?1?0?100111??0?0-001111-00---0110-??1--0?10010??0?0011010000001010010-00000000?00?
?1000???0011000-0100100011?0?--1?1???????00?0??000-???????0-?-?00?????0-00010101011-1
1??-????------?-???????0---??------0---00---0000--02??1000001100000010000010010??????110--
0-0?-???1
Campbellodus:
000??001?00000?1?0-?1-001-00---0100-0010-01100?0??000?0??????????????????????????????
????????0????????100110???--1-?0??111001000?0-001?01????-?--?0?????0-00?10100--1-11?----
--------?-???0???0---0?------0---??---00-0--??????????1??????????????001?????1?1?0--0-0?-???1
Cassidiceps:
0001?00??0111100?10???-???111?10??0-??0----00?????????01?1????????????????????????????
????????????????0---0-110????????10-0011101001001 1?0--?-00--02--??0-00?----0--?0--?--?--?---
----?--?????---?0?------0----0---0???--????????????????0--------000-????011?000?-00-???0
Cheiracanthus:
000120011011110-?111-?00?01110000-0-??0----0011001010?01?1???????1?????????????01????
??????????????????0-1-0-1110010010?10-0011110001000 1?0--?-00--00--??0-00?----0--?0--1-----
0----------??-??---?0?------0----0---0?-1--????????????????0--------000-????011?0000-00-???0
Cheirolepis:
0011001110111112110-01-110&1-1100101011??10-001111?10000?11110?011?011????101?1000
00?00????1?????111?11??10110-?????1?101010-00000-00---001?0000-011?0200101000011 01
0--?20-000100100&10000??0?0000100101010000011000000?111100?0??????0??00??10?00010
1?0&11201?0100100--0-0--0110
Chondrenchelys:
11012001?0???-1-?1?1-----000---00-0-??1100-0011?00011001110?0?000110??-111??0?????????
????0?00110?0?0010-1-0-1-00110100-11000000-00---000?????-???--0--?----0?-------?---?--?--???
?----?--?????---?0????---0-?-???--???1--????????????????---?-??--000-??????100--????????0
Cladodoides:
110????????????-????---?-?????????????111?-0011?00???1010???0000011010100001111111001
1011111?0101010001??????????????????????????????????????-?----0--?----0?-------?---?--?--??
??-------?????---??????---0-?-???--0?-1--02?100???1?0??00---?-??--???-??????100?-??0??????
Cladoselache:
1101200100-??2?-?1?1-----000---10-0-?01110-00110000?0?01?10?0??0111????10???11?11?????
??1?1?101010??10?0-1-0-1-0011010101?-001000000?00000????-?----0--?----0?-------?---?--?--??
??----?--?????---?00???---0-?-???--???1--?????????1??????---?-??--000-????001000-0?0?????0
Climatius:
000100000000010-?100?-00?11100?0??0-??11000001110000?0?0??1???????1?????????????????
?????????????????11110-100001000??10-0011101111001 1?000?-00--02--??0-00?----0--?0--1--
?--?----------??-??---?0??-----0----0---00?1--????????????????0--------000-????011?100000111--0
Cobelodus:
1101?00??0-????-?1?1-----000---10-0-001110-0011?00010101?1??01000111101101011111111100
1101101100101000001 0-1-0-1-0011011101100000 0-00---0000????-???--0--?----0?-------?---?--?-
-????----?--?????---?0????---0-?-???--???1--02?1?0?0????0?00---?-??--000-??????1?0?-??0?????0
Coccosteus
000??00?0??????0?0-001111-00---0110-??10-0110??????00?11?1??????????????00?????????????

?????0????????????100011?0?--1?????1?000000-0010-100?????-?--?0?????0-0001010101011011?0-
???0------?-01?000?0---0?------0---00---000?--?????????????????????????001?????00110--?-0?-??
?1
Cowralepis:
000??0?????????0?0-1?-0-1-0????0110-0?10-0110?????00??1???????????????????????????????
????0???????????100110????????01?1?000000-00?0?1-00????-?--?0?????0-00?10101111010??-?
???------?-???010?0---0?------0---00---00??--?????????????????????????001?????001?0--??0?????
1
Culmacanthus:
?001?00110111101?100???1?000---0??0-??0----10?????1???01?1????????????????????????????
????????????????11110-1011010???10-0011110001001 1??--?-00--02--000-000----0--?0--?--?--0
----------??-??---???------0----0---0???--????????????????0--------000-????011??101-?0-???0
Debeerius:
1101200??0???-1-?1?1-----000---00-0-00101?-0001?000?1001110?0?0?0111??-111??0?110?????
?10-0100100?0?00?0-1-0-1-00110100011?001-01000100100????-???--0--?----0?-------?---?--?--?
???----?--?????---?0????---0-?-???--???1--????????????????---?-??--000-???00?1?00-?????00-0
Diabolepis:
00?121?????????1?0-?1-1???????????????10-0011?????000011?10?0?100110???101?1?0?0?1??1
1??????00111???1001????0?1011110?00101??????0-??
1????000???11011110?11????100---11000010?????1101000??0?0?0?0??010101011??????????1
00?-?????010?
Dialipina:
0??1?01?010001?0?0-01-101??????00?10??1?-00?1??????????1?1??????????????0?????????????
?????????????????10110-???????????10-00000-00--??11?0??????1??20??00/1?00?110???-??0-?0
????????00????01010?0???1?10????11??0????????1????????????????????????????10?????01?0--
?-0?-0100
Dicksonosteus:
00?110?1?0?????0?0-001111-00---0100-??1??0110000????001101000001010010-00001000?00?
?10000-0001100000?0010001000?--1-101????000?0-?010-1??????0-???00?????0-00010101011-
1100-???0------?-0/11?01000---00------0---00---0000--0211100000110000001000001001000 0???
110--0???????1
Diplacanthus:
00010001101001 0?0100-?01?000---00?0-??0--0-10?????0???01?1???????????????????????????
????????????????11110-111100001??10-001111100100 11?0--?-00--00--??0-00?----0--?0-----?--
0----------??-0?---?0?------0----0---0???--????????????????0--------000-?1??011?1111-10-1--0
Doliodus:
1?01?0000000???-?1???--0??00---?0?0-??1110-0011??????101010?00?00010???00??111?11?00?
???11??10101000?0?0-1?????????????????00??1???11?????000?-??--00--?----0?-------?0--?--?--?
----------?????---?0??-----0-?-???--0?-1--02??0????1?0??00---------?-0-????0?10?00?-0?-???0
Entelognathus:
000??0???0000??0?0-01-111-110010101???1?????101?????00?1010?0000010????00?01????0???
????????011000??10?100010??????????????00??0?????0????????-???00????00-00??0111-00?10
000???0000?1??????????00??1?0?????110?0??????01??0???1??0?01100?00????????0010??????
1?0?-0?0?????1
Eusthenopteron:
0011201100000 12100-01-110-1100101010111 0-0011111000000111101111001101101011 10000
?1111111100?0011110101010110-0------000-10-00000-00---0111?010-00110101--10100001110-
?10-10-01000001011101-02100?021110001-11??021111?11111100001010001000100002000022
1111000100--0-0?-00&100
Euthacanthus:
0001000110110 10-0100-?00?01100100-0-??0--0-0011???0?0?01?1???????1???????????????????
??????????????????11110-10100100???10-001110110100 11?0--?-00--00--??0-00?----0--?0--1----
-????-------??-0?---?0?------0----0---0?-?--????????????????0--------000-????011?0000-00-???0

Gogonasus 0011211100000?2100-01-110-1101?010101?10-0011111000000111101110001101 10
101110000??1111?1????001111010101010110-?????????0?????0??0??????0?1?1010100?10101--1
0100001110-?10-10-?10000010????????1????1?1???0??1??????????1111110000101000100010 0
002000002 1111?00100--0?0?????0
Guiyu:
0011201101000121?0-01-110-1100101010??11-1011?????000011?10?1???01001??10?1??0?0??
??????????????1???????10?110?--?-1?????1??001?1000010?11?000001??10200101000100111 0-?
10-?011000000000?001000?0?110?11101111001?11011??1?301?0110???0?????01000000122 0
????11100?-??0??0101
Gyracanthides:
000120000?100?--?11?-?-0-000---10-0-??0--0-00?????????0????????????????????????????????
?????????????????-0-100001001??10-00111101100011?0--?-??---?--???-?0?-------?---------0-------
---??-??---???------0----?---0???--????????????????---------??0-????011?1001-00-???0
Hamiltonichthys:
11012001?00?020-?110---0-000---10-0-0?1110-0001?0?010101?11?00??0110??-?0??1???1?????
???110?0?10001??010-1-0-1-0001010001100010010001101 10????-00---0--?----0?-------?---?--?--
?----------?????---?0??-----0-?-???--00?1--0???0????1?0??0?---------000-???1?01000-??0?????0
Homalacanthus:
0001000110111 11-0111-?00?01111000-0-??00-0000110010?0?01?1??????01???????1??????????
??????????1????????0-1-0-1110010?1??10-0011110001000100--0-00-002--??0-00?----0--?0--0--?
--0----------??-0?---?0?------0----0---0?-1--????????????????0--------000-????011?0000-00-00-?
Howqualepis:
?????01?0100012110-01-110-1100101011?1110101111?1??00?11?10?0110?111???101?????0??
??????????0?1111???1010110-?-???1??01110-00000-00---?011010?0110002100001000010110-?
20-0001001?1100000?00000100101011?00011001000?1?110000????0?000?0??1000111111112
0??01001?0--0????????
Ischnacanthus:
0001200110111 10-0100-?00?01101000-0-??1101110111000 10?0??1???????1?????????????????
??????????1????????0-1-0-111001101??10-0011110001001 1?000?-00--00--??0-00?----0--?0------
--0----------??-0?---?0??-----0----0---00-1--????????????????0--------000-????011?0001-00-????
Kathemacanthus:
0101000000?001?-?110-?00?000---00-0-??0----00?????000?01?1??????????????0??????????????
????????????????0-1-0-??????-?00?10-0011101001001 1?0--?-00--00--??0-00?----0--?0--------0---
-------??-??---?0?------0----0---0?-1--????????????????0--------000-????011?10011000???0
Kenichthys:
00?121?101000??100-01-11?-??00101010??10--011?????000011??0?1?100110???1011????0??1
1??????????111??????10110-0----1-000-??????????????????101010??1??0111101001011 10-?10-
?1100100001011101112100 10-1?100010111002110????1111000?10???0?????0000200 1001????
???100?-?????????
Ligulalepis:
001??0?10??????1?0-?1-1????????????????????????????0?101010?1?011010010??1?00001001
1?1100000111??????????????????????????????????????0???????0??????0?0??10????-??0-???
????????????00?000?0?????1???????????????????????101?000?0??1???????????????????10???
??0??010?
Lodeacanthus:
000100011011110-?110??00?01111100-0-??00-0000111010 00?01???????????????????????????
1???????????????????0-1-0?100?0?000??10-0011111010000 1?0--0-00-0021???0-00?-?--0--?0--1?
----0-------?-??????----0?------0---00---0?-?--???0??????-?????0--------000-??-?011?0100-00-0??0
Lophosteus:
???120?101?????0?0-?????????????????????????1??
???0???-???????????????????????????????????
????????????????????0???????????1???1??0??-0?-1--
?

Lupopsyrus:
000100010?10020-??1?-?00?01010?00-0???0--0-00?????????0??1???????????????????????????
?????????????????0-1-0-100001001??10-00111011110011?0--?-00--0?--??0-00?----0--?0--------0-
---------??-??---?0?------0----0---0???--????????????????0--------000-????011?0011-00?1--0
Macropetalichthys:
00?110?????????1?0-?00001-???????0?????0?????????????001100?0000000000-0000?1-1?0???1
0?00-?0010000-?00?10?1????????????????????1???10????????0-???00?????0-000101111000???
?-????------?-???0?0?0---??------0---??---????--???100????110000?????????001???????1??-??????
????
Meemannia:
00?121?????????1?0-?1-1???????????????1?-0011?????000???????0?????????01?????????1??11
??10??00111??1??????????????????????????????????????0???????0??????0??0?100????????????
????????????0?010???????1??0??10????????????????????????0??1????????????????????1?0?-??
???010?
Mesacanthus:
000120011?11110-?100??00?01110000-0-??0----0011?000?0?01?1???????1?????????????0?????
??????????????????0-110-111001100??10-00111010010001?0--?-00--021-??0-00?----0--?0--1----
-0----------??-0?---?0?------0----0---00-1--????????????????0--------000-????011?0000-00-???0
Miguashaia:
00112011000001210 0-01-111-00---01010??10-001111100?00?11?1??0????1?????????????????
??????????????????10110-????????00?10-00000-00---0111?000-0010010?101010?101?10-?10-
10?011000?---?-11?021?00-10?100000111100 11?11??11??????1?????????0??00001?0210????00
1?0--0?0??0100
Mimipiscis:
00112011010001211 0-01-110-11001010111110-0011111100000111101011001111101011110 00
0100111110000011110111010110-0----1-001110000000-00---0011010001100021000010000101
10-?20-000100101101000010000100101110000011000000111110000000100000000110001011
011120100100100--0?0??0110
Moythomasia:
0011201101000121 10-01-11?-1100101011?110-0011111100000111101011001111101011110 00
0100111110000011110111010110-0----1-001110000000-00---0011010001100021000010000101
10-?20-00010010110100001000010010111 0/10000110000001??110000000?0000000?11000111
1111120100100100--0?0??0110
Nerepisacanthus:
00010000&11011110-?11?-?00?000---10-0-??11?1110?????0?0?01?1???????1????????????????
????????????????????0-110-101001100??10-00111000010?11?000?-00---2--??0-00?-------?---?---
--?----------??-??---?0??-----0----0---0??1--????????????????0--------000-????111?000100?01--0
Obtusacanthus:
01010001-0?0020-?110-?00?000---00-0-??0----00?????????0??1?????????????????????????????
???????????????0-1-0-??????-?0??10-00111001110011?0--?-00--00--??0-00?-------?0--------0-----
-----??-??---?0?------0----0---0?-?--????????????????0--------000-????011?0001-00?1--0
Onychodus:
00112011000011210 0-01-111-00---01010?11101011111 0??00?11?10111?001001-?10111?0?0?1
001???????0?1111????10110-0----1-000?10-00000-00---0111?00?-0011010010101 0?101110-?1
0-10101101000000011110?100011001010011100111101 1?1113010001 0000???0??010010000220
001000100--0-0??0100
Onychoselache:
1101?001?0-00-0-?1?1---0-000---?0-0-??1110-0001?0?0?0101?1??0????11?????0???????0??????
???????????1????0-1-0-1-10010100011000100100010011 0????-0-?--0--?----0?-------?---?--?--???
?----?--?????---?0????---0-?-???--???1--????????????????---?-??--000-???10?1000-?-0?-0100
Orthacanthus:
1101200????????-?1??---0-?00---10-0-001110-00111000101010?1?0000011010100001111111 00
110111111010101000 10-1-0-1-0011011??11000100?00??10-10????-0??--0--?----0?-------?---?--?

--????----?--?????---?00???---0-?-???--???1--?????????1??????0--?-??--000-??????1000-?????1--0
Osorioichthys:
00?1201?0????1?110-01-11?-1100101011??1???0?1??????????1??????????????????????????????
?????????????????10110-??????????????00??0-00---????010?0????0210000100?1001????20-000
1??1?1100?0???0000?00?11?10?00?110?00???????00?????????????????????????1120??????1?0?
-0???????0
Osteolepis:
001121110100012100-01-11?-1100101010??10-001111?????0011110?1?10011011?101110??00
11?1111??0?00111?0101010110-???????????10-00000-00---?11110?0100110101--10100001110-
?10-10-?1????????????????1????2111??0??11??????????111110000101000100010000200002211
11?00100--0?0??0100
Parayunnanolepis:
0??1?0?1?0?11?10?0-000010-0----0100-????????0??????????0??????????????????????????????
????????????????100010?????????0?10010000?00---?00?????-?--?1???0?0?010-0101-0?-11??-??
?0------?-???????0---0?------0---??---????--?0???????????????????????002????010110--????????0
Parexus:
000100000000010-?100???0?11?0000??0-??1100000?????????01?1???????????????????????????
??????????????????11110-101001000??10-00111011111011?000?-00--0?--??0-00?----0--?0--1--?
--?-------?--?????---?0??-----0----0---0???--????????????????0--------000-????011?100010001--0
Poracanthodes:
00010&20010011110-?10????0??????????0-??110111011?000?0?????????????1???????????????
?????????????????????0-1-0-111001100??10-00111000010011?000?-00---?--??0-00?-------?---?--
---?-------?--??-??---?0??-----0----0---00-1--????????????????0--------000-????011?0001-10-1--0
Porolepis:
001121110100012100-01-110-110?101010?1110101111100000011110?11?0011011?1011100?0
??101??1????001111??01?10110-0----1-000?10-00000-00---?11?1010100110???10??10??00110-
??0-???01??000?????1?1??10???2111?11101???0?11?11111121?0001??000???0???1010???2???
?1000100--??0??????
Powichthys:
001121?10100012100-01-11?-1100?0101???11??011?????000011110?111001101??101?1?0?0??
10????????0011110?01010110-?????????????????????????????101111??0010111101011011????
10-??10??0000101110111100010?1?101110101001110????1121000?1110?011?0101101001021
111100?100--?????????
Promesacanthus:
00012001101111?-?101??00?01110000-0-??0----0011???010?01?1???????1???????????????1????
??????????????????0-1-0-11100?001??10-001110110100 01?0--?-00--00--??0-00?----0--?0--?-----
0----------??-??---?0?------0----0---0?-1--????????????????0--------000-????011?0000-00-???0
Psarolepis:
00?121?101?0??21?0-01-?10-?????????????11?1011?????000011010?1?10010011?10?11?0?0?10
01???100000111??????10?1??????-1??01?????01?10?0010????000001??10??110???0100??10-??
0-?1010??1110000101100 00?011??1110111010111101 1??113011011000?0001??01000000??1/2
00001111000-??0??0101
Pterichthyodes:
0?0??0-1?0???010?0-00??10-0?---0100-??0--???0???????????0?0???????0??????????????????????
??????0?????????1?0010?????1--00-0--11?00?00--?000?????-?--?10??0?0-010-0101-0?011??-???0
------?-???????0---??------0---??---????--?????????????????????????002???????110?-??0?????0
Ptomacanthus:
?00100?00011010-?100??00?01?0001??????1100-00110000???01?10??0????0????0????0000???
?????????????????10?11110-100001010??10-00111011110011?000?-00---?--??0-00?-------?---1--
?--?----------?????---?0??-----0-?-???--0?-1--?????????????0??00--------000-?????11?00000001???0
Pucapampella:
110??0?????????-????---?-????????????????????????????????1????000001101?1100?1101011001?
0?101010111?00100??-???-?0--?----??-???????---?--?--??

??-------?????---??????---0-?-???--????--?2?10????1?0??00---?-??--00?-??????10????????????
Rhadinacanthus:
0001000110111110?0100-?01?000---0??0-??0--0-10?????0???01?1?????????????????????????????
?????????????????11110-111100001??10-0011111001001 1?0--?-00--00--?-0-00?----0--?0-----?--
0----------??-??---?0?------0----0---0???--????????????????0--------000-????011?1111-00-1--0
Rhamphodopsis:
000??0???????0?1?0-?1-001-00---0110-??10-01100????????0??????????????????????????????????
???????0????????10011??0?--1-????111001000?0110?10?????-?0??0?????0-00?1010??01-11?----
--------?-???0???0---0?------0---??---0???--??????????1??????????????000??????01?0--??0?????1
Sigaspis:
0????0???0???010?0-00111?-00---010??????????0??
???????????????100010?????????1?1?0000?0??000-?????????????0?????0?00?10101000?10?0-?
??0------?-11?????0--?0?------0---??---????--??????????????????????????000?????101?0--??0?????
1
Styloichthys:
00?121?101000?2100-01-11?-????????????11??011????000011110?1?1001101?0101110??0?11
01?????0?0011100101010110-0----1-000-???????0??????????00?011??1???01????00?01110-??0-
?1?0010??0101110111200?101??101???100101110????1121010?1110?01??0?00001011??21111
0??100?-?????010?
Tamiobatis:
1101?0?000-00??-?11?---0-?????????????1110-0011?000??101??0?000001101010000111111?00
1???11111010100000 1?-1-??????1???????????1?01??110???00???-???--0--?----0?-------?---?--?--
????----?--?????---?????----0-?-???--???1--02???0?0?1??0?00---?-??--000-?????11?00-?10?1???0
Tetanopsyrus:
0001200110111110-?110??00?000---00-0-??10-0010010001?0?01?1???????????????????????????
???????????????????0-1-0-100001?010?10-0011100011001 1?0--?-00--00--??0-00?----0--?0--------
0----------??-??---?0?------0----0---00-1--????????????????0--------000-????011?0001-00-???0
Tristychius:
11012001?0-??-?-?1?1-----000---00-0-?01110-0001?0?010101010?000001101?-1000101?-0?001?
????11101000?10010-1-0-1-00110100011000100100011 0110????-???--0--?----0?-------?---?--?--?
???----?--?????---?0???----0-?-???--???1--????????????????---?-??----0-???1001000-????????0
Triazeugacanthus:
0001000110111110-111???00?01011000-0-??00-0000?1???????01???????????????????????????????1
???????????????????0-1-0???0?0?000??10-0011111010000 1?0--0-00-0021???0-00?-?--0--?0--0?--
--0-------?-??????----0?------0---00---0?-?--???0??????-?????0--------000-??-?011?0000-00-0110
Uraniacanthus:
0001000110111110-?100-?01?01000100-0-??0----10?????1???0??1????????????????????????????
??????????????????0-1-0-1011000?00?10-0011111111 0?11?0--?-00--0?--??0-00?----0--?0--------0-
---------??-??---???------0----0---0?-?--????????????????0--------000-????011??101-1?-1--0
Vernicomacanthus:
000??0???????1?-??0???0???100??0??????11?0000?????????0???????????????????????????????????
???????????????????110-1?000???1??10-0011101111001 1?0?0?-00---?--??0-00?-------?---?--?--?--
-----?--?????---????-----0----0---0???--????????????????0--------000-?????1101010???????0
Youngolepis:
00?121?101000?2100-01-11?-?????0101???11??01111???000011110?011001101101011100?001
1011111?000011110101010110-0----1-000-???????0??????????101111??0?10111???00101110-?
10-?110010000101110111100110-1?100110100&1101110???11121000?1110?0010011010100/1
1??211110??1?0?-?????0100

Results

As in most analyses using large data matrix for fossil taxa, the high proportion of unavailable coding (both “?” and “-”) is most likely a major source of phylogenetic ambiguities: 48.3% (47 taxa and 134

characters; [11]), 49.6% (60 taxa and 138 characters; [49]), 57.1% (79 taxa and 267 characters; this study), 60.1% (77 taxa and 262 characters; [1]), 61.5% (75 taxa and 253 characters; [2]). Although we are dealing with more than 50% of missing data, we recovered a phylogenetic signal showing that acanthodians are stem chondrichthyans.

Considering the large size of the data matrices, very few characters were dealing with the morphology, histology, growth and organisation of the scales. Brazeau [11] used 12 characters (4-6, 8-16) out of 134 characters, Davis et al. [49] used 11 characters (4-6, 8-15) out of 138 characters, and Burrow et al. [1] used 16 characters (4-6, 8-15, 139, 143, 144, 260 and 262) out of 262 characters. We used 20 characters (relative to scales) out of 267. Scale histology characters 8 (condition of scale growth pattern with polyodontode or monodontode), 9 (concentric growth pattern), 260 (areal growth pattern) were redefined (see List of characters). Polarity of scale characters 8 (polyodontode or monodontode), 11 (body scale profile), and 13 (flatten base of body scales) were changed. Characters 263 (appositional growth pattern), 264 (hypermineralized superficial layer of scale), 265 (type of hypermineralized tissue), and 266 (single- or multi-layered enamel) were added. Fourteen out of the 20 scale characters are relevant for acanthodians. The deletion of any one of these 14 characters has a significant impact on the resulting topology (S7 Figure - S12 Figure). The deletion of 8 of these 14 characters [12 (bulging base), 13 (flat base), 14 (flank scale alignment), 127 (anal fin spine), 128 (paired pectoral fin spines), 260 (areal growth), 263 (appositional growth), and 265 (enamel/enameloid)] makes the acanthodians and putative chondrichthyans as a monophyletic group closely related to chondrichthyans (S3 Table). The number of steps to obtain the monophyly of the acanthodians plus putative chondrichthyans varies between 703 and 713 (S3 Table), which is a minor difference from the 711 steps of the complete analysis. The deletion of two of the fin spines characters [character 127 (anal fin spine) and character 128 (paired pectoral fin spines)] also led to the monophyly of the acanthodians, with trees being only one and four steps shorter, respectively.

References

1. Burrow CJ, Den Blaauwen JL, Newman M, Davidson RG. The diplacanthid fishes (Acanthodii, Diplacanthiformes, Diplacanthidae) from the Middle Devonian of Scotland. Palaeontologica Electronica. 2016;19(1.10A):1–83.

2. Zhu M, Yu X, Ahlberg PE, Choo B, Lu J, Qiao T, et al. A Silurian placoderm with osteichthyan-like marginal jaw bones. Nature. 2013;502(7470):188–193.

3. Donoghue PCJ, Smith MP. The anatomy of *Turinia pagei* (Powrie), and the phylogenetic status of the Thelodonti. Earth and Environmental Science Transactions of the Royal Society of Edinburgh. 2001;92(01):15–37. doi:doi:10.1017/S026359330000002X.

4. Janvier P, Arsenault M, Desbiens S. Calcified cartilage in the paired fins of the osteostracan *Escuminaspis laticeps* (Traquair 1880), from the Late Devonian of Miguasha (Québec, Canada), with a consideration of the early evolution of the pectoral fin endoskeleton in vertebrates. Journal of Vertebrate Paleontology. 2004;24(4):773–779. doi:10.2307/4524773.

5. Qu Q, Blom H, Sanchez S, Ahlberg P. Three-dimensional virtual histology of silurian osteostracan scales revealed by synchrotron radiation microtomography. Journal of Morphology. 2015;276(8):873–888. doi:10.1002/jmor.20386.

6. Wang X, Miao D, Zhang Y. Cannibalism in a semi-aquatic reptile from the Early Cretaceous of China. Chinese Science Bulletin. 2005;50(3):282–284. doi:10.1007/bf02897540.

7. Gross W. Downtonische und dittonische Acanthodier-reste des Ostseegebietes. Palaeontographica Abteilung A Palaeozoologie-Stratigraphie. 1971;136:1–82.

8. Valiukevičius J. Acanthodian histology: Some significant aspects in taxonomical and phylogenetical research. Geobios. 1995;28, Supple(0):157–159. doi:10.1016/s0016-6995(95)80105-7.

9. Hawthorn JR, Wilson MVH, Falkenberg AB. Development of the dermoskeleton in *Superciliaspis gabrielsei* (Agnatha: Osteostraci). Journal of Vertebrate Paleontology. 2008;28(4):951–960.

10. Qu Q, Sanchez S, Zhu M, Blom H, Ahlberg PE. The origin of novel features by changes in developmental mechanisms: ontogeny and three-dimensional microanatomy of polyodontode scales of two early osteichthyans. Biological Reviews. 2016;.

11. Brazeau MD. The braincase and jaws of a Devonian "acanthodian" and modern gnathostome origins. Nature. 2009;457(15):305–308.

12. Burrow CJ, Turner S. A review of placoderm scales, and their significance in placoderm phylogeny. Journal of Vertebrate Paleontology. 1999;19(2):204–219.

13. Keating JN, Donoghue PCJ. Histology and affinity of anaspids, and the early evolution of the vertebrate dermal skeleton. Proceedings of the Royal Society of London B: Biological Sciences. 2016;283(1826).

14. Hanke GF. *Promesacanthus eppleri* n. gen., n. sp., a mesacanthid (Acanthodii, Acanthodiformes) from the Lower Devonian of northern Canada. Geodiversitas. 2008;30(2):287–302.

15. Kemp NE, Westrin SK. Ultrastructure of calcified cartilage in the endoskeletal tesserae of sharks. Journal of Morphology. 1979;160(1):75–101.

16. Gagnier PY. Acanthodii. In: Schultze HP, Cloutier R, editors. Devonian Fishes and Plants of Miguasha, Quebec, Canada. München: Verlag Dr. Friedrich Pfeil; 1996. p. 149–164.

17. Arratia G, Cloutier R. Reassessment of the morphology of *Cheirolepis canadensis* (Actinopterygii). In: Schultze HP, Cloutier R, editors. Devonian Fishes and Plants of Miguasha, Quebec, Canada. München: Verlag Dr. Friedrich Pfeil; 1996. p. 165–197.

18. Arratia G, Cloutier R. A new cheirolepidid fish from the Middle-Upper Devonian of Red Hill, Nevada, USA. In: Arratia G, Wilson MVH, Cloutier R, editors. Recent Advances in the Origin and Early Radiation of Vertebrates. München, Germany: Verlag Dr. Friedrich Pfeil; 2004. p. 583–598.

19. Andrews M, Long J, Ahlberg P, Barwick R, Campbell K. The structure of the sarcopterygian *Onychodus jandemarrai* n. sp. from Gogo, Western Australia: With a functional interpretation of the skeleton. Transactions of the Royal Society of Edinburgh: Earth Sciences. 2005;96(03):197–307.

20. Cloutier R, Ahlberg PE. Morphology, characters and the interrelationships of basal sarcopterygians. In: Stiassny MLJ, Parenti LR, Johnson GD, editors. Interrelationships of Fishes. USA: Academic Press Inc.; 1996. p. 445–469.

21. Schultze HP, Cumbaa SL. *Dialipina* and the characters of basal actinopterygians . In: Ahlberg PE, editor. Major Events in Early Vertebrate Evolution. London: Taylor & Francis; 2001. p. 315–332.

22. Cloutier R, Arratia G. Early diversification of actinopterygians. In: Arratia G, Wilson MVH, Cloutier R, editors. Recent Advances in the Origin and Early Radiation of Vertebrates. München: Verlag Dr. Friedrich Pfeil; 2004. p. 217–270.

23. Giles S, Coates MI, Garwood RJ, Brazeau MD, Atwood R, Johanson Z, et al. Endoskeletal structure in *Cheirolepis* (Osteichthyes, Actinopterygii), An early ray-finned fish. Palaeontology. 2015; p. n/a–n/a. doi:10.1111/pala.12182.

24. Warren A, Currie BP, Burrow C, Turner S. A redescription and reinterpretation of *Gyracanthides murrayi* Woodward 1906 (Acanthodii, Gyracanthidae) from the Lower Carboniferous of the Mansfield Basin, Victoria, Australia. Journal of Vertebrate Paleontology. 2000;20(2):225–242.

25. Schultze HP. Ausgangsform und Entwicklung der rhombischen Schuppen der Osteichthyes (Pisces). Paläontologische Zeitschrift. 1977;51(3-4):152–168.

26. Béchard I, Arsenault F, Cloutier R, Kerr J. The Devonian placoderm fish *Bothriolepis canadensis* revisited with three-dimensional digital imagery. Palaeontologia Electronica. 2014;17(1):1–19.

27. Zidek J. Kansas Hamilton Quarry (Upper Pennsylvanian) *Acanthodes*, with remarks on the previously reported North American occurrences of the genus. The University of Kansas Paleontological contributions. 1976;83.

28. Hanke GF, Wilson MVH. Anatomy of the Early Devonian acanthodian *Brochoadmones milesi* based on nearly complete body fossils, with comments on the evolution and development of paired fins. Journal of Vertebrate Paleontology. 2006;26(3):526–537.

29. Newman MJ, Burrow CJ, Den Blaauwen JL, Davidson RG. The Early Devonian acanthodian *Euthacanthus macnicoli* Powrie, 1864 from the Midland Valley of Scotland. Geodiversitas. 2014;36(2):321–348.

30. Watson DMS. The acanthodian fishes. Philosophical Transactions of the Royal Society of London Series B, Biological Sciences. 1937;228(549):49–146.

31. Wilson LB, Schradin C, Mitgutsch C, Galliari F, Mess A, Sánchez-Villagra M. Skeletogenesis and sequence heterochrony in rodent evolution, with particular emphasis on the African striped mouse, *Rhabdomys pumilio* (Mammalia). Organisms Diversity & Evolution. 2010;10(3):243–258.

32. Hanke GF, Wilson MVH. New teleostome fishes and acanthodian systematics. In: Arratia G, Wilson MVH, Cloutier R, editors. Recent Advances in the Origin and Early Radiation of Vertebrates. München: Verlag Dr Friedrich Pfeil; 2004. p. 189–216.

33. Hanke GF, Davis SP, Wilson MVH. New species of the acanthodian genus *Tetanopsyrus* from Northern Canada, and comments on related taxa. Journal of Vertebrate Paleontology. 2001;21(4):740–753. doi:10.1671/0272-4634(2001)021[0740:nsotag]2.0.co;2.

34. Gagnier PY, Wilson MVH. Early Devonian acanthodians from northern Canada. Palaeontology. 1996;39(2):241–258.

35. Long JA. A new diplacanthoid acanthodian from the Late Devonian of Victoria. Memoirs of the Association of Australasian Palaeontologists. 1983;1:51–65.

36. Burrow CJ, Rudkin D. Oldest near-complete acanthodian: The first vertebrate from the Silurian Bertie Formation Konservat-Lagerstätte, Ontario. PLoS ONE. 2014;9(8):e104171. doi:10.1371/journal.pone.0104171.

37. Brazeau MD. A revision of the anatomy of the Early Devonian jawed vertebrate *Ptomacanthus anglicus* Miles. Palaeontology. 2012;55(2):355–367. doi:10.1111/j.1475-4983.2012.01130.x.

38. Friedman M, Matt F. *Styloichthys* as the oldest coelacanth: Implications for early osteichthyan relationships. Journal of Systematic Palaeontology. 2007;5(3):289–343. doi:10.1017/S1477201907002052.

39. Forey PL, Ahlberg PE, Lukševičs E, Zupinš I. A new coelacanth from the Middle Devonian of Latvia. Journal of Vertebrate Paleontology. 2000;20(2):243–252.

40. Porro LB, Rayfield EJ, Clack JA. Computed tomography, anatomical description and three-dimensional reconstruction of the lower jaw of *Eusthenopteron foordi* Whiteaves, 1881 from the Upper Devonian of Canada. Palaeontology. 2015; p. n/a–n/a. doi:10.1111/pala.12192.

41. Karatajute-Talimaa VN. The early stage of the dermal skeleton formation in chondrichthyans. In: Mark-Kurik E, editor. Fossil Fishes as Living Animals. Tallinn: Academy of Sciences of Estonia; 1992. p. 223–231.

42. Sansom RS, Rodygin SA, Donoghue PCJ. The anatomy, affinity and phylogenetic significance of *Ilemoraspis kirkinskayae* (Osteostraci) from the Devonian of Siberia. Journal of Vertebrate Paleontology. 2008;28(3):613–625.

43. Burrow CJ, Davidson RG, Den Blaauwen JL, Newman MJ. Revision of *Climatius reticulatus* Agassiz, 1844 (Acanthodii, Climatiidae), from the Lower Devonian of Scotland, based on new histological and morphological data. Journal of Vertebrate Paleontology. 2015; p. e913421. doi:10.1080/02724634.2014.913421.

44. Burrow CJ, Newman MJ, Davidson RG, Den Blaauwen JL. Redescription of *Parexus recurvus*, an Early Devonian acanthodian from the Midland Valley of Scotland. Alcheringa. 2013;37:1–23.

45. Hanke GF, Wilson MVH. The putative stem-group chondrichthyans *Kathemacanthus* and *Seretolepis* from the Lower Devonian MOTH locality, Mackenzie Mountains, Canada. In: Elliott DK, Maisey JG, Yu X, Miao D, editors. Morphology, Phylogeny and Paleobiogeography of Fossil Fishes. München: Dr Friedrich Pfeil; 2010. p. 159–182.

46. Sansom RS, Freedman KIM, Gabbott SE, Aldridge RJ, Purnell MA. Taphonomy and affinity of an enigmatic Silurian vertebrate, *Jamoytius kerwoodi* White. Palaeontology. 2010;53(6):1393–1409. doi:10.1111/j.1475-4983.2010.01019.x.

47. Choo B. Revision of the actinopterygian genus *Mimipiscis* (= *Mimia*) from the Upper Devonian Gogo Formation of Western Australia and the interrelationships of the early Actinopterygii. Earth and Environmental Science Transactions of the Royal Society of Edinburgh. 2011;102(02):77–104.

48. Schultze HP. Scales, enamel, cosmine, ganoine, and early osteichthyans. Comptes Rendus Palevol. 2015;(0). doi:http://dx.doi.org/10.1016/j.crpv.2015.04.001.

49. Davis SP, Finarelli JA, Coates MI. *Acanthodes* and shark-like conditions in the last common ancestor of modern gnathostomes. Nature. 2012;486(7402):247–250.
