# Supplementary figures and images for: From body scale ontogeny to species ontogeny: Histological and morphological assessment of the Late Devonian acanthodian Triazeugacanthus affinis from Miguasha, Canada
Source: PLoS One. 2017 Apr 12;12(4):e0174655. doi: 10.1371/journal.pone.0174655 (PMC5389634; doi:10.1371/journal.pone.0174655)

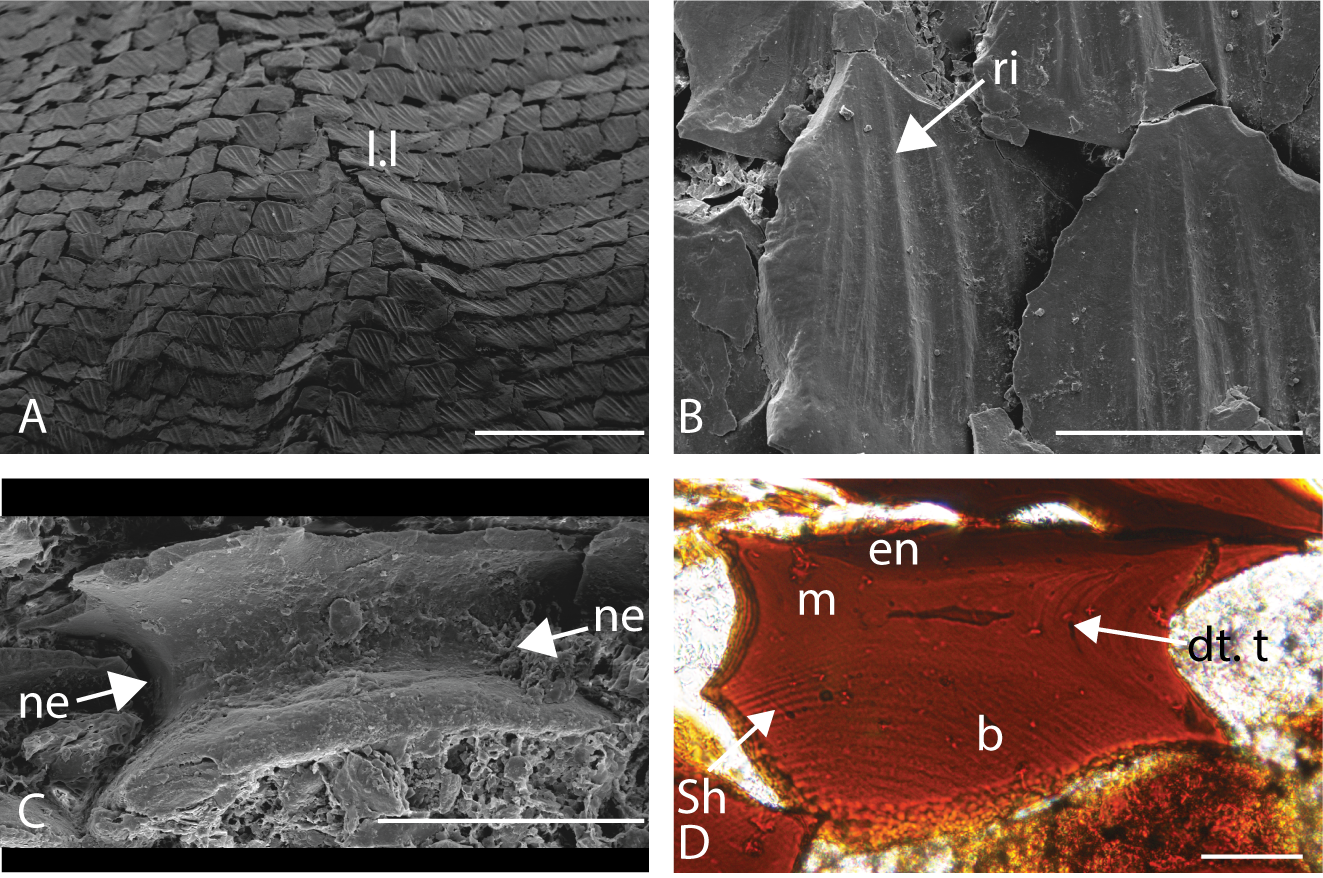

Supplement: S1 Fig — A-D: MHNM 03-2215. A: SEM of the body squamation showing the alignment. B: Details from two scales showing the superficial ridges. C: SEM observations of a transverse section. D: Transverse ground section under polarised light. b, acellular bone; m, mesodentine; en, well-mineralised layer; l.l, lateral line; ne, neck; ri, superficial ridge; Sh, Sharpey’s fibers. Scale bar = 1 mm in A, 200 μm in B, 100 μm in C, 50 μm in D. (TIF) [file pone.0174655.s007.tif]

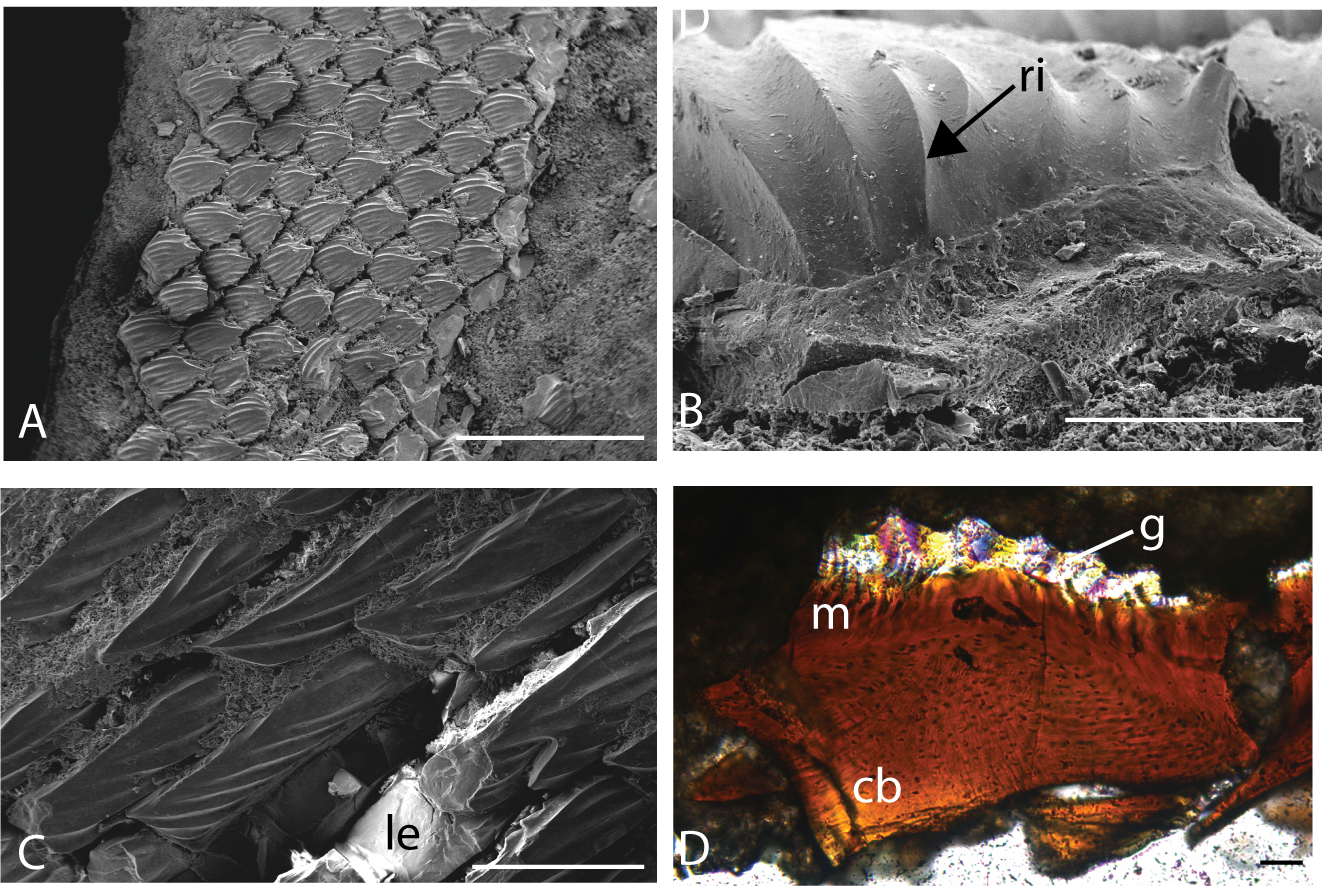

Supplement: S2 Fig — A: MHNM 05-53, SEM of the body squamation. B: MHNM 05-53, details showing the ridged scale surface and the broad base. C: MHNM 05-152, SEM of lepidotrichial segments showing the ornamentation. D: Transverse ground section of a scale under polarised light. cb, cellular bone; m, mesodentine; g, ganoine; le, lepidotrichium; ri, superficial ridge. Scale bar = 2 mm in A, 200 μm in B, 500 μm in C, 50 μm in D. (TIF) [file pone.0174655.s008.tif]

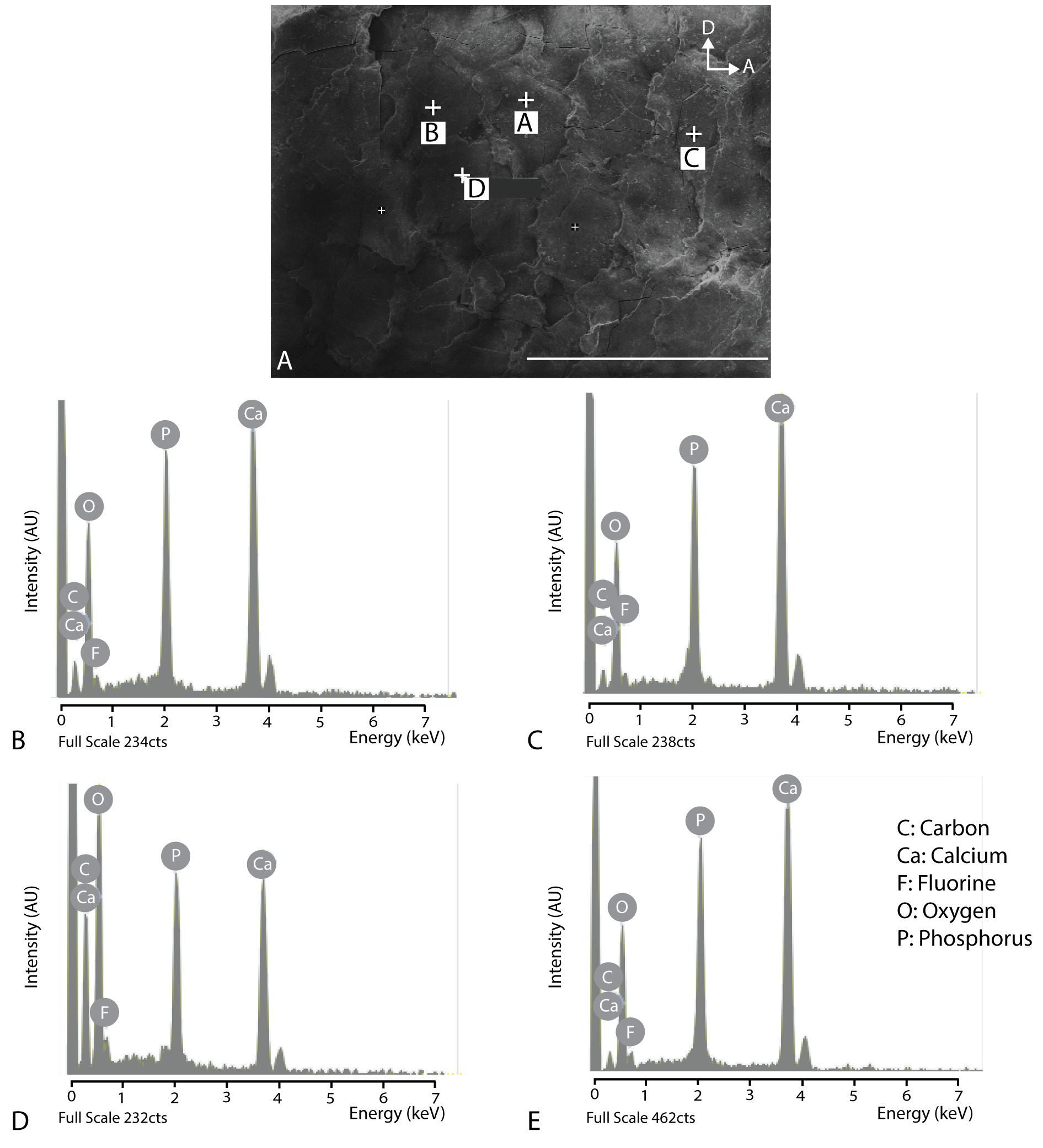

Supplement: S3 Fig — A: MHNM 03-1497, location of spectra for EDX analyses. Note that the oxygen peak is non-significant and depends essentially on the vacuum level in the chamber of the environmental SEM. Scale bar: A = 1 mm. (TIF) [file pone.0174655.s009.tif]

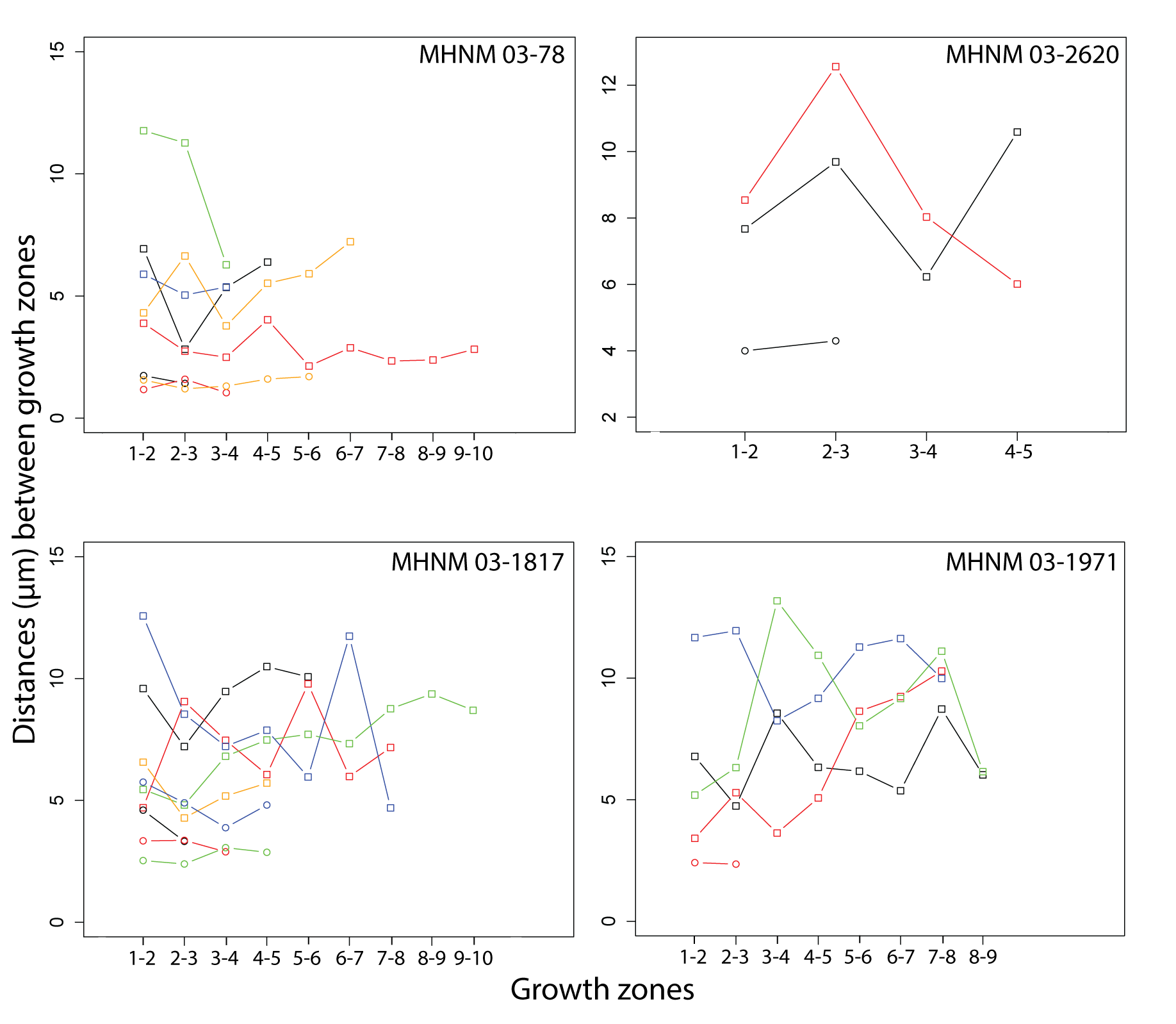

Supplement: S4 Fig — Circles are for the ganoine layers (superimpositional growth), squares for mesodentine and bone layers (“box-in-box” growth). The “box-in-box” patttern of growth is more irregular than the superimpositional growth of the multi-layered ganoine. Growth zones are numbered by the two growth lines that delimit the zone. Each coloured line represents one scale. (TIF) [file pone.0174655.s010.tif]

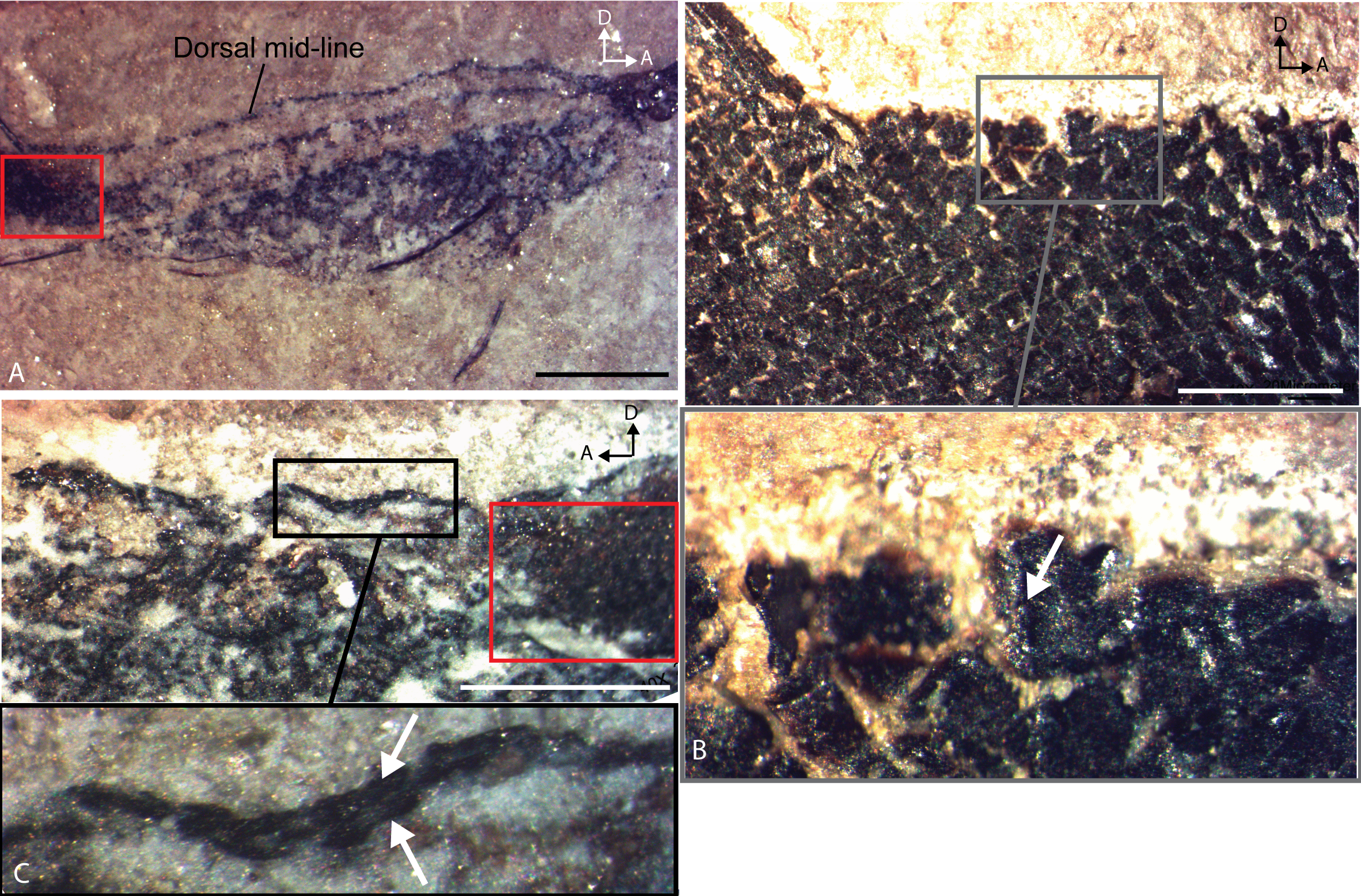

Supplement: S5 Fig — A: Early juvenile, MHNM 03-1252. Dorsal mid-line scales develop before trunk scales; the latter are only present in the posterior region (red rectangle). B: Adult, MHNM 03-1497. Dorsal scale showing the presence of a median ridge (white arrow). C: Late juvenile, MHNM 03-2684. Two parallel scale rows (white arrows) are present anterior to trunk scales (red rectangle). Scale bars: A = 2 mm, B, C = 1mm. (TIF) [file pone.0174655.s011.tif]

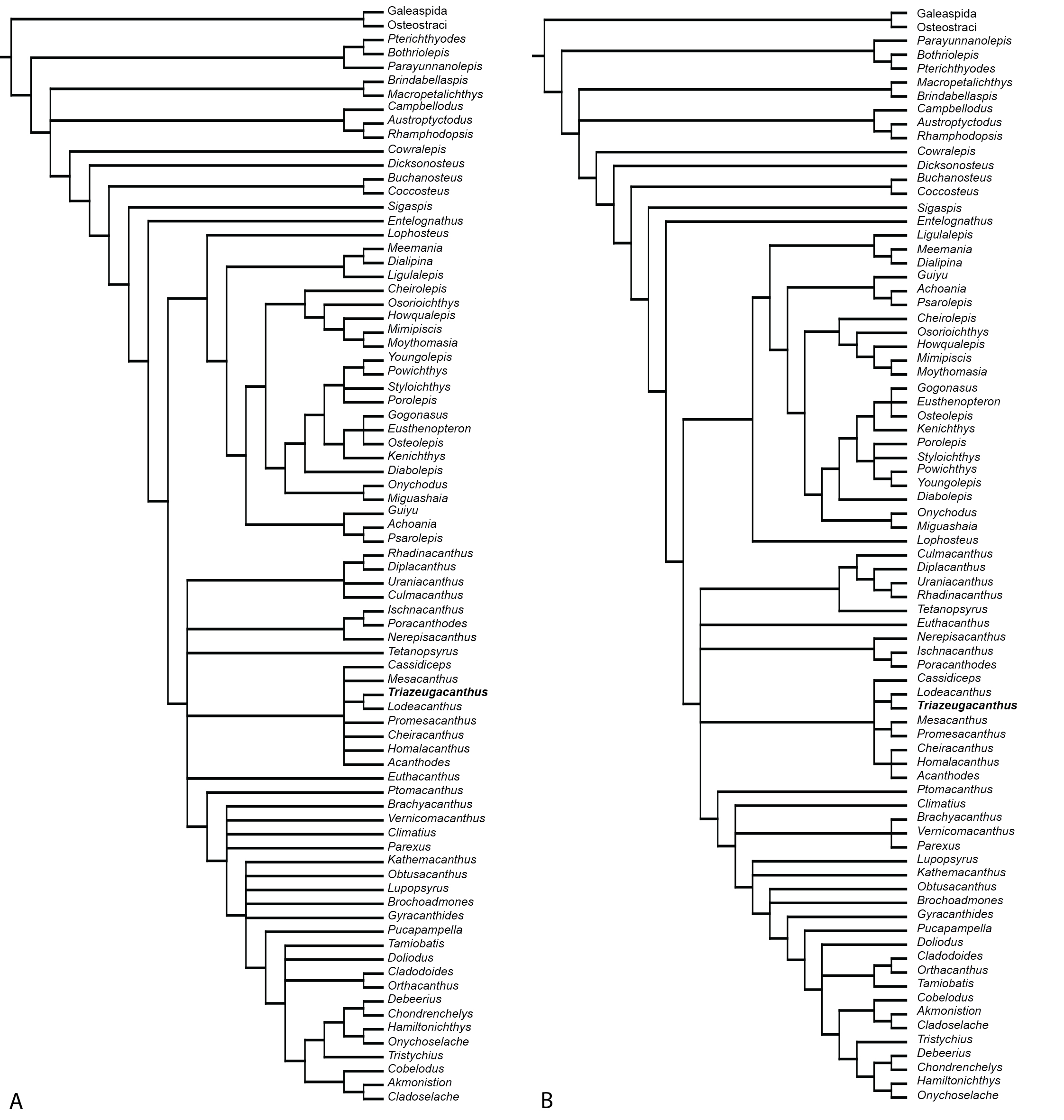

Supplement: S6 Fig — A: Strict consensus of 10,000 most parsimonious trees (711 steps). B: Adams consensus of 10,000 most parsimonious trees (711 steps). (JPG) [file pone.0174655.s012.jpg]

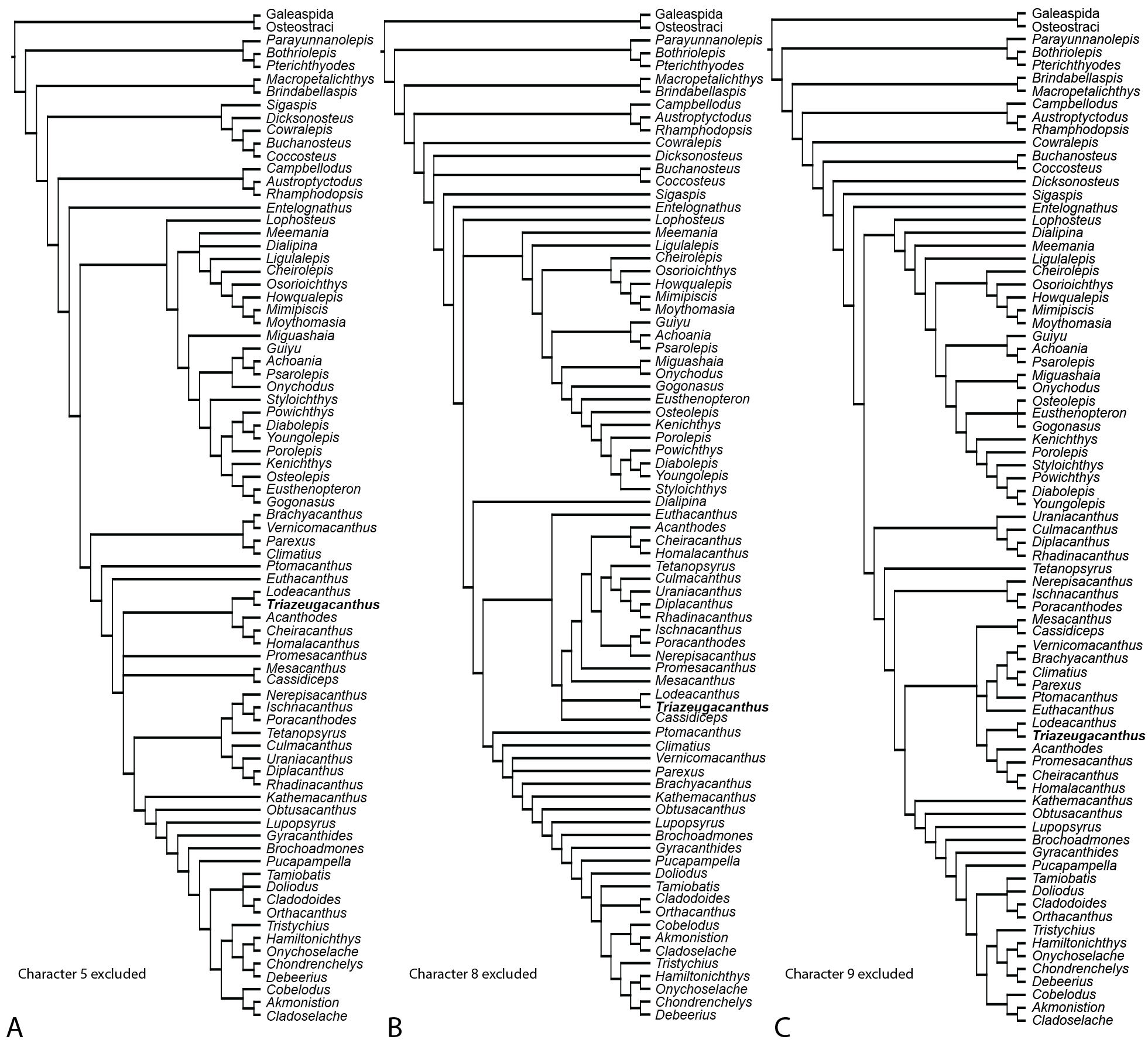

Supplement: S7 Fig — Each analysis is realized with the exclusion of one scale-related character. (JPG) [file pone.0174655.s013.jpg]

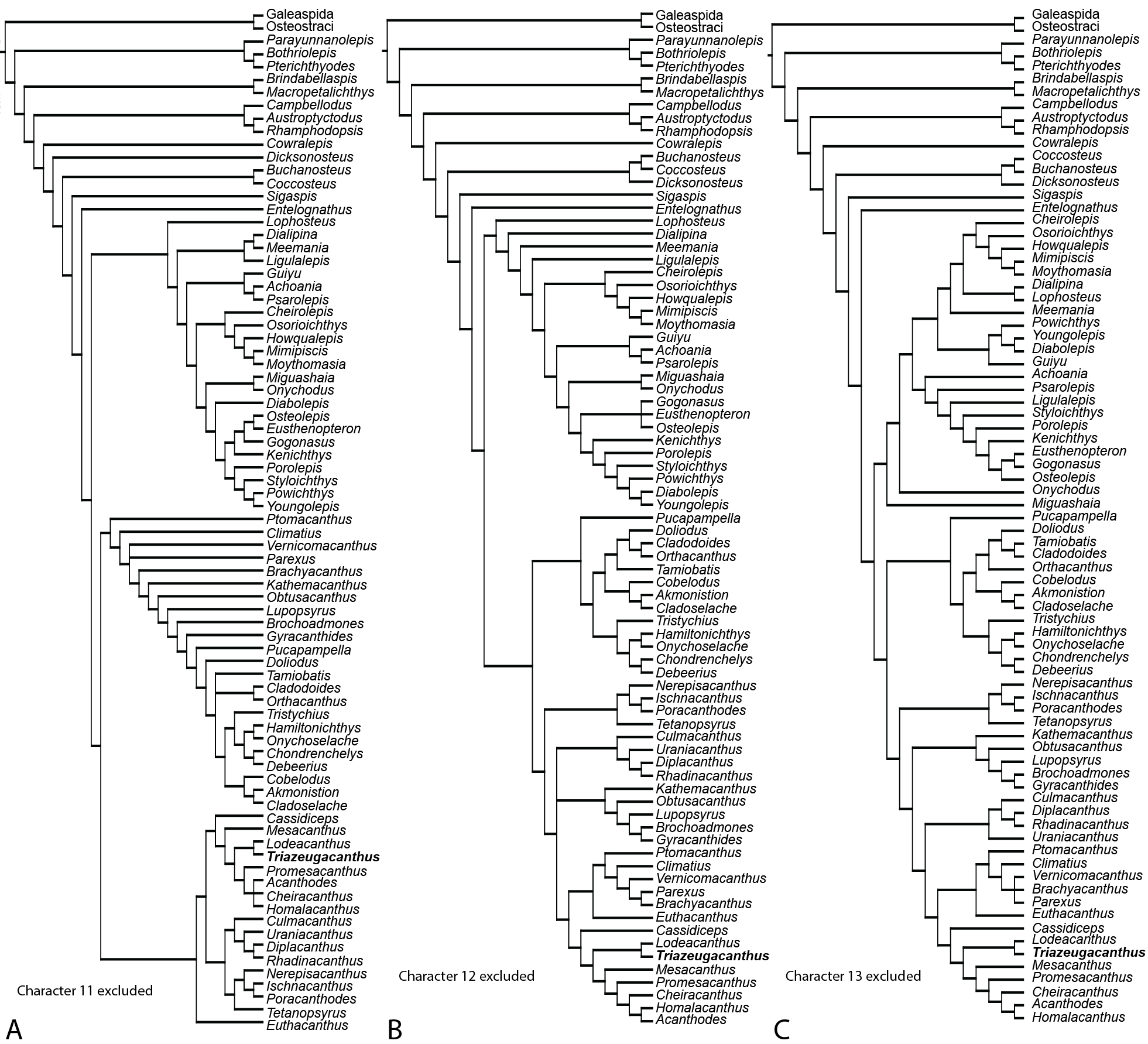

Supplement: S8 Fig — Each analysis is realized with the exclusion of one scale-related character. (JPG) [file pone.0174655.s014.jpg]

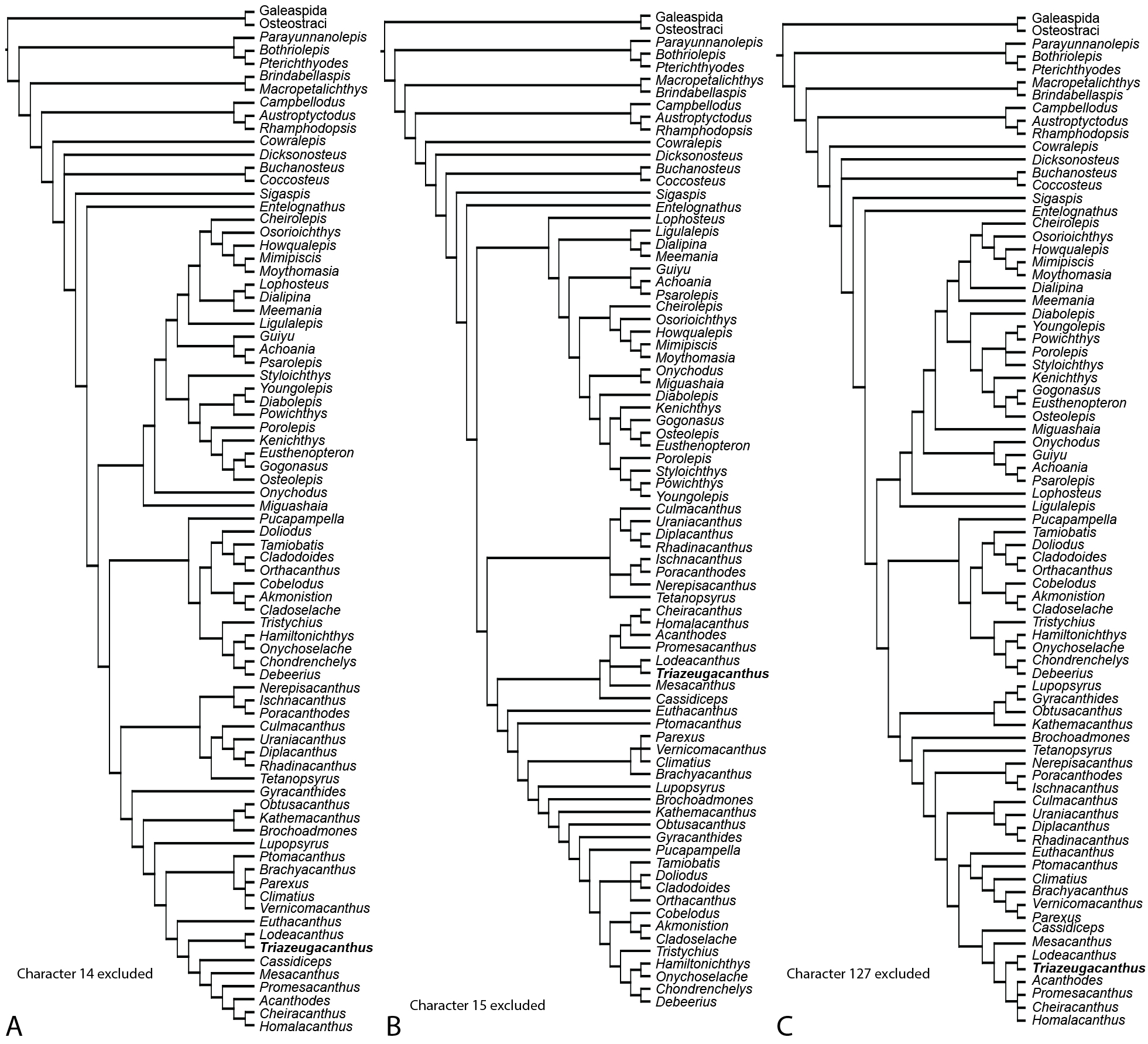

Supplement: S9 Fig — Each analysis is realized with the exclusion of one scale-related character. (JPG) [file pone.0174655.s015.jpg]

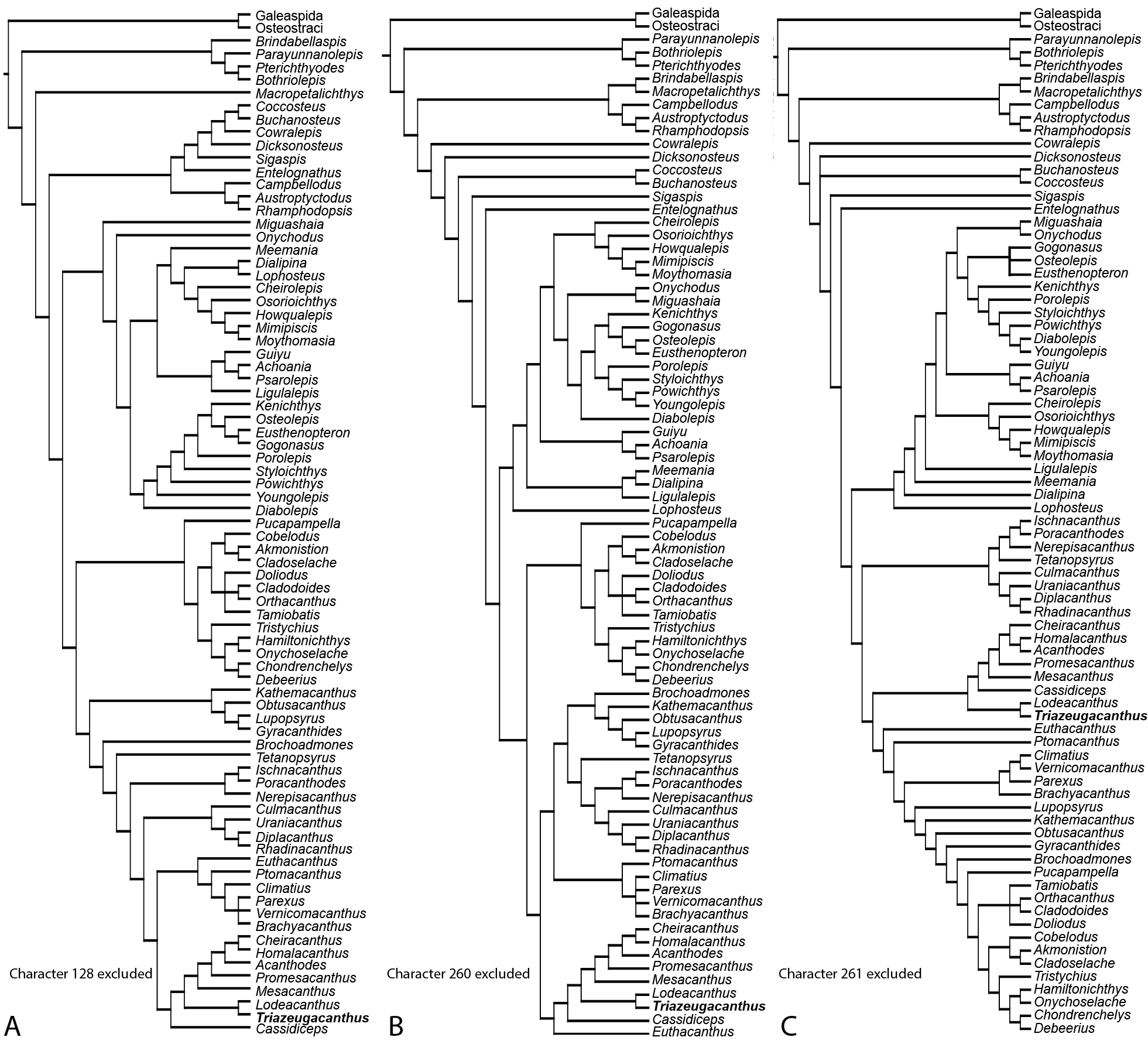

Supplement: S10 Fig — Each analysis is realized with the exclusion of one scale-related character. (JPG) [file pone.0174655.s016.jpg]

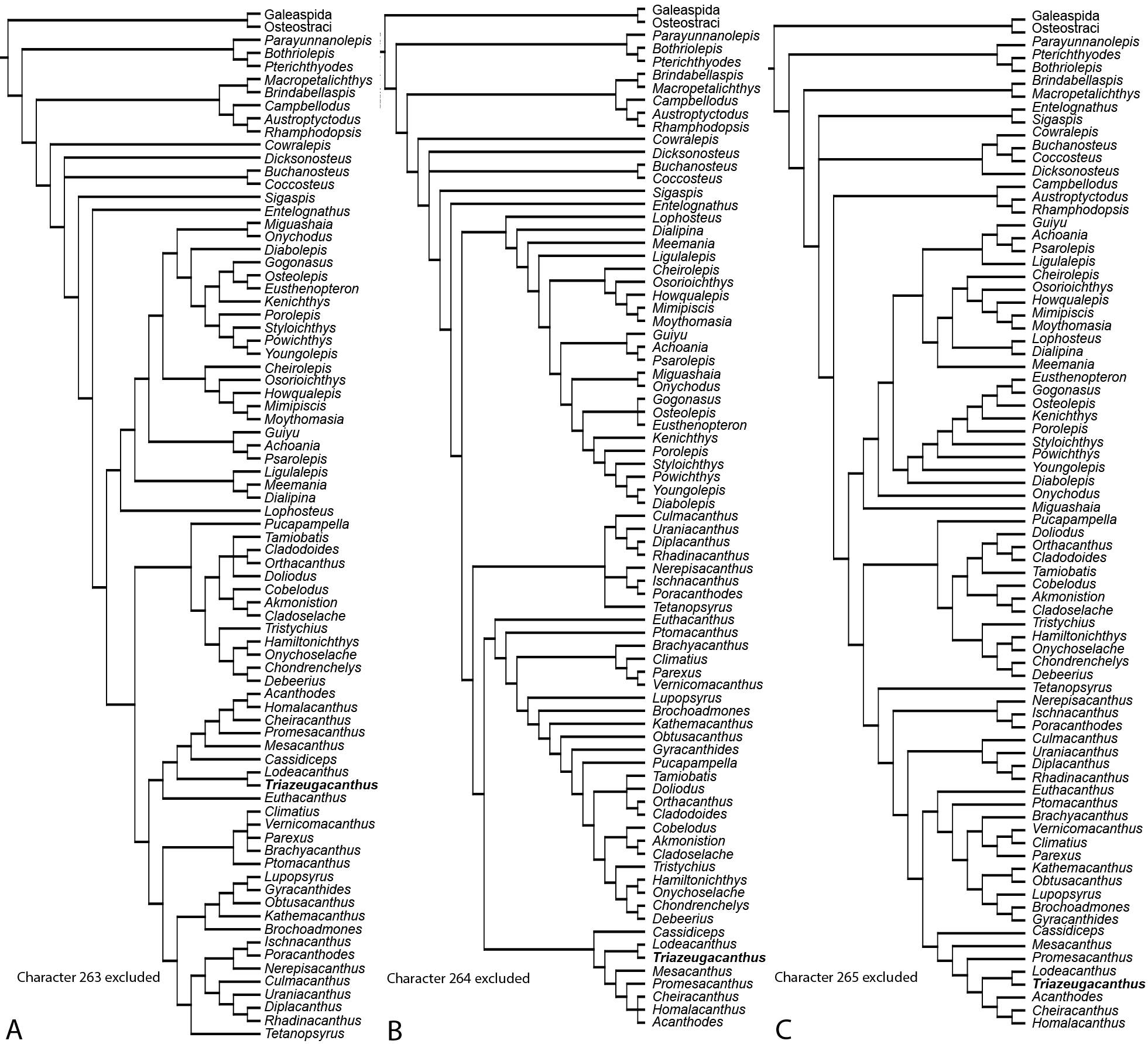

Supplement: S11 Fig — Each analysis is realized with the exclusion of one scale-related character. (JPG) [file pone.0174655.s017.jpg]

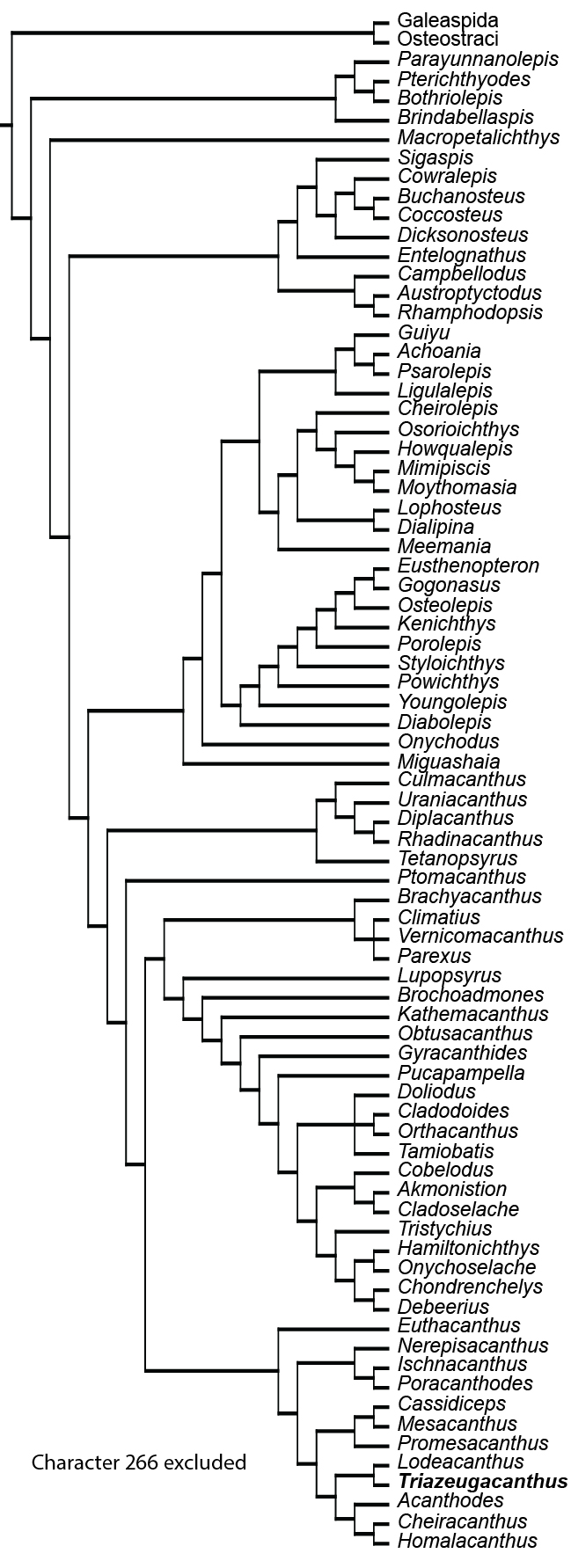

Supplement: S12 Fig — The analysis is realized with the exclusion of one scale-related character. (JPG) [file pone.0174655.s018.jpg]
